# The genome sequence and transcriptome of *Potentilla micrantha* and their comparison to *Fragaria vesca* (the woodland strawberry)

**DOI:** 10.1093/gigascience/giy010

**Published:** 2017-02-15

**Authors:** Matteo Buti, Marco Moretto, Elena Barghini, Flavia Mascagni, Lucia Natali, Matteo Brilli, Alexandre Lomsadze, Paolo Sonego, Lara Giongo, Michael Alonge, Riccardo Velasco, Claudio Varotto, Nada Šurbanovski, Mark Borodovsky, Judson A Ward, Kristof Engelen, Andrea Cavallini, Alessandro Cestaro, Daniel James Sargent

**Affiliations:** 1 Department of Genomics and Biology of Fruit Crops, Research and Innovation Centre, Fondazione Edmund Mach (FEM) Via E. Mach 1, 38010 San Michele all'Adige, Italy; 2 Center for the Development and Improvement of Agri-Food Resources (BIOGEST-SITEIA) University of Modena and Reggio Emilia, P.le Europa 1, 42124 Reggio nell'Emilia (RE), Italy; 3 Unit of Computational Biology, Research and Innovation Centre, Fondazione Edmund Mach (FEM) Via E. Mach 1, 38010 San Michele all'Adige, Italy; 4 Department of Agricultural, Food, and Environmental Sciences, University of Pisa, Pisa I-56124, Italy; 5 Department of Agronomy, Food, Natural Resources, Animals and Environment, University of Padova Agripolis, V.le dell'Università 16, 35020 Legnaro (PD), Italy; 6 Dipartimento di Bioscienze e Centro di Ricerca Pediatrica Romeo ed Enrica Invernizzi, Università degli Studi di Milano, Via Celoria 26, 20133 Milano; 7 Wallace H. Coulter Department of Biomedical Engineering, Georgia Tech, Atlanta, Georgia; 8 Driscoll's Strawberry Associates, Cassin Ranch, 121 Silliman Drive, Watsonville, California; 9 Department of Biodiversity and Molecular Ecology, Research and Innovation Centre, Fondazione Edmund Mach (FEM) Via E. Mach 1, 38010 San Michele all'Adige, Italy; 10 Driscoll's Genetics Limited, East Malling Enterprise Centre, New Road, East Malling, Kent ME19 6BJ, UK

**Keywords:** long-read sequencing, evolutionary development, angiosperms, genome sequence, transcriptomics

## Abstract

**Background:**

The genus *Potentilla* is closely related to that of *Fragaria*, the economically important strawberry genus. *Potentilla micrantha* is a species that does not develop berries but shares numerous morphological and ecological characteristics with *Fragaria vesca*. These similarities make *P. micrantha* an attractive choice for comparative genomics studies with *F. vesca*.

**Findings:**

In this study, the *P. micrantha* genome was sequenced and annotated, and RNA-Seq data from the different developmental stages of flowering and fruiting were used to develop a set of gene predictions. A 327 Mbp sequence and annotation of the genome of *P. micrantha*, spanning 2674 sequence contigs, with an N50 size of 335,712, estimated to cover 80% of the total genome size of the species was developed. The genus *Potentilla* has a characteristically larger genome size than *Fragaria*, but the recovered sequence scaffolds were remarkably collinear at the micro-syntenic level with the genome of *F. vesca*, its closest sequenced relative. A total of 33,602 genes were predicted, and 95.1% of bench-marking universal single-copy orthologous genes were complete within the presented sequence. Thus, we argue that the majority of the gene-rich regions of the genome have been sequenced.

**Conclusions:**

Comparisons of RNA-Seq data from the stages of floral and fruit development revealed genes differentially expressed between *P. micrantha* and *F. vesca*.The data presented are a valuable resource for future studies of berry development in *Fragaria* and the Rosaceae and they also shed light on the evolution of genome size and organization in this family.

## Background

*Potentilla*, a genus of approximately 500 species [1], is closely related to that of *Fragaria* [2], the genera having diverged from a common ancestor just 24 million years ago [3]. The genus *Fragaria*, a member of the Fragariianae tribe of the Rosaceae family, is economically important due to the sweet, aromatic accessory fruits (berries) produced by members of the genus, in particular, those of the cultivated allo-octoploid (2*n* = 8 *×* = 56) strawberry species *F. × ananassa*. The availability of a genome sequence for a wild diploid relative of the cultivated strawberry, the woodland strawberry *Fragaria vesca* (2*n* = 2 *×* = 14) [4], has enabled the investigation of the molecular basis of many traits of economic and academic interest in strawberry, including the development of accessory fruits. However, all members of the *Fragaria* genus produce berries; as such, the use of reverse genetics approaches to study the genes involved in berry evolution and development would require *Fragaria* mutants that do not produce fruits, a resource that is not currently available.

In the post genomics era, comparative analysis permits the study of related, yet divergent, species by tracing changes at the genomic and transcriptomic levels responsible for their phenotypic differences. Previously, the sequenced genomes of *F. vesca*, *Prunus persica*, and *Malus × domestica* were compared [5], providing insights into the evolutionary mechanisms that have shaped the three species and demonstrating that the *Fragaria* genome underwent significant small-scale structural rearrangements since it diverged from the common ancestor of the three genera. Comparative transcriptomics can also be used to reveal differences in the expression of orthologous genes between organisms at different stages of physiological development [6]. Such an approach suggests that comparative analyses between *Fragaria* and a closely related species that does not bear berries may reveal important insights into the evolution of fruit development. Additionally, speciation is often related to changes in genome structure and genome size, in particular. Differences in genome size are often the consequence of polyploidization events and/or changes in the abundance of repetitive DNA, especially transposable elements [7].

**Figure 1:**
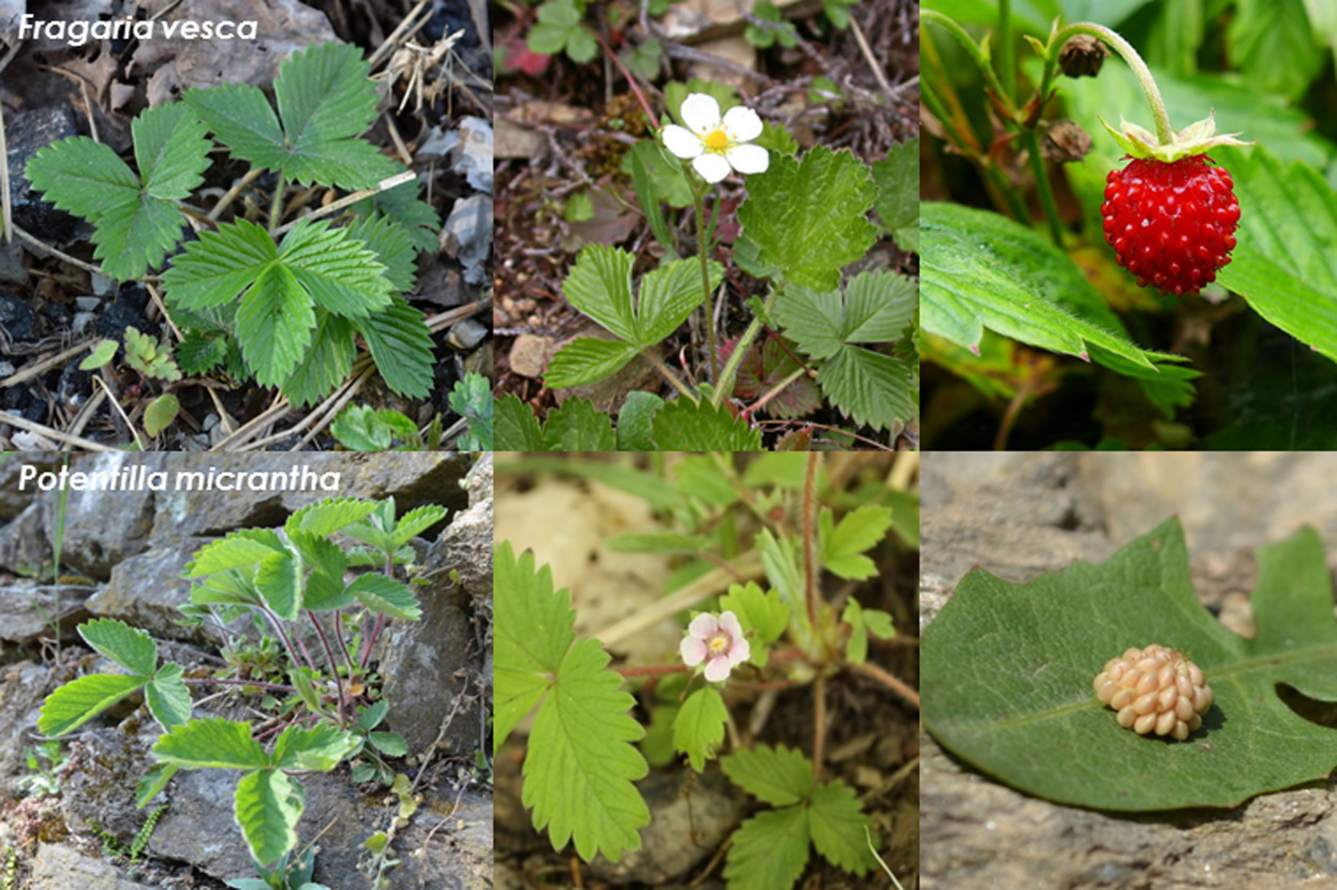
Comparison of *Fragaria vesca* and *Potentilla micrantha* morphology for leaves, flowers, and fruits.

*Potentilla micrantha*, like the majority of species of the genus *Potentilla*, does not develop accessory fruits; however, it shares numerous morphological characteristics with *F. vesca* (Fig. 1), including plant habit and flower morphology. Notably, they grow within the same ecological niches and, where their ranges of distribution overlap, *P. micrantha* can be found growing nearby populations of *F. vesca* (Sargent, unpublished results). These striking similarities make *P. micrantha* an attractive choice for understanding the genetic basis of berry development in *F. vesca*. As a precursor to a whole genome sequencing initiative, an initial sequencing project focused on the *P. micrantha* chloroplast was undertaken using the Illumina HiSeq and PacBio RS sequencing platforms [8].

## Data Description

Our objectives in this study were to develop a genomic toolkit for *P. micrantha* to permit comparative genomic and transcriptomic studies with *F. vesca*, with a view to identifying the evolutionary changes that have occurred between the two species. The genome size of *P. micrantha* was determined using flow cytometry. The nuclear genome was sequenced and assembled from Illumina and PacBio sequencing reads and assembled and integrated using ALLPATHS and PBJelly. Gene predictions from the *P. micrantha* genome were made with support of RNA-Seq data generated from tissue libraries sampled during flower and fruit development. The genome of *F. vesca* was compared to the sequencing scaffolds produced for *P. micrantha*, and while they exhibited a remarkable degree of collinearity at the micro-syntenic level, large-scale differences in transposon activity were identified that might explain the large differences in genome size between the two species. The dataset we report will be useful for comparative studies of a number of traits between *P. micrantha* and its economically important close relatives.

### Flow cytometry, heterozygosity estimation, and genome assembly

DNA was extracted from *P. micrantha* young, unexpanded leaves. Flow cytometry using a *V. minor* internal standard with a DNA content of 1.52 pg/2C returned average DNA quantities of 0.52 pg/2C for *F. vesca* “Hawaii 4” and 0.83 pg/2C for *P. micrantha* over 3 biological replicates. Using the calculation of Dolezel et al. [9] that 1 pg DNA is equivalent to 978 Mbp of DNA sequence, the genome size of *P. micrantha* was determined as 405.87 Mbp in length and that of *F. vesca* “Hawaii 4” was calculated to be 254.28 Mbp.

Data were returned for the overlapping fragment library (OLF), and all 4 mate-pair libraries were sequenced using Illumina HiSeq. In total, 61.4 Gbp of data were returned, and the relative depth of coverage obtained for the *P. micrantha* genome from each library is given in Additional File 1, Table S1. Four different PacBio RS sequencing libraries were constructed and sequenced using 2 different versions of the PacBio chemistry (Additional File 2, Table S2). From the sequencing of 63 single molecule real-time (SMRT) cells, 6,447,413 sequences with an average length of 2221 bp were recovered, totaling 14.32 Gb of long-read sequence data. From the data, 33 × equivalent of sequence was contained in reads longer than 1 kb, which were used for gap filling of the Illumina assembly using PBJelly [10].

The initial ALLPATHS assembly of the Illumina short-read sequences produced 33,026 contigs with an N50 of 16,235 bp and a total length of 247,565,733 bp. Following scaffolding, a genome assembly with a total length of 315,266,043 bp contained in 2866 sequencing scaffolds was returned. The final scaffold set returned following ALLPATHs assembly contained 0.07% ambiguous sites (single nucleotide polymorphisms), revealing the genome of *P. micrantha* to be one of the most homozygous naturally occurring genomes sequenced to date. Following incorporation of the PacBio RS data using PBJelly [10], the *P. micrantha* sequence assembly contained 326,533,584 bp of sequence data, a 3.5% increase over the ALLPATHS Illumina assembly, in 2674 scaffolds. The longest and N50 scaffold lengths both increased following gap filling by 9.3% and 5.1%, respectively, but most significantly, the number of gapped Ns in the assembly was reduced by 59.7% to 27,311,787 (8.4% of the final assembly) (Table 1). The final scaffolded assembly contained 80.45% of the total estimated genome size for *P. micrantha* as calculated by flow cytometry. Scaffolds ranged from 935 bp to 3,488,351 bp in length. Of the 2674 scaffolds, 878 (32.8%) were less than 10 kbp in length, 534 (20%) were between 10 and 50 kbp in length, 738 (27.6%) were between 50 and 200 kbp in length, 500 (18.7%) contained between 200 kbp and 1 Mbp of sequence, and the remaining 23 (0.9%) contained more than 1 Mbp of sequence. The majority of the 1440 benchmarking single-copy orthologous (BUSCO) groups queried [11] were present in the genome sequence, with 95.1% (1337 complete and single copy and 33 complete and duplicated BUSCOs) identified within the sequencing scaffolds.

**Table 1:** *Potentilla micrantha* assembly stats

	ALLPATHS-LG	
Sequence statistics	Illumina data	PacBio PBJelly
Number of scaffolds	2866	2674 (−6.7%)
Total size of scaffolds	315,266,043	326,533,584 (+3.5%)
Longest scaffold	3,162,838	3,488,351 (+9.3%)
N50 scaffold length	318,490	335,712 (+5.1%)
Gapped Ns in scaffolds	67,706,454	27,311,787 (−59.7%)
Number of contigs	33,026	n/a
Number of contigs in scaffolds	32,063	n/a
Total size of contigs	247,565,733	n/a
N50 contig length	16,235	n/a

### Gene prediction and preliminary annotation

The results of the combined alignment of the 12 RNA-seq read sets to the *P. micrantha* genome assembly and number of splice sites identified using STAR are presented in Additional File 3, Table S3. A total of 1908 consensus repeat sequences were generated by RepeatModeler, totaling 1,431,262 bp and having a G-C content of 40.8%. The total ATCG content of sequencing scaffolds greater than 10 kb in length was 298,987,576 bp. A total of 138,597,969 bp (46.36%) of the genome sequence were masked using the consensus sequences in the RepeatModeler library, including 26,359 (7.5%) of the mapped GT-AG introns identified by STAR. Gene prediction using GeneMark-ET on the masked genome identified 33,602 genes, of which 32,137 were predictions containing multiple exons and 4655 were single exon predictions. A total of 172,791 exons were predicted, with an average length of 223 bp and an average of 5.14 exons per gene. A total of 139,216 introns were predicted in the Coding DNA sequence (CDS) of the genes, with an average intron length of 499 bp. BUSCO analyses were compared between the gene predictions developed for *P. micrantha* and those for *F. vesca*. In total, 1282 (89%) complete and 68 (4.7%) fragmented BUSCOs (93.75% total) were recovered for *P. micrantha* compared to 1303 (90.5%) complete and 79 (5.5%) fragmented BUSCOs (95.6%) recovered for *F. vesca* gene predictions, indicating a similar level of completeness of the *P. micrantha* assembly to its nearest sequenced relative. Following a local Basic Local Alignment Search Tool (BLAST) search and BLAST2GO analysis, 27,968 *P. micrantha* predicted genes were assigned a preliminary gene annotation.

### Scaffold anchoring and synteny to the *Fragaria vesca* Fvb genome sequence

**Figure 2:**
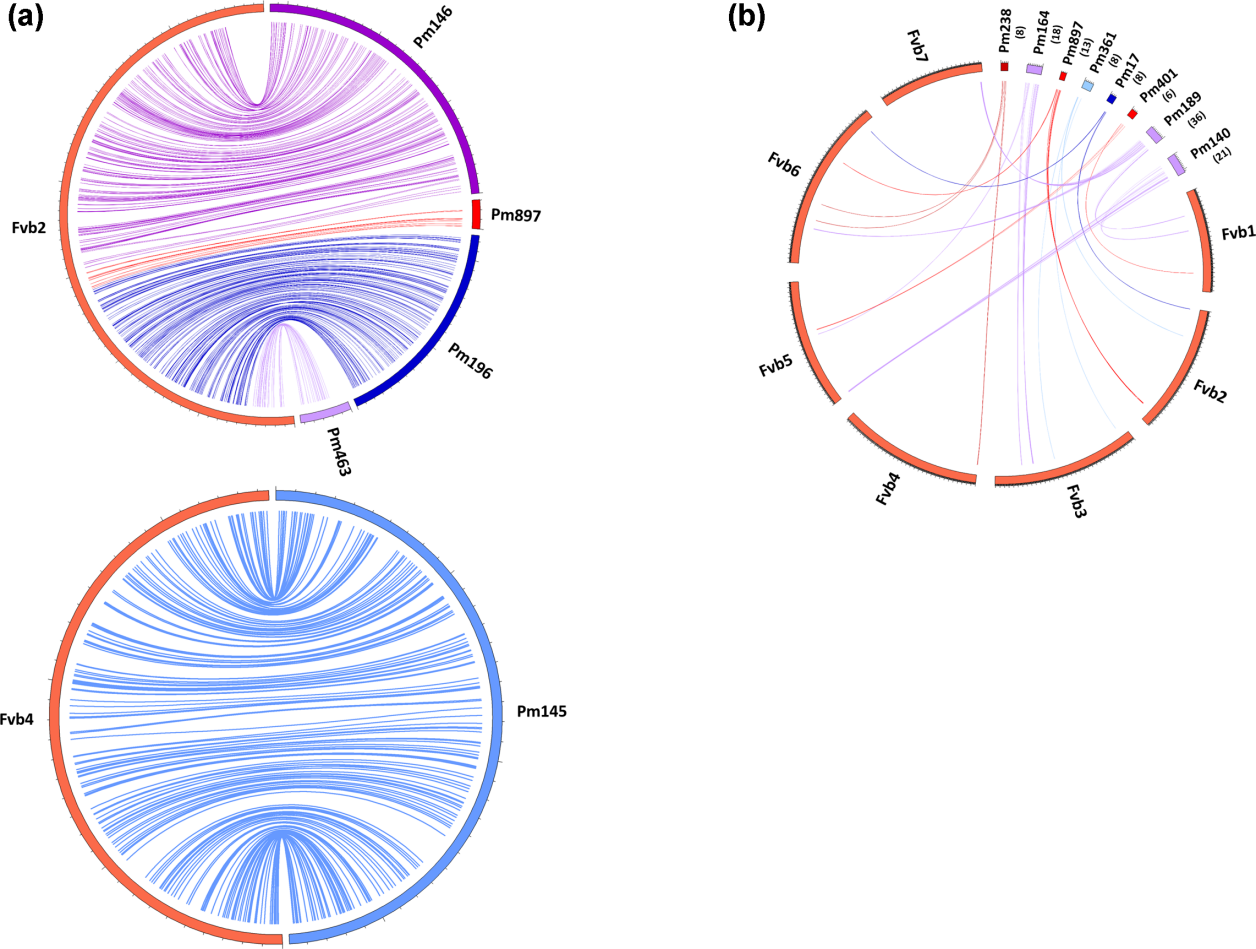
(**a**) Anchoring of 5 *Potentilla micrantha* genome scaffolds to the *Fragaria vesca* Fvb pseudomolecules *Fvb*2 and *Fvb*4 demonstrating the microsynteny between the *F. vesca* and *P. micrantha* genomes (numbers in parentheses below the scaffold names indicate the number of genes contained in each split syntenic block. (**b**) A comparison of the 7 pseudomolecules of the *F. vesca* genome with 8 *P. micrantha* sequencing scaffolds, highlighting the major translocation events identified between the 2 species in this investigation.

Following the inparanoid analysis, 33,127 genes returned an orthologous relationship with 1 or more *F. vesca* gene predictions at the amino acid level (98.6%). A subsequent BLAST analysis of the gene predictions against the *F. vesca* v2.0 pseudomolecules identified 24,641 *P. micrantha* genes that returned an unambiguous match with a *F. vesca* orthologue. A total of 1682 *P. micrantha* sequence scaffolds, containing 315,081,089 bp (96.5% of the total sequence) contained at least 1 gene that was anchored to 1 of the *F. vesca* v2.0 pseudomolecules. Of those, 573 contained at least 10 orthologous gene sequences, 118 contained at least 50 orthologous sequences, and 32 contained more than 100 orthologous (Supplementary Excel File 1). Scaffold “Contig145,” the largest scaffold in the *P. micrantha* genome sequence (3,488,351 bp), contained the largest number of orthologous gene sequences anchored to the *F. vesca* v2.0 genome sequence (560), while scaffold “Contig2191” was the smallest anchored scaffold at 1163 bp and containing a single orthologous gene sequence. Comparison of the 2 genomes revealed a remarkable degree of micro-synteny, with the majority of the *P. micrantha* scaffolds spanning uninterrupted regions of the *F. vesca* genome sequence (data not shown). A very high degree of collinearity in gene order was observed between *P. micrantha* scaffolds and the *F. vesca* pseudomolecules (Fig. 2a). In general, only a small number of inversions were observed between syntenic blocks between the 2 genomes, and just 8 *P. micrantha* scaffolds contained distinct syntenic blocks that aligned with more than 1 *Fragaria* pseudomolecule (Fig. 2b). However, scaffold anchoring to a genetic map was not performed for the *P. micrantha* genome sequence, and as such, a comparison of macrosynteny between *Fragaria* and *Potentilla* could not be made.

### Gene expression during fruit development

**Figure 3:**
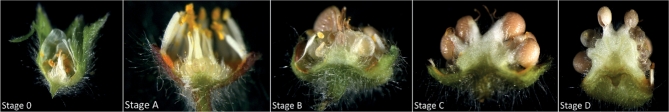
*Potentilla micrantha* flower and fruit developmental stages used for RNA extraction.

**Figure 4:**
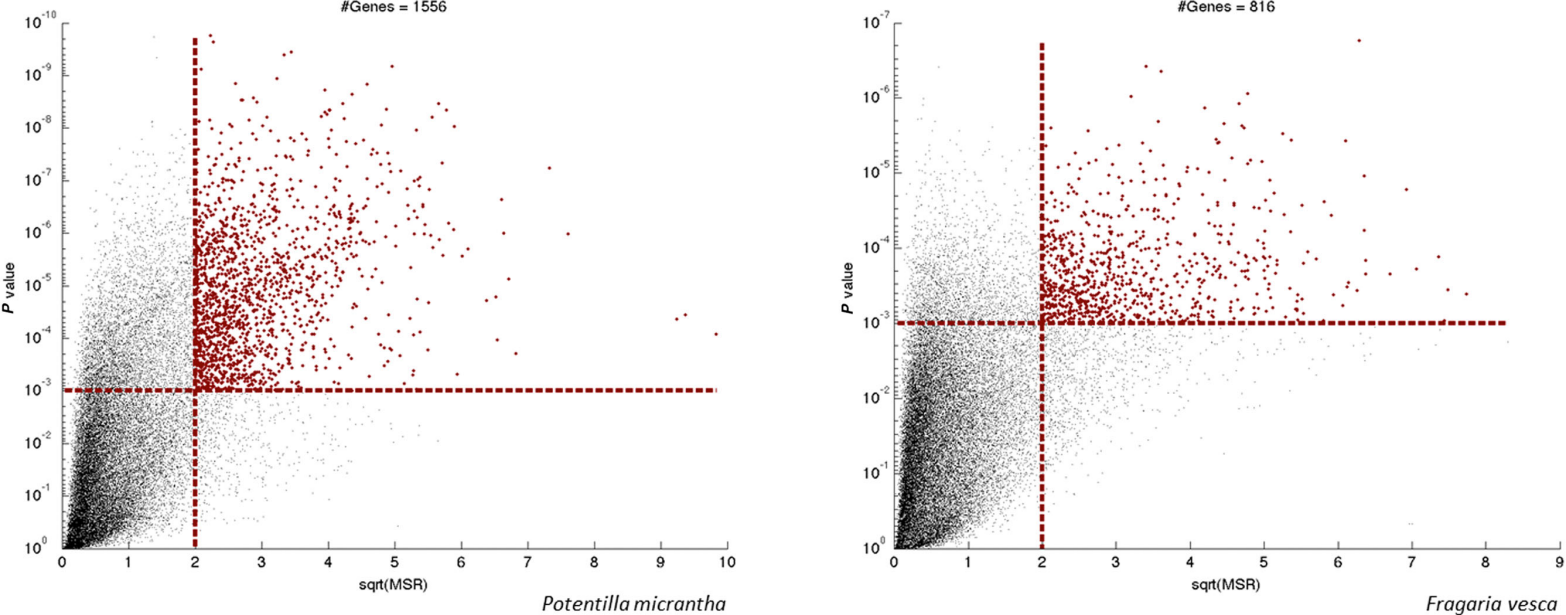
Differentially expressed genes during fruit development in *Potentilla micrantha* and *Fragaria vesca.* Volcano plots of differential expression analysis between the 4 developmental stages A-B-C-D in *P. micrantha* and *F. vesca*. Using a cu-off of sqrt (MSR) >2.00 and *P* value <10^−3^, 1556 genes were differentially expressed in *P. micrantha*, while 816 genes were differentially expressed in *F. vesca*.

**Figure 5:**
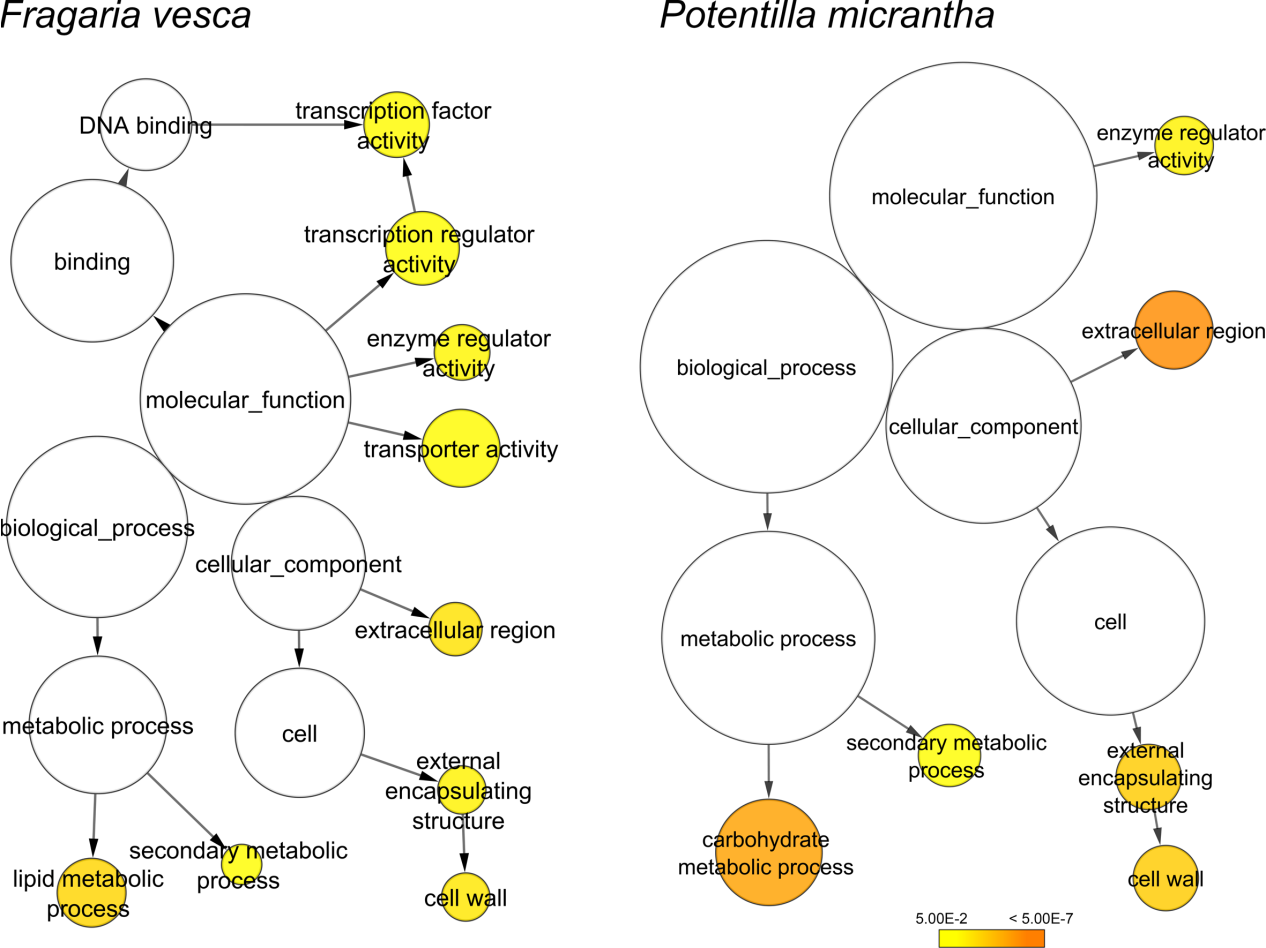
Overrepresented GO-slim categories in *Fragaria vesca* and *Potentilla micrantha* differentially expressed gene sets. The circles are shaded based on significance level (yellow = FDR below 0.05), and the radius of each circle is proportional to the number of genes included in each GO-slim category.

**Figure 6:**
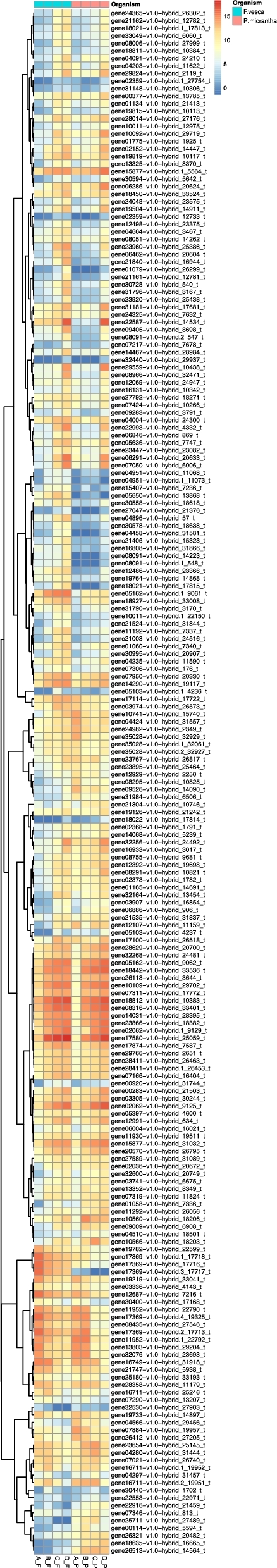
Heat map comparing the log expression values of 205 genes (orthologs of both *Fragaria vesca* and *Potentilla micrantha*). The rows (genes) were sorted using hierarchical clustering using “correlation” distance and “complete” linkage. A–D correspond to the 4 developmental stages defined in the Methods section.

Tissues from 5 stages of flowering and “fruit” development were harvested from *P. micrantha* flowers in biological duplicates or triplicates for RNA isolation. The stages of flowering followed those identified in *Fragaria* by Kang et al. [12], with the addition of a stage 0 (unopened flowers) and young unexpanded leaf tissue. The selected developmental stages are shown in Fig. 3. RNA-seq libraries were made and sequenced with Illumina HiSeq2000. Following Quality control (QC) and adapters trimming, 619,085,115 101 bp paired reads were obtained from the 12 *P. micrantha* RNA-seq libraries. Sequencing yield from individual libraries ranged from 29,653,058 to 60,158,302 reads per sample (Additional File 4, Table S4). Following trimming, the number of reads available for *Fragaria* from the published sequences of Kang et al. [12] were 1,236,882,540, with reads per library ranging from 109,643,225 to 155,643,061. Between 62% and 69% of *P. micrantha* filtered reads per library mapped to the *P. micrantha* gene prediction set, and 63%–67% of *F. vesca* filtered reads per library mapped to the *F. vesca* gene predictions (Additional File 4, Table S4). A total of 1556 genes were differentially expressed between the 4 developmental stages in at least 1 pair-wise comparison of the different stages in *P. micrantha*, while in *F. vesca*, 816 genes were differentially expressed in at least 1 of the contrasts (Fig. 4). A total of 52.44% and 43.38% differentially expressed genes were gene ontology (GO) annotated for *P. micrantha* and *F. vesca*, respectively (Additional File 5, Fig. S1). Analysis of the GO terms for *F. vesca* and *P. micrantha* revealed an enrichment for lipid metabolic processes, transporter activity, and transcription factor activity and for transcription regulator activity in *F. vesca* over *P. micrantha* (Fig. 5). The gene expression profiles between the 4 developmental stages studied in the 2 species showed no clear, consistent patterns between the 2 species overall (Additional File 6, Fig. S2). However, the common differentially expressed genes displayed largely similar expression patterns (Fig. 6), with some exceptions, most notably gene 1369-v1.0-hybrid and its homologue in *P. micrantha* (17 717_t), a predicted 3-hydroxy-3-methylglutaryl coenzyme A reductase 1, which was highly expressed in *F. vesca* but exhibited far lower levels in *P. micrantha*.

### Analysis of MADS-box conserved domain-containing genes in *Potentilla* and *Fragaria*

**Figure 7:**
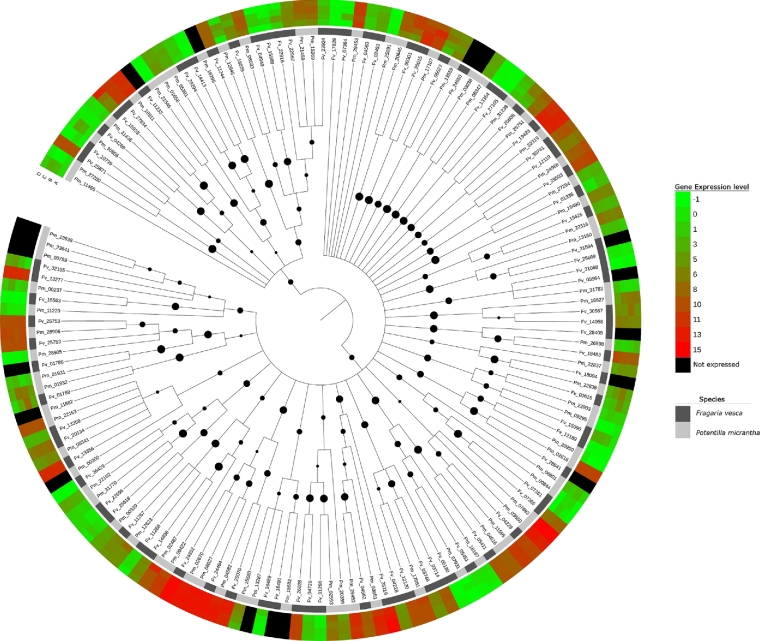
A maximum likelihood-based phylogenetic reconstruction of the *Potentilla micrantha* and *Fragaria vesca* genes containing MADS-box motifs, along with the relative gene expression levels for each gene. Categories A–D refer to the developmental stages defined in the Methods section. Filled circles represent the relative level of support for each relationship defined in the maximum likelihood analysis.

**Figure 8:**
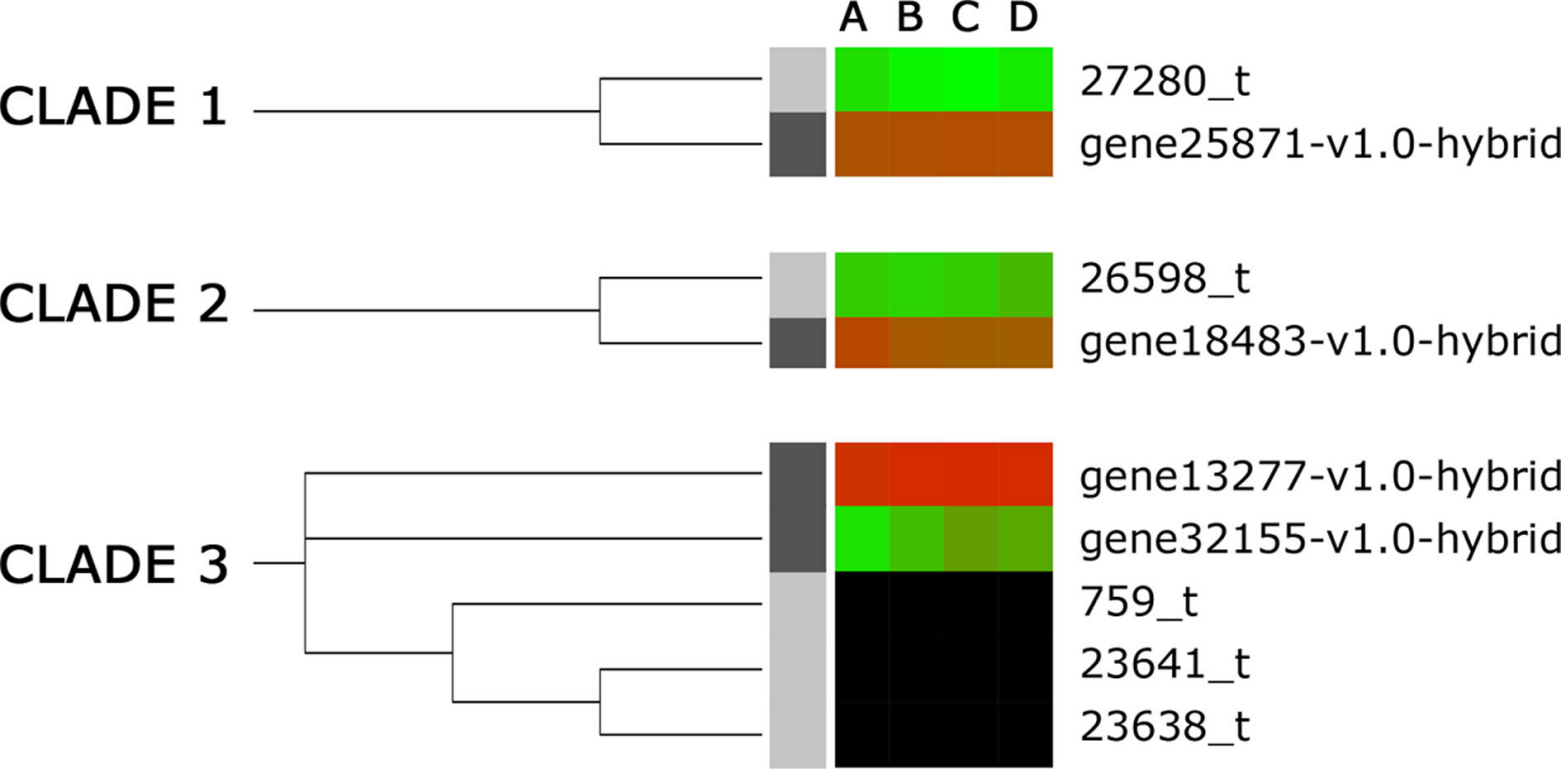
The 3 identified clades of orthologous MADS-box motif containing genes that were not expressed or poorly expressed in *Potentilla micrantha* but highly expressed in *Fragaria vesca.* Categories A–D refer to the 4 developmental stages defined in the Methods section.

A total of 75 *P. micrantha* and 81 *F. vesca* predicted proteins containing MADS-box conserved domains were aligned and phylogenetic trees were obtained to reliably identify orthology relationships between *P. micrantha* and *F. vesca* genes. The 3 methods used for phylogenetic reconstruction (maximum likelihood [ML], maximum parsimony [MP], and neighbor-joining [NJ]) returned largely congruent topologies for the nodes with more than 50% bootstrap support, with NJ providing a slightly more resolved tree given the use of a pairwise, instead of a partial, deletion approach. Figure 7 displays the ML phylogenetic reconstruction of the *P. micrantha* and *F. vesca* genes containing MADS-box, along with the gene expression levels for each gene (data for the NJ and MP trees are not shown). The majority of the genes were retained after the divergence of the species, indicated by a large proportion of orthologous pairs retrieved. Only a few events of lineage-specific gene loss/duplication were observed. Both observations are in line with the lack of ploidy changes within *P. micrantha* and *F. vesca* in the estimated 24.22 million years since species divergence. As expected, the majority of orthologous pairs shared similar expression patterns. Based on the ML gene tree, however, 3 clades of orthologous genes were identified that were not expressed, or were poorly expressed, in *P. micrantha* but highly expressed in *F. vesca* (Fig. 8). The 3 clades, numbered 1, 2, and 3 in Fig. 8, contained the following genes: clade 1 contained genes 27 280_t (*P. micrantha*) and gene25871-v1.0-hybrid (*F. vesca*), which displayed the highest homology to *A. thaliana* AGL36, a sequence-specific DNA binding transcription factor active during endosperm development [13]; clade 2 contained genes 26 598_t (*P. micrantha*) and gene18483-v1.0-hybrid (*F. vesca*), whose closest *A. thaliana* homologue was AGL62, a MADS gene that promotes embryo development, indicating an essential role of endosperm cellularization for viable seed formation [14]; and clade 3 contained *P. micrantha* genes 23 638_t, 23641t and 759_t and *F. vesca* genes gene32155-v1.0-hybrid and gene13277-v1.0-hybrid, whose closest *A. thaliana* homologue AGL15 delays senescence programs in perianth organs and developing fruits and alters the process of seed desiccation [15].

### Analysis of the repetitive component of the *Potentilla micrantha* genome

In total, 1,001,838 of 1,484,780 reads clustered with RepeatExplorer were grouped into 107,190 clusters, representing 67.5% of the genome. No predominant repeat families were identified in the *P. micrantha* genome, with the most redundant repeat cluster representing just 1.18% of the total genome length. Long terminal repeat (LTR) retrotransposons made up the main fraction (24.1%) of the *P. micrantha* genome (Additional File 7, Fig. S3), with a *Gypsy* to *Copia* ratio of approximately 2:1. Terminal-repeat retrotransposons in miniature were poorly represented, making up just 0.2% of the genome, while putative DNA transposons accounted for 5.7% of the genome and included putative CACTA, Harbinger, and hAT elements, with other, unclassified repeats accounting for 10.6% of the genome. A comparison of the repetitive portion of the *F. vesca* and *P. micrantha* genomes performed by pairwise clustering of Illumina sequence reads revealed significant diversification between the repetitive component of the genomes of the 2 species (Additional File 8, Fig. S4). Among the top 291 repeat clusters that had a genome proportion >0.01%, 107 were specific to *P. micrantha*, 51 were specific to *F. vesca*, while only 25 were similarly represented in the 2 species. Among all repeat classes, only ribosomal DNAs show similar genome proportions between *P. micrantha* and *F. vesca*.

### *Potentilla* full-length LTR-Retrotransposable element (RE) characterization, annotation, and insertion age

Of the 505 characterized LTR-Retrotransposable elements (REs), 220 (43.6%) belonged to the *Copia* superfamily, with the greatest proportion belonging to the *Bianca* family, 256 (50.7%) belonged to the *Gypsy* superfamily, with the greatest proportion belonging to the *Ogre/TAT* family, while the remaining 29 (5.7%) could not be placed into a specific superfamily. Table 2 lists the proportion of the annotated 505 LTR-REs in each superfamily and the numbers of elements contained in each subfamily within the *Copia* and *Gypsy* superfamilies. For RE insertion age determination, a mean synonymous substitution rate between *P. micrantha* and *F. vesca* of 0.064 (K_s_) was estimated by comparing 50 orthologous genes, which equated to 52,703 bp of aligned sequences. Using a timescale of 24.22 million years since the separation of *P. micrantha* and *F. vesca* and the estimated K_s_ of 0.064, a synonymous substitution rate of 2.64 × 10^−9^ substitutions per year was calculated. As mutation rates for LTR retrotransposons have been estimated to be approximately 2-fold higher than silent site mutation rates for protein coding genes [16, 17], a substitution rate per year of 5.28 × 10−9 was used in calculations of LTR-RE insertion dates. When the whole set of usable retrotransposons was taken into account, the nucleotide distance (K) between sister LTRs showed a large degree of variation between retro-elements, ranging from 0 to 0.124 using the Kimura 2 parameter method, which represents a time span of at most 23.54 million years.

**Table 2:** Annotation of 505 full-length LTR-retrotransposons of *Potentilla micrantha*.

Superfamily	Family	Number	Percentage
Ty1-*Copia*	*AleI/Retrofit*	14	2.77
	*AleII*	26	5.15
	*Angela*	20	3.96
	*Bianca*	114	22.57
	*Ivana*	23	4.55
	*Maximus/SIRE*	10	1.98
	*TAR/Tork*	11	2.18
	Unknown	2	0.40
	Total	220	43.56
Ty3-*Gypsy*	*Athila*	3	0.59
	*Chromovirus*	42	8.32
	*Ogre/TAT*	186	36.83
	Unknown	25	4.95
	Total	256	50.69
Unclassified		29	5.74

## Materials and Methods

### Plant material, flow cytometry, and DNA isolation

A specimen of *P. micrantha* was collected from Avala, Serbia, in spring 2012 and subsequently used for sequencing. The plant was maintained in a growth room at a constant temperature of 24°C during the day and 18°C at night, with a 16-hour photoperiod to encourage new shoot development. Young leaves were harvested and subjected to flow cytometry by Plant Cytometry Services, NL. Measurements were taken in triplicate against a *Vicia minor* internal standard using the propidium iodide fluorescent dye. The *F. vesca* accession “Hawaii 4” for which a whole genome sequence has been published [51] was analyzed for comparison. Prior to harvesting leaf material for DNA extraction, the plant was moved to a darkened growth chamber for 120 hours, maintaining a constant temperature of 22°C. DNA was extracted from young, unexpanded leaf material using the modified Cetyl trimethylammonium bromide (CTAB) extraction protocol [18], quantified using a Nanodrop spectrophotometer and Qubit fluorometer, and assessed for integrity by agarose gel electrophoresis against a λ *Hind*III size standard.

Since *P. micrantha* does not reproduce asexually from runners, a seedling population obtained from the selfing of the original mother plant was maintained from which to harvest tissue from stages of floral and fruiting development.

### Tissue sampling, RNA extraction, and sequencing

Tissues from 5 stages of flowering and “fruit” development were harvested from untreated flowers in biological duplicates or triplicates for RNA isolation. The stages of flowering followed those identified in *Fragaria* by Kang et al. [12], with the addition of a stage 0 (unopened flowers) and young unexpanded leaf tissue. The selected developmental stages are shown in Fig. 3. RNA was extracted from 50 mg of snap-frozen tissue from each developmental stage using the Spectrum plant total RNA extraction kit (Sigma) with an on-column DNase I digestion (Sigma) step. The extraction protocol followed the manufacturers’ recommendations with 2 minor modifications: 1% Polyvinylpyrrolidone (PVP) was added to the lysis solution and the number of washes at each stage was doubled (ie, 2 washes were performed with wash solution 1 and 4 washes were performed with wash solution 2). The RNA extracted from each sample was diluted in 50 μL of elution solution (Sigma). Following elution, total RNA was quantified using a Nanodrop spectrophotometer and Qubit fluorometer and assessed for integrity using a Bioanalyzer (Agilent). Samples returning a RNA integrity number (RIN) value greater than 7.5 were considered acceptable for sequencing. A total of 12 Illumina TruSeq libraries were constructed from 2 μg of total RNA. Libraries were made from the following samples: 1 from stage 0, 2 from stage 1, 2 from stage 2, 3 from stage 3, and 3 from stage 4. A final library was made from RNA of young leaf tissue. The libraries were sequenced in triplex per single lane of Illumina HiSeq2000. Samples were indexed and multiplexed, then 101 bp paired-end sequencing was performed using the Illumina HiSeq 2000 platform at the Weill Medical core genomics facility at Cornell University.

### Whole genome shotgun sequencing and assembly

A strategy following the ALLPATHs-LG protocol was followed to produce an initial assembly using second-generation sequence data. Five sequencing libraries were developed: an OLF with an insert size of 170 bp and 4 libraries of 3 kb, 5 kb, 8 kb, and 12 kb. The OLF library was created using the Illumina Nextera library preparation kit following the manufacturer’s recommendations and was sequenced in simplex on a single lane of Illumina HiSeq2000, while the MP libraries were prepared using the Illumina Mate Pair Library v2 kit following the manufacturer’s recommendations and were subsequently sequenced in duplex. All sequencing was performed at the Weill Medical Center core genomics facility at Cornell University. ALLPATHS-LG (ALLPATHS-LG, RRID:SCR_010742) [19] was run using the sequencing libraries described above using default settings. Subsequently, a selection of SMRT-bell sequencing libraries were constructed using various versions of the PacBio RS sequencing kits and chemistries (Additional File 2, Table S2), and PBJelly (PBJelly, RRID:SCR_012091) [10] running default settings was used to incorporate data generated using the PacBio RS platform (Pacific Biosciences) into the ALLPATHS-LG Illumina assembly scaffolds. Identification of benchmarking universal single-copy orthologs was performed using BUSCO v3 (BUSCO, RRID:SCR_015008) [11] running default parameters and using 1440 BUSCO groups from the embryophyta_odb9 (plant) lineage data.

### Gene prediction, annotation, determination of gene orthology, and evaluation of synteny between *Potentilla* and *Fragaria* genomes

First, *ab initio* repeat finding was done with RepeatModeler (RepeatModeler, RRID:SCR_015027) [20] that was run on the complete set of genomic scaffolds set and a repeat library was created. Next, the genome was masked using RepeatMasker (RepeatMasker, RRID:SCR_012954) [21]. Gene prediction was done with GeneMark-ET [22]. The following parameters were used: a minimum scaffold length of 10 kb, a maximum scaffold gap size of 40 kb, a minimum intron size of 50 bp, a maximum intron length of 10 kb, and a maximum intergenic length of 50 kb. RNA-seq reads from the 12 libraries were aligned to the genome sequence scaffolds using the STAR tool with default parameters [23]. Reads from the 12 RNA-seq datasets were aligned to the genome. Mapping of RNA-seq reads that included intron junctions led to the identification of introns. Introns with a high “intron score” (identified by more than 60 RNA-seq reads) were considered to be reliably identified. Predicted genes were annotated using BLAST2GO (BLAST2GO, RRID:SCR_005828) [24]. The nonredundant National Centre for Biotechnology Information (NCBI) protein database was downloaded, and BLAST was run locally. Results from the BLAST analysis were uploaded to the BLAST2GO server, and GO analyses were performed using default parameters.

Orthologous relationships between *Fragaria* and *Potentilla* genes were determined through sequence clustering performed using Inparanoid 7 [25]. Analyses were based only on homology, as an alternative to the more stringent ortholog classification. *Prunus persica* v2.0.a1 predicted proteins downloaded from the Genome Database for Rosaceae (GDR) [26]; *P. micrantha* and *F. vesca* protein sequences were blasted all against all; and the output file was filtered at the following thresholds: maximum E-value = 10^−4^ and query coverage of at least 50%. The resulting file was used as an input to the Markov cluster algorithm (MCL) using as edge weight −log_10_(evalue) (all E-values = 0 were changed to 1E-300). To explore more thoroughly the homology network used as input, the MCL algorithm was run at different granularity levels (inflation parameter equal to 1.5, 1.7, 2.0, 2.3, 2.4, 2.7, 3), and then a table indicating cluster memberships at the different stringencies was compiled for each node. Ortholog classification was produced using Inparanoid 7 [25] for pairs of species in all combinations. The resulting sqltables were then used as an input for QuickParanoid [27], and the sequences were combined in a 3-species ortholog classification. The clusters obtained with QuickParanoid were used to calculate the number of genes contained in each cluster for both *Potentilla* and *Fragaria*.

*Potentilla* gene predictions for which an orthologous relationship was identified through the inparanoid analysis were used as queries to identify the physical locations of orthologous sequences on the *F. vesca* v2.0 pseudomolecules. Those sequences that returned a single, unambiguous match on the genome sequence were used to evaluate synteny between the 2 species. Since the *Potentilla* genomic scaffolds were not oriented and ordered against a reference genetic map, conservation of synteny between the *Potentilla* and *Fragaria* genomes was determined through a comparison of the physical positions of orthologous gene sequences on the sequence scaffolds of *Potentilla* and the pseudomolecules of *Fragaria.* Criteria for the identification of syntenic regions followed that of Jung et al. [5]. No attempt was therefore made to infer macro-syntenic structure on a chromosome scale between the 2 genomes.

### Gene expression during stages of fruit development in *Potentilla micrantha* and *Fragaria vesca*

The quality of the raw reads generated as described above was checked with FastQC (FastQC, RRID:SCR_014583) [28]; Trimmomatic (Trimmomatic, RRID:SCR_011848) [29] was used to remove adapter sequences. The *F. vesca* .sra files [12] were used to compare gene expression in *Fragaria* with *Potentilla*. *Fragaria* reads from the same developmental stage were merged and treated as a single dataset since data from *Potentilla* was not generated from individual floral organs. The 12 trimmed *P. micrantha* RNA-seq libraries were mapped on the *P. micrantha* gene prediction CDS, while the 10 *F. vesca* sets were mapped to the *F. vesca* v1.0 gene prediction CDS [4] downloaded from the GDR [26] using Bowtie2 [30] and default settings. The number of reads mapping to each gene for each RNA set was calculated from the .sam alignment files derived from Bowtie2.

Counts of RNA-seq reads over transcripts were used to calculate the gene expression level in Fragments per kilobase of transcripts per million mapped reads (FPKM) = 10^9^*ER/(EL × MR), where ER was the number of mapped reads in the exons of a particular gene, EL was the sum of exon length in base pairs, and MR was the total number of mapped reads [31]. FPKM was used to distinguish expressed genes from inactive genes (those not returning any expression data) during the flower development in each species. Further, FPKM was used to define a set of highly expressed genes. Genes were considered as “highly-expressed” if FPKM >1000. Genes that returned an FPKM <1000 in all samples were removed from further differential expression analysis. The retained differentially expressed genes were processed by performing a linear rescaling of the log_2_ counts, aligning the distributions for every sample at their distribution modes, followed by variance stabilization to ensure homoscedasticity. A 1-way analysis of variance (ANOVA) was performed gene-by-gene on the rescaled log_2_ counts to detect changes in expression among different developmental phases. Differentially expressed genes (DEGs) were selected by setting cutoffs both on the *P* values from the ANOVA F tests, as well as on the magnitude of observed changes represented by the square root of the ANOVA Mean-squared-anomaly (MSR) values (equivalent to using volcano plots for 2-condition studies). Genes were considered differentially expressed if the sqrt (MSR) >2.00 and *P* value <10^−3^.

GO enrichment analysis of DEG sets of *P. micrantha* and *F. vesca* was carried out using BLAST2GO 2.8.0 [24] with “Fisher exact test” method, considering as “enriched” the GO categories with FDR <0.05. *Potentilla micrantha* whole transcriptome functional annotation obtained in this work was used as background for *Potentilla* GO enrichment analysis, while the “InterPro GO for GeneMark hybrid transcripts” database downloaded from the GDR website was used as background for *F. vesca*. Cytoscape 3.5.1 (Cytoscape, RRID:SCR_003032) [32] with the BiNGO 3.0.3 plugin was used for the GO-slim network visualization of enriched GO categories over *F. vesca* and *P. micrantha* DEGs. For determination of overrepresentation, the Benjamini and Hochberg FDR-adjusted significance level cutoff was 0.05.

### Phylogenetic and functional analysis of MADS-box domain-containing genes and gene expression profile mapping

Protein sequences of *Potentilla* (this publication) and *Fragaria* (Fvesca_v1.0_hybrid; [26]) were analyzed on the NCBI conserved domain database [33]. All proteins containing a MADS-box domain were retrieved and the MADS-box extracted with Bedtools getfasta [34] using default parameters. An initial sequence alignment was carried out using ClustalW, and pairwise distances were calculated to eliminate outliers. A total of 16 sequences were removed from further analysis since they were too short and possessed incomplete N-terminal ends, indicating they were likely pseudogenes. The alignment used for phylogenetic analysis was constructed with SATé-II [35] and contained 156 protein sequences (75 from *Potentilla* and 81 from *Fragaria*).

Three methods, ML, MP, and NJ, each with 1000 bootstrap replicates, were used for phylogenetic reconstruction of the MADS-box domain containing genes using Mega 7.0.14 [36]. Where data were missing in the alignment, deletion of columns containing a fraction of missing data above 10% and 30% was performed for ML and MP methods. Pairwise deletion was instead used in the case of NJ to maximize the phylogenetic information retained in the alignment. The ML topology was used as reference for further analysis.

The expression profiles of the genes containing a MADS-box were used to decorate the phylogenetic tree using iTOL v2 [37], allowing the identification of orthologous MADS-box gene pairs displaying differential gene expression profiles between *Potentilla* and *Fragaria*. Curated annotation of differentially expressed putative gene function was carried out using BLASTp homology searches of the TAIR (The Arabidopsis Information Resource) database [38].

### Analysis of the repetitive component of *Potentilla* genome

To identify and characterize genomic repeats in the *P. micrantha* genome, a reduced set of 2,000,000 randomly selected genomic Illumina reads, corresponding to 0.57 × of the *P. micrantha* genome, were subjected to clustering using RepeatExplorer [39]. Among the clusters produced, the top clusters, with a genome proportion higher than 0.01%, were annotated using 0.2 as the cutoff for cluster connection through mates. Clusters that were annotated as similar to phi-X174 were removed as contaminants. The output of RepeatExplorer was also used to prepare an in-house library containing all contigs belonging to clusters annotated by RepeatExplorer as LTR-REs by similarity search against RepBase [40]. Subsequently, pairwise hybrid clustering between a random set of 1,431,114 Illumina reads derived from *P. micrantha* genomic DNA and 1,090,102 *F. vesca* genomic reads, each corresponding to 0.41× of the respective genomes, was performed using RepeatExplorer [39].

### *Potentilla* full-length LTR-RE characterization

LTR-FINDER [41] was used to isolate putative full-length LTR-REs from 280 randomly selected *Potentilla* genome sequence scaffolds, and alignment boundaries were obtained by adjusting the ends of LTR-pair candidates using the Smith–Waterman algorithm. These boundaries were readjusted based on the occurrence of the following typical LTR-RE features: (a) the putative LTR-RE were flanked by the dinucleotides TG and CA at 5΄ and 3΄ ends, respectively; (b) a target-site duplication (TSD) 4–6 nt in length was present in the sequence; (c) a putative 15–18 nt primer binding site complementary to a tRNA at the end of the putative 5΄-LTR was present in the sequence; and (d) a 20–25-nt polypurine tract just upstream of the 5΄ end of the 3΄ LTR was present in the sequence. Putative LTR-REs were manually validated using DOTTER [42], verifying the occurrence of LTRs, dinucleotides TG and CA at the 5΄ and 3΄ ends, respectively, and TSDs. The validated LTR-REs were annotated using BLASTX and BLASTN querying the NCBI nr nucleotide and protein NCBI databases and RepBase [40]. To limit false-positive detection, a fixed E-value threshold of E <10^−5^ for BLASTN and E <10^−10^ for BLASTX was used. The full-length elements identified were analyzed using RepeatExplorer [39], performing searches for GAG, protease, retrotranscriptase, RNAseH, integrase, and chromodomain derived from plant protein domains from RepBase. The similarity search was filtered at E-value <10^−10^, allowing for both mismatches and frameshifts. The same tool was used to assign full-length elements to specific *Gypsy* or *Copia* lineages. Full-length LTR-REs that were identified as belonging to *Gypsy* or *Copia* superfamilies and clusters annotated as LTR-retrotransposons by RepeatExplorer (see above) were then used as reference datasets for further searches in order to identify previously unclassified elements using RepeatMasker, running default parameters, but with -div set to 20.

For determination of RE redundancy, approximately 32,000,000 raw *Potentilla* Illumina paired-end reads were randomly selected, corresponding to 10.3× genome coverage. After removal of organellar contamination performed by mapping the reads to an in-house Rosaceae organellar database and the removal of duplicate reads, 25,206,510 reads corresponding to 7.2× equivalent genomic coverage were used for redundancy analysis by mapping the reads to all REs characterized in the *Potentilla* genome using CLC-BIO Genomic Workbench 8.0 (CLC-BIO, Aarhus, Denmark). Mismatch cost, deletion cost, and insertion cost were fixed at 1, and similarity and length fraction were both fixed at 0.9, 0.8, 0.5, or 0.4 to obtain high, medium, low, or very low stringencies, respectively. As reads that mapped to multiple distinct sequences were few and distributed randomly throughout the dataset, the number of reads mapping to each RE was taken as the degree of redundancy of that sequence within the genome. The effective abundance of a particular class of reads was calculated as the proportion of the total number of reads mapped in each class, with respect to the overall number of genomic reads mapped, using optimal stringency parameters, ie, where further relaxation of stringency did not significantly increase the number of mapped reads.

The abundance of each single RE sequence in the genome was analyzed by mapping *Potentilla* DNA reads, corresponding to 2× genome coverage to the full-length REs characterized, 1 by 1, using BWA (alignment via Burrows–Wheeler transformation) version 0.7.5a-r405 (BWA, RRID:SCR_010910) [43] running the following parameters: bwaaln -t 4 -l 12 -n 4 -k 2 -o 3 -e 3 -M 2 -O 6 -E 3. The resulting single-end mappings were resolved via the samse module of BWA, and the output was converted to .bam file format using SAMtools version 0.1.19 [44]. Subsequently, SAMtools was used to calculate the number of mapped reads for each alignment using the following parameters: samtools view -c -F 4.

### Determination of RE insertion age

Retrotransposon insertion age was estimated through a sequence divergence comparison of the 5΄- and 3΄-LTRs of each putative full-length retrotransposon. Synonymous substitution rates were calculated for 50 pairs of orthologous genes of *P. micrantha* and *F. vesca*, using a time of divergence of 24.22 million years [3]. Subsequently, the 2 LTRs were aligned with ClustalX software [43], indels were eliminated, and the number of nucleotide substitutions was counted using DnaSP [44] for each retrotransposon. The insertion times of retrotransposons with both LTRs were dated using the Kimura 2 parameter method [45], calculated using DnaSP, and a synonymous substitution rate that is 2-fold that calculated for genes [16, 17].

## Discussion

### Data validation and quality control

In this investigation, the genome of *P. micrantha*, a member of the Rosaceae family, a diverse family of fruiting perennial plant genera, was sequenced using both short-read Illumina and long-read PacBio sequence data, and the resulting data were assembled into a highly contiguous reference sequence for the genus *Potentilla*. PacBio data (using early iterations of the sequencing chemistry) were proficiently integrated with short-reads, significantly improving the contiguity of the assembly. The genome assembly presented here has a quality similar to the *F. vesca* genome, containing significantly fewer unsequenced gaps within scaffolds, and is far more contiguous than that of *Rubus occidentalis* [46]. Along with the set of gene predictions presented, it represents a valuable resource for studying the genetic basis of a number of key morphological traits that differ between *P. micrantha* and its closest sequenced relatives.

*Potentilla* and *Fragaria* are separated by just 24.22 million years of evolution [4]. However, in this investigation, we show the genome of *P. micrantha* is 59.6% larger than that of *F. vesca* and it is also larger than the available genomes of the other Fragariianae, ie, *Rubus* [47, 48] and *Rosa* species [49, 50] to which it is more distantly related. We also demonstrate here that *P. micrantha* and *F. vesca* exhibit a remarkable degree of microsynteny of the coding portion of the genome, with the main differences being short-range inversions. Nonetheless, the apparent differences in insertion age of transposable elements in the 2 genomes have led to significant differences in the repetitive portions. Whereas the genome structure of *P. micrantha* is similar to that of most angiosperm species [51], with a repetitive component amounting to around 41.5% of the total genome content, the genome of *F. vesca* has been previously demonstrated to contain just 22% repetitive elements [4]. Contrary to the coding or nonrepetitive genome, the repetitive fractions of the *P. micrantha* and *F. vesca* genomes are highly diversified, suggesting that the overwhelming majority of retrotransposon activity in the genus *Potentilla* occurred after the divergence of the 2 genera from their common progenitor. The data presented here strongly indicate that retrotransposon activity (or the lack thereof in the genus *Fragaria*) is responsible for the significant difference between the genome size of *Fragaria* and its closest relatives and support the assertion of Potter et al. [2] that *Fragaria* should be treated as a distinct genus, separate from *Potentilla*.

Gene expression patterns for differentially expressed genes that were common to both *F. vesca* and *P. micrantha* were largely similar between the 2 species; however 1 gene, a 3-hydroxy-3-methylglutaryl coenzyme A reductase 1 homologue, displayed significantly higher gene expression levels in *F. vesca.* The 3-hydroxy-3-methylglutaryl coenzyme A reductase 1 gene catalyzes the first committed step in the cytosolic isoprenoid biosynthesis pathway [52]. Loss-of-function mutants of this gene in *Arabidopsis* display a dwarf phenotype due to suppression of cell elongation and reduced sterol levels [52]. Sterols are precursors in cellulose synthesis, important for cell wall formation [53] and fruit development. As such, upregulation in the 3-hydroxy-3-methylglutaryl coenzyme A reductase 1 gene during fruit development in *F. vesca* over *P. micrantha* may indicate a role for this enzyme in berry formation in *Fragaria*.

In contrast to the gene expression patterns of differentially expressed genes common to both *F. vesca* and *P. micrantha* during fruit development, global patterns of gene expression during fruit development differed between the 2 species. The GO for the *F. vesca* expression profile was enriched for genes with transcription factor and transcription regulator activity as well as transporter activity and lipid metabolic processes. A study of the differences in transcriptional regulation between *F. vesca* and *P. micrantha* therefore may provide clues to the genetic basis of berry formation in *F. vesca*. MADS-box transcription factors have been implicated in a wide and extremely diverse array of developmental processes in plants [54] and were initially demonstrated to play a major role in floral organ differentiation, including gametophyte, embryo, and seed development, as well as flower and fruit development. A study of the differential expression of MADS-box genes revealed 3 clades of orthologous genes where gene expression of orthologous genes was upregulated in *F. vesca* with respect to *P. micrantha*, where the genes were either shown to have lower expression levels or were not expressed in the tissues studied. One clade contained genes that were homologous to AGL36, a transcription factor crucial for endosperm differentiation and development [13, 55]. Another clade contained genes homologous to *A. thaliana* AGL62, which likewise has been implicated in embryo development and is thought to have an essential role in endosperm cellularization for viable seed formation [14]. The third clade contained genes homologous to AGL15 reported to have diverse roles in embryogenesis, fruit maturation, seed desiccation, and the repression of floral transition [15, 56], as well as being a positive regulator of the expression of mir156, a repressor of floral transition [57].

### Reuse potential

The set of genomics tools developed here for *P. micrantha*, a nonfruiting relative of *F. vesca* includes a genome sequence, gene predictions, and RNA-Seq data. It is a valuable resource and will form the foundation for future genomics studies in the species and comparative genomics studies within the Rosoideae subfamily of Rosaceae in particular. It will also allow more detailed future functional studies of fleshy receptacle (berry) development.

## Availability of supporting data 

The dataset supporting the results of this article are available in the GenBank repository (project PRJEB18433P). The genome reference sequence and gene predictions can be downloaded from the GigaScience GigaDB repository [58].

## Additional files

Additional File 1: Table S1. Illumina sequencing libraries used in the sequencing of the *Potentilla micrantha* genome including fragment sizes and total genome depth of coverage.

Additional File 2: Table S2. PacBio RS sequencing kits and chemistries used for *Potentilla micrantha* sequencing.

Additional File 3: Table S3. RNA-seq read data used for gene prediction and number of splice sites identified in the *Potentilla micrantha* genome.

Additional File 4: Table S4. *Potentilla micrantha* and *Fragaria vesca* RNA-seq reads statistics.

Additional File 5: Fig. S1. Distribution of predicted genes *Potentilla micrantha* and *Fragaria vesca* mapped, blasted, and GO-annotated by Basic Local Alignment Search Tool 2GO analysis.

Additional File 6: Fig. S2. The differential gene expression profiles between the 4 developmental stages of fruit development studied in *Fragaria vesca* and *Potentilla micrantha*.

Additional File 7: Fig. S3. The overall abundance of different classes of transposons within the *Potentilla micrantha* genome according to the analyses performed using RepeatExplorer.

Additional File 8: Fig. S4. Genome proportion in *Potentilla micrantha* and *Fragaria vesca* of 291 repeats clustered using RepeatExplorer. Other repeats include satellite DNAs, pararetroviruses, and 1 LINE.

## Funding

This work was funded by a grant to the Fondazione Edmund Mach (F.E.M.) from the Autonomous Province of Trento grants office. A.C. acknowledges funding from the Department of Agriculture, Food, and Environment of Pisa University, project “Plantomics.”

## Abbreviations

ANOVA: analysis of variance; BLAST: basic local alignment search tool; BUSCO: bench-marking universal single-copy orthologous; CDS: coding DNA sequence; CTAB: cetyl trimethylammonium bromide; DEG: differentially expressed gene; FDR: Benjamini Hochberg correction; FPKM: fragments per kilobase of transcript per million mapped reads; GA3: gibberellic acid; GDR: genome database for rosaceae; GO: gene ontology; LTR: long terminal repeat; MCL: markov cluster; ML: maximum likelihood; MP: maximum parsimony; MSR: mean squared anomaly; NAA: naphthaleneacetic acid; NCBI: national center for biotechnology information; NJ: neighbor-joining; NPA: N-1-naphthylphthalamic acid; OLF: overlapping fragment library; PVP: polyvinylpyrrolidone; RE: retrotransposable element; RIN: RNA integrity number; SNP: single nucleotide polymorphism; SMRT: single molecule real-time; TSD: target-site duplication QC: quality control.

## Conflict of interests

All authors report no competing interests.

## Author contributions

M.Buti performed the experiments, analyzed and interpreted all data, and authored the manuscript. M.M., P.S., and A.C. analyzed sequence data and performed genome assemblies. K.E. and M. Brilli assisted with experimental design, analyzed and interpreted gene expression data, and commented on and contributed to the manuscript. L.N. and A.C. performed full-length retrotransposon isolation. E.B., F.M., and A.C. performed clustering, annotation, and redundancy analyses of repetitive sequences. E.B., F.M., L.N., and A.C. participated in the interpretation and discussion of results and contributed to the writing of the manuscript. A.L and M. Borodovsky performed gene predictions and analyzed and interpreted the data. L.G. and N.Š. assisted with experiments, interpreted data, and contributed to the manuscript. M.A. and J.W. assisted with genome assemblies and gene annotation. C.V. analyzed and interpreted phylogenetic data and contributed to the manuscript. R.V. commented on the manuscript. D.J.S. designed the study, assisted with the experiments, analyzed and interpreted the data, and authored the paper.

## Supplementary Material

GIGA-D-17-00155_Original_Submission.pdf

GIGA-D-17-00155_Revision_1.pdf

GIGA-D-17-00155_Revision_2.pdf

GIGA-D-17-00155_Revision_3.pdf

Response_to_Reviewer_Comments_Original_Submission.pdf

Response_to_Reviewer_Comments_Revision_1.pdf

Response_to_Reviewer_Comments_Revision_2.pdf

Reviewer_1_Report_(Original_Submission) -- Manuel Spannagl 01 Aug 2017 Reviewed

Reviewer_1_Report_(Revision_1) -- Manuel Spannagl 21 Nov 2017 Reviewed

Reviewer_2_Report_(Original_Submission) -- Soichiro Nagano 05 Aug 2017 Reviewed

Reviewer_3_Report_(Original_Submission) -- Sook Jung 15 Aug 2017 Reviewed

Reviewer_3_Report_(Revision_1) -- Sook Jung 01 Dec 2017 Reviewed

Additional Files
