# Erratum to: The genome sequence and transcriptome of *Potentilla micrantha* and their comparison to *Fragaria vesca* (the woodland strawberry)

**DOI:** 10.1093/gigascience/giy088

**Published:** 2018-11-16

In the final publication of “The genome sequence and transcriptome of *Potentilla micrantha* and their comparison to *Fragaria vesca* (the woodland strawberry),” by Matteo Buti et al., the GenBank repository project number was incorrect. Reference 9 and 10 were also incorrect. The GenBank repository project number and the references have been corrected. The corrected article is available in *GigaScience*, Volume 7, Issue 4.

The Publisher regrets these errors.

