# Supplementary material for: The genome sequence and transcriptome of Potentilla micrantha and their comparison to Fragaria vesca (the woodland strawberry)
Source: Gigascience. 2017 Feb 15;7(4):giy010. doi: 10.1093/gigascience/giy010 (PMC5893959; doi:10.1093/gigascience/giy010)
Supplement: GIGA-D-17-00155_Revision_2.pdf [file giy010_giga-d-17-00155_revision_2.pdf]

GigaScience

The genome sequence and transcriptome of Potentilla micrantha and their comparison to Fragaria vesca (the woodland strawberry)

--Manuscript Draft--

**Manuscript Number:**	GIGA-D-17-00155R2
**Full Title:**	The genome sequence and transcriptome of Potentilla micrantha and their comparison to Fragaria vesca (the woodland strawberry)
**Article Type:**	Data Note
**Funding Information:**	
**Abstract:**	The genus Potentilla is closely related to that of Fragaria, the economically important strawberry genus. Potentilla micrantha is a species that does not develop berries, but shares numerous morphological and ecological characteristics with F. vesca. These similarities make P. micrantha an attractive choice for comparative genomics studies with F. vesca. In this study, the Potentilla micrantha genome was sequenced and annotated, and RNA-Seq data from the different developmental stages of flowering and fruiting were used to develop a set of gene predictions. A 327 Mbp sequence and annotation of the genome of P. micrantha, spanning 2,674 sequence contigs, with an N50 size of 335,712, estimated to cover 80% of the total genome size of the species was developed. The genus Potentilla has a characteristically larger genome size than Fragaria, but the recovered sequence scaffolds were remarkably collinear at the micro-syntenic level with the genome of F. vesca, its closest sequenced relative. A total of 33,602 genes were predicted, and 95.1% of BUSCO genes were complete within the presented sequence. Thus, we argue that the majority, of the gene-rich regions of the genome have been sequenced. Comparisons of RNA-Seq data from the stages of floral and fruit development revealed genes differentially expressed between P. micrantha and F. vesca. The data presented are a valuable resource for future studies of berry development in Fragaria and the Rosaceae and they also shed light on the evolution of genome size and organization in this family.
**Corresponding Author:**	Daniel James Sargent, PhD<br><br>UNITED KINGDOM
**Corresponding Author Secondary Information:**	
**Corresponding Author's Institution:**	
**Corresponding Author's Secondary Institution:**	
**First Author:**	Matteo Buti, PhD
**First Author Secondary Information:**	
**Order of Authors:**	Matteo Buti, PhD
	Marco Moretto, PhD
	Elena Barghini, PhD
	Flavia Mascagni, PhD
	Lucia Natali, PhD
	Matteo Brilli, PhD
	Alexandre Lomsadze, PhD
	Paolo Sonego, PhD
	Lara Giongo, PhD
	Michael Alonge, MSc

Powered by Editorial Manager® and ProduXion Manager® from Aries Systems Corporation

	Riccardo Velasco, PhD
	Claudio Varotto, PhD
	Nada Surbanovski, PhD
	Mark Borodovsky, PhD
	Judson A Ward, PhD
	Kristoff Engelen, PhD
	Alessandro Cestaro, PhD
	Andrea Cavallini, PhD
	Daniel James Sargent, PhD
**Order of Authors Secondary Information:**	
**Response to Reviewers:**	Dr Daniel James Sargent<br>Driscoll's Genetics Limited<br>East Malling Enterprise Centre<br>New Road<br>East Malling<br>Kent, ME19 6BJ, UK<br>4th January 2018<br><br>Dear Dr Zauner,<br><br>Please find enclosed our revised manuscript: GIGA-D-17-00155R2 The genome sequence and transcriptome of Potentilla micrantha and their comparison to Fragaria vesca (the woodland strawberry), which has been reformatted in the style of a Data Note.<br><br>Below we provide a point-by-point description of the changes made in response to the reviewers comments and hope that the changes we have made will be sufficient for the paper to be acceptable for publication.<br><br>We look forward to hearing from you in due course regarding our revised paper.<br><br>Very best regards,<br><br>Dan Sargent (on behalf of all authors).<br><br>Responses to Reviewer's comments:<br><br>Q1. Overall, I am pleased that the reviewers agree that the latest, revised version of your manuscript presents useful and valuable data. However, I also agree with reviewer 1 that, in the absence of more in-depth analyses, the main value of the paper is now indeed the presentation of a high-quality resource, rather than new biological insights.<br><br>R1. Many thanks for the useful comments. We agree with your feeling that the paper describes more of a technical advance than a description of new biological insights at this stage, but hope we have provided a valuable dataset that can be used for future biological investigations.<br><br>Q2. I therefore feel that your revised manuscript may be more suitable for the format of a "Data Note". Data notes are indexed in the same way as research articles, but the emphasis is on presenting an exceptional resource, together with description and validation of the dataset. If you agree that we consider your next revised version as a data note, please adjust the format (see: https://academic.oup.com/gigascience/pages/data_note). I feel that this would require only minor adjustments to the text itself (e.g. the discussion part could be revised to demonstrate validation and re-use cases of the data). You can keep the Background section for the Data Note format (this is not quite clear from our Instructions).

Please let me know if you agree to this suggestion, and I'm happy to answer any questions regarding the Data Note format.

R2. We are happy for our paper to be published as a data note and have revised the manuscript accordingly (following the guidelines and taking recently published data note articles as examples).

Reviewer reports:
Q3. Reviewer #1: The authors addressed some of the main issues appropriately (synteny, figures etc.). However, for other issues like the transposon section, the hormonal treatment analysis and the miR1511 analysis the main action was just to shorten or completely drop the part. This is Ok and/or was suggested but on the other hand, no real efforts were made to strengthen the comparison/fleshy fruit analyses (or any other analytical part) and most of my suggestions/questions for this and also the annotation and gene expression part were simply ignored. As a result, this study as it stands now mostly provides an "extended description" of the resources generated (although potentially valuable) with clear shortcomings in the analysis and interpretation of the data. Along that lines, I appreciate that the authors in the new version resign from claiming analytical results not there or possible.

R3. We appreciate the reviewer's useful and constructive criticisms on the previous version of our paper and whilst we tried to incorporate as many of the suggested changes as was possible, the lead authors did not have the resources available to make all the suggested improvements, and as such we opted to remove the unsupported claims and refocus the paper as a technical report.

Additional comments:
Q4. a.) Your BUSCO analysis shows that you are missing ~6% of the BUSCO genes from the genome sequence (present) to the final gene prediction (absent). Are they completely missing in the gene prediction or fragmented etc.?

R4. We have provided more detail in the manuscript, detailing the complete and fragmented BUSCO sequences retrieved. We have also performed a BUSCO analysis on the F. vesca gene predictions and provided a comparison of the two to demonstrate that the P. micrantha set is virtually as complete as the F. vesca set.

Q5. b.) I still wonder about the ~9,000 gene predictions not showing a hit on the F. vesca pseudomolecules…do those genes have functional annotation and expression support?

R5. Our sincere apologies for not making this section clearer (it was not explained carefully enough what analyses we are performing and the results were not reported in detail). The Inparanoid analysis attempted to identify orthologous genes between the P. micrantha and F. vesca datasets using protein sequences. This analysis revealed 33,127 P. micrantha genes that had a putative match in the F. vesca gene set (98.6%). However, for the analysis of synteny, we chose only those genes where there was a clear and unambiguous orthologous relationship between the two genomes. This is the reason only 24,555 genes were considered for the synteny analysis. We have amended the text to clarify these points.

Q6. c.) I cannot make much sense out of figure 2B. In the figure resolution I have, individual lines look like they correspond to a single (or very few) gene(s), although I suspect it has to be more genes. It would be good to define the sizes of the blocks somewhere.

R6. We have added the number of genes in each conserved region which are split between pseudomolecules on the F. vesca genome. We have also made it clearer that these are only scaffolds that are split between pseudomolecules (i.e. evidence of major rearrangements). There were many instances of individual genes that did not fall into syntenic blocks.

Q7. d.) I'd move figure 9 to the supplementary material and drop sup Figure S1. It still has the same problem as when it was a main figure.

Powered by Editorial Manager® and ProduXion Manager® from Aries Systems Corporation

	R7. Done as suggested<br><br>Reviewer #3: The authors have addressed all my comments and concerns.<br>Q8. I have one minor comment -I found 'CDs' in multiple places. Could you spell it out when it first appears? If it stands for coding DNA sequence, I think 'CDS' is a more common abbreviation.<br><br>R8. Done as suggested.
**Additional Information:**	
**Question**	**Response**
Are you submitting this manuscript to a special series or article collection?	No
**Experimental design and statistics**<br><br>Full details of the experimental design and statistical methods used should be given in the Methods section, as detailed in our Minimum Standards Reporting Checklist. Information essential to interpreting the data presented should be made available in the figure legends.<br><br>Have you included all the information requested in your manuscript?	Yes
**Resources**<br><br>A description of all resources used, including antibodies, cell lines, animals and software tools, with enough information to allow them to be uniquely identified, should be included in the Methods section. Authors are strongly encouraged to cite Research Resource Identifiers (RRIDs) for antibodies, model organisms and tools, where possible.<br><br>Have you included the information requested as detailed in our Minimum Standards Reporting Checklist?	Yes
**Availability of data and materials**<br><br>All datasets and code on which the conclusions of the paper rely must be either included in your submission or deposited in publicly available repositories (where available and ethically appropriate), referencing such data using a unique identifier in the references and in the “Availability of Data and Materials” section of your manuscript.	Yes

Have you have met the above requirement as detailed in our Minimum Standards Reporting Checklist?	

Powered by Editorial Manager® and ProduXion Manager® from Aries Systems Corporation

Manuscript

Click here to download Manuscript The genome sequence of Potentilla micrantha R2_CLEAN.docx

The genome sequence and transcriptome of *Potentilla micrantha* and their comparison to *Fragaria vesca* (the woodland strawberry)

DATA NOTE

Matteo Buti[1,7] (mbuti78@gmail.com), Marco Moretto[1] (marco.moretto@fmach.it), Elena Barghini[2] (elena.barghini@gmail.com), Flavia Mascagni[2] (flaviamascagni@gmail.com), Lucia Natali[2] (lucia.natali@unipi.it), Matteo Brilli[1,3] (matteo.brilli.bip@gmail.com), Alexandre Lomsadze[4] (alexandre.lomsadze@bme.gatech.edu), Paolo Sonego[1] (paolo.sonego@fmach.it), Lara Giongo[1] (lara.giongo@fmach.it), Michael Alonge[5] (michael.alonge@driscolls.com), Riccardo Velasco[1] (riccardo.velasco@fmach.it), Claudio Varotto[1] (claudio.varotto@fmach.it), Nada Šurbanovski[1] (surbanovski.nada@gmail.com), Mark Borodovsky[3] (borodovsky@gatech.edu), Judson A. Ward[4] (judson.ward@driscolls.com), Kristof Engelen[1] (engelen.kristof@gmail.com), Alessandro Cestaro[1] (alessandro.cestaro@fmach.it), Andrea Cavallini[2] (andrea.cavallini@unipi.it), Daniel James Sargent [1,6,*] (sargentdj@gmail.com)

[1]Fondazione Edmund Mach, Centre for Research and Innovation, via Mach 1, San Michele all'Adige, 38010 (TN), Italy

[2]Department of Agricultural, Food, and Environmental Sciences, University of Pisa, Pisa I-56124, Italy.

[3]Department of Agronomy, Food, Natural Resources, Animals and Environment, University of Padova Agripolis, V.le dell'Università 16, 35020 Legnaro (PD), Italy.

[4]Wallace H. Coulter Department of Biomedical Engineering, Georgia Tech, Atlanta, GA 30332, USA.

[5]Driscoll's Strawberry Associates, Cassin Ranch, 121 Silliman Drive, Watsonville, California, USA.

[6]Driscoll's Genetics Limited, East Malling Enterprise Centre, New Road, East Malling, Kent ME19 6BJ, UK.
[7]Center for the Development and Improvement of Agri-Food Resources (BIOGEST-SITEIA) University of Modena and Reggio Emilia, P.le Europa 1, 42124 Reggio nell'Emilia (RE), Italy
*Corresponding Author

ABSTRACT

The genus *Potentilla* is closely related to that of *Fragaria*, the economically important strawberry genus. *Potentilla micrantha* is a species that does not develop berries, but shares numerous morphological and ecological characteristics with *F. vesca*. These similarities make *P. micrantha* an attractive choice for comparative genomics studies with *F. vesca*. In this study, the *Potentilla micrantha* genome was sequenced and annotated, and RNA-Seq data from the different developmental stages of flowering and fruiting were used to develop a set of gene predictions. A 327 Mbp sequence and annotation of the genome of *P. micrantha*, spanning 2,674 sequence contigs, with an N50 size of 335,712, estimated to cover 80% of the total genome size of the species was developed. The genus *Potentilla* has a characteristically larger genome size than *Fragaria*, but the recovered sequence scaffolds were remarkably collinear at the micro-syntenic level with the genome of *F. vesca*, its closest sequenced relative. A total of 33,602 genes were predicted, and 95.1% of BUSCO genes were complete within the presented sequence. Thus, we argue that the majority, of the gene-rich regions of the genome have been sequenced. Comparisons of RNA-Seq data from the stages of floral and fruit development revealed genes differentially expressed between *P. micrantha* and *F. vesca*. The data presented are a valuable resource for future studies of berry development in *Fragaria* and the Rosaceae and they also shed light on the evolution of genome size and organization in this family.

Keywords: long-read sequencing; evolutionary development; angiosperms; genome sequence; transcriptomics;

INTRODUCTION

Potentilla, a genus of approximately 500 species [1], is closely-related to that of *Fragaria* [2], the genera having diverged from a common ancestor just 24 million years ago [3]. The genus *Fragaria*, a member of the Fragariianae tribe of the Rosaceae family, is economically-important due to the sweet, aromatic accessory fruits (berries) produced by members of the genus, in particular those of the cultivated allo-octoploid (2*n*=8×=56) strawberry species *F.* × *ananassa*. The availability of a genome sequence for a wild diploid relative of the cultivated strawberry, the woodland strawberry *F. vesca* (2*n*=2×=14) has enabled the investigation of the molecular basis of many traits of economic and academic interest in strawberry, including the development of accessory fruits. However, all members of the *Fragaria* genus produce berries, and as such the use of reverse genetics approaches to study the genes involved in berry evolution and development would require *Fragaria* mutants that do not produce fruits, a resource that is not currently available.

In the post genomics era comparative analysis permits the study of related, yet divergent species, by tracing changes at the genomic and transcriptomic levels responsible for their phenotypic differences. Previously, the sequenced genomes of *F. vesca*, *Prunus persica* and *Malus × domestica* were compared [4]; the study revealed insights into the evolutionary mechanisms that have shaped the three species, demonstrating that the *Fragaria* genome underwent significant small-scale structural rearrangement since it diverged from the common ancestor of the three genera. Comparative transcriptomics can also be used to reveal differences in the expression of orthologous genes between organisms at different stages of physiological development [5]. Such an approach suggests that comparative analyses between *Fragaria* and a closely-related species that does not bear berries may reveal important insights into the evolution of fruit development. Additionally, species separation is often related to changes in genome structure, and genome size in particular. Differences in genome

size are often the consequence of polyploidization events and/or changes in the abundance of repetitive DNA, especially transposable elements [6].

Potentilla micrantha, like the majority of species within the *Potentilla* genus does not develop accessory fruits, but shares numerous morphological characteristics with *F. vesca* (Fig. 1) including plant habit and flower morphology. Notably, they grow within the same ecological niches, and where their ranges of distribution overlap, *P. micrantha* can be found growing nearby populations of *F. vesca* (Sargent, unpublished results). These striking similarities make *P. micrantha* an attractive choice for comparative genomics studies with *F. vesca* to study the genetic basis of berry development in the latter species. As a precursor to a whole genome sequencing initiative, an initial sequencing project focused on the *P. micrantha* chloroplast was undertaken using the Illumina HiSeq and PacBio RS sequencing platforms [7].

DATA DESCRIPTION

The objectives of this study were to develop a genomic toolkit for *P. micrantha* to permit comparative genomic and transcriptomic studies with *F. vesca*, with a view to identifying the evolutionary changes that have occurred between the two species. The genome size of *P. micrantha* was determined by flow cytology and the nuclear genome was sequenced and assembled from Illumina and PacBio sequencing reads, assembled and integrated using ALLPATHS and PBJelly. Gene predictions from the *P. micrantha* genome were made with support of RNA-Seq data generated from tissue libraries sampled during flower and fruit development. The genome of *F. vesca* was compared to the sequencing scaffolds produced for *P. micrantha*, and whilst they exhibited a remarkable degree of collinearity at the micro-syntenic level, large-scale differences in transposon activity were identified that could be responsible for the large differences in genome size between the two species. The dataset we report will be useful for comparative studies of a number of traits between *P. micrantha* and its economically-important close relatives.

RESULTS

Flow cytometry, heterozygosity estimation and genome assembly

DNA was extracted from *Potentilla micrantha* young, unexpanded leaves. Flow cytometry using a *V. minor* internal standard with a DNA content of 1.52 pg/2C returned average DNA quantities of 0.52 pg/2C for *F. vesca* 'Hawaii 4' and 0.83 pg/2C for *P. micrantha* over three biological replicates. Using the calculation of Dolezel et al. (2003) [8] that 1 pg DNA is equivalent to 978 Mbp of DNA sequence, the genome size of *P. micrantha* was determined as 405.87 Mbp in length whilst that of *F. vesca* 'Hawaii 4' was calculated to be 254.28 Mbp.

Data were returned for the overlapping fragment library (OLF) and all four mate-pair libraries sequenced using Illumina HiSeq. In total, 61.4 Gbp of data were returned and the relative depth of coverage obtained for the *P. micrantha* genome from each library is given in Additional File 1: Table S1. Four different PacBio RS sequencing libraries were constructed and sequenced using two different versions of the PacBio chemistry (Additional File 2: Table S2). From the sequencing of 63 SMRT cells, 6,447,413 sequences with an average length of 2,221 bp were recovered, totaling 14.32 Gb of long read sequence data. From the data, 33× equivalent of sequence was contained in reads longer than 1 kb which were used for gap filling of the Illumina assembly using PBJelly [9].

The initial ALLPATHS assembly of the Illumina short-read sequences produced 33,026 contigs with an N50 of 16,235 bp and a total length of 247,565,733 bp. Following scaffolding, a genome assembly with a total length of 315,266,043 bp contained in 2,866 sequencing scaffolds was returned. The final scaffold set returned following ALLPATHs assembly contained a total of 0.07% ambiguous sites (SNPs), revealing the genome of *P. micrantha* to be one of the most homozygous naturally-occurring genomes sequenced to date. Following incorporation of the PacBio RS data using PBJelly [9], the *P. micrantha* sequence assembly contained 326,533,584 bp of sequence data, a 3.5% increase over the ALLPATHS Illumina assembly, in 2,674 scaffolds. The longest and N50 scaffold lengths both increased following gap filling by 9.3% and 5.1% respectively, but most significantly, the number of gapped Ns in the assembly was reduced by 59.7% to 27,311,787 (8.4% of the final assembly) (Table

1). The final scaffolded assembly contained 80.45% of the total estimated genome size for *P. micrantha* as calculated by flow cytometry. Sequence scaffold size ranged from 935 bp to 3,488,351 bp. Of the 2,674 scaffolds, 878 (32.8%) were less than 10 kbp in length, 534 (20%) were between 10 and 50 kbp in length, 738 (27.6%) were between 50 and 200 kbp in length, 500 (18.7%) contained between 200 kbp and 1 Mbp of sequence, and the remaining 23 (0.9%) contained over 1 Mbp of sequence. The majority of the 1,440 benchmarking single-copy orthologous (BUSCO) groups queried [10] were present in the genome sequence, with 95.1% (1,337 complete and single copy and 33 complete and duplicated BUSCOs) identified within the sequencing scaffolds.

Gene prediction and preliminary annotation

The results of the combined alignment of the 12 RNA-seq read sets to the *Potentilla* genome sequence scaffolds and number of splice sites identified using STAR is presented in Additional File 3: Table S3. A total of 1,908 consensus repeat sequences were generated by RepeatModeler totaling 1,431,262 bp and having a GC content of 40.8%. The total ATCG content of sequencing scaffolds greater than 10 kb in length was 298,987,576 bp. A total of 138,597,969 bp (46.36%) of the genome sequence were masked using the consensus sequences in the RepeatModeler library, including 26,359 (7.5%) of the mapped GT-AG introns identified by STAR. Gene prediction using GeneMark-ET on the masked genome identified a total of 33,602 genes, of which 32,137 were predictions containing multiple exons, and 4,655 were single exon predictions. A total of 172,791 exons were predicted, with an average length of 223 bp and an average of 5.14 exons per gene. A total of 139,216 introns were predicted in the CDS of the genes, with an average intron length of 499 bp. BUSCO analyses were compared between the gene predictions developed for *P. micrantha* and those of *F. vesca*. In total, 1,282 (89%) complete and 68 (4.7%) fragmented BUSCOs (93.75% total) were recovered for *P. micrantha*, compared to 1,303 (90.5%) complete and 79 (5.5%) fragmented BUSCOs (95.6%) recovered for *F. vesca* gene predictions indicating a similar level of completeness of the *P. micrantha*

to its nearest sequenced relative. Following a local BLAST search and BLAST2GO analysis, a total of 27,968 *P. micrantha* predicted genes were assigned a preliminary gene annotation.

Scaffold anchoring and synteny to the *Fragaria vesca* Fvb genome sequence

Following the inparanoid analysis, a total of 33,127 genes returned an orthologous relationship with one or more *F. vesca* gene predictions at the amino acid level (98.6%). A subsequent BLAST analysis of the gene predictions against the *F. vesca* v2.0 pseudomolecules identified a total of 24,641 *P. micrantha* genes that returned an unambiguous match with the position with an orthologous gene on the *F. vesca* genome. A total of 1,682 *P. micrantha* sequence scaffolds, containing 315,081,089 bp (96.5% of the total sequence) contained at least one gene that was anchored to one of the *F. vesca* v2.0 pseudomolecules. Of those, 573 contained at least ten ortholgous gene sequences, 118 contained at least 50 orthologous sequences and 32 contained over 100 ortholgous (Supplementary Excel File 1). Scaffold 'Contig145', the largest scaffold in the *P. micrantha* genome sequence (3,488,351 bp) contained the largest number of orthologous gene sequences anchored to the *F. vesca* v2.0 genome sequence (560), whilst scaffold 'Contig2191' was the smallest anchored scaffold at 1,163 bp, and containing a single orthologous gene sequence. Comparison of the two genomes revealed a remarkable degree of micro-synteny with majority of the *P. micrantha* scaffolds spanning uninterrupted regions of the *F. vesca* genome sequence (Data not shown). A very high degree of collinearity in gene order was observed between *P. micrantha* scaffolds and the *F. vesca* pseudomolecules (Fig. 2a). In general, only a small number of inversions were observed between syntenic blocks between the two genomes, and just eight *Potentilla* scaffolds contained distict syntenic blocks that aligned with more than one *Fragaria* pseudomolecule (Fig. 2b). Scaffold anchoring to a genetic map however was not performed for the *P. micrantha* genome sequence, and as such, a comparison of macrosynteny between *Fragaria* and *Potentilla* could not be made.

Gene expression during fruit development

Tissues from five stages of flowering and 'fruit' development were harvested from *Potentilla micrantha* untreated flowers in biological duplicates or triplicates for RNA isolation. The stages of flowering followed those identified in *Fragaria* by [11], with the addition of a stage 0 (unopened flowers) and young unexpanded leaf tissue. The selected developmental stages are shown in Fig. 3. RNA-libraries were made and sequenced with Illumina HiSeq2000. Following QC and adapters trimming, a total of 619,085,115 101 bp paired reads were obtained from the 12 *P. micrantha* RNA-seq libraries. Sequencing yield from individual libraries ranged from 29,653,058 to 60,158,302 reads per sample (Additional File 4: Table S4). Following trimming, the number of reads available for *Fragaria* from the published sequences of [11] were 1,236,882,540, with reads per library ranging from 109,643,225 to 155,643,061. Between 62% and 69% of *P. micrantha* filtered reads per library mapped to the *P. micrantha* gene prediction set, whilst similarly 63% to 67% of *F. vesca* filtered reads per library mapped to the *F. vesca* gene prediction set (Additional File 4: Table S4). A total of 1,556 genes were differentially expressed between the four developmental stages in at least one pair-wise comparison of the different stages in *P. micrantha*, whilst 816 genes were differentially expressed in *F. vesca* between the four stages (Fig. 4). A total of 52.44% and 43.38% of the differentially expressed genes were GO-annotated for *P. micrantha* and *F. vesca* respectively (Additional File 5: Fig. S1). Analysis of the GO terms for *F. vesca* and *P. micrantha* revealed an enrichment for genes associated with lipid metabolic processes, transporter activity, and transcription factor activity and transcription regulator activity in *F. vesca* over *P. micrantha* (Fig. 5). The gene expression profiles between the four developmental stages studied in the two species showed no clear consistent patterns between the two species overall (Additional File 6: Fig. S2), however the common differentially expressed genes displayed largely similar expression patterns (Fig. 6), with some exceptions, most noteably gene1369-v1.0-hybrid and its homologue in *P. micrantha* (17717_t), a predicted 3-hydroxy-3-methylglutaryl coenzyme A reductase 1, which was highly expressed in *F. vesca* but exhibited far lower levels of gene expression in *P. micrantha*.

Analysis of MADs-box conserved domain-containing genes in *Potentilla* and *Fragaria*

A total of 75 *P. micrantha* and 81 *F. vesca* predicted proteins containing MADS-box conserved domains were aligned and phylogenetic trees were constructed to reliably identify orthology relationships between *P. micrantha* and *F. vesca* genes. The three methods employed for phylogenetic reconstruction (ML, MP, NJ) returned largely congruent topologies for the nodes with more than 50% bootstrap support, with NJ providing a slightly more resolved tree given the use of a pairwise, instead of a partial deletion approach. Fig. 7 displays the ML phylogenetic reconstruction of the *P. micrantha* and *F. vesca* genes containing MADs-box, along with the gene expression levels for each gene (data for the NJ and MP trees are not shown). The majority of the genes were retained after the divergence of the species, indicated by a large proportion of orthologous pairs retrieved. Only a few events of lineage-specific gene loss/duplication were observed. Both observations are in line with the lack of ploidy changes within *P. micrantha* and *F. vesca* in the estimated 24.22 million years since species divergence. As expected, the majority of orthologous pairs shared similar expression patterns. Based on the ML gene tree however, three clades of orthologous genes were identified that were not expressed, or poorly expressed in *P. micrantha* but highly expressed in *F. vesca* (Fig. 8). The three clades, numbered as 1, 2 and 3 on Fig. 8**,** contained the following genes: clade 1 contained genes 27280_t (*P. micrantha*) and gene25871-v1.0-hybrid (*F. vesca*), which displayed highest homology to *A. thaliana* AGL36, a sequence-specific DNA binding transcription factor active during endosperm development [12]; clade 2 contained genes 26598_t (*P. micrantha*) and gene18483-v1.0-hybrid (*F. vesca*), whose closest *A. thaliana* homologue was AGL62, a MADS gene that promotes embryo development, indicating an essential role of endosperm cellularization for viable seed formation [13]; and clade 3 contained *P. micrantha* genes 23638_t, 23641t and 759_t and *F. vesca* genes gene32155-v1.0-hybrid and gene13277-v1.0-hybrid, whose closest *A. thaliana* homologue AGL15 delays senescence programs in perianth organs and developing fruits and alters the process of seed desiccation [14].

Analysis of the repetitive component of the *Potentilla micrantha* genome

In total, 1,001,838 of 1,484,780 reads clustered with RepeatExplorer were grouped into 107,190 clusters, representing 67.5% of the genome. No predominant repeat families were identified in the *P. micrantha* genome, with the most redundant repeat cluster representing just 1.18% of the total genome length. LTR-retrotransposons made up the main fraction (24.1%) of the *P. micrantha* genome (Additional File 7: Fig. S3), with a *Gypsy* to *Copia* ratio of approximately 2:1. Terminal-repeat retrotransposons in miniature (TRIMs) were poorly represented, making up just 0.2% of the genome, whilst putative DNA transposons accounted for 5.7% of the genome and included putative CACTA, Harbinger, and hAT elements, with other, unclassified repeats accounting for 10.6% of the genome. A comparison of the repetitive portion of the *F. vesca* and *P. micrantha* genomes performed by pairwise clustering of Illumina sequence reads revealed significant diversification between the repetitive component of the genomes of the two species (Additional File 8: Fig. S4). Among the top 291 repeat clusters that had a genome proportion >0.01%, 107 were specific to *P. micrantha*, 51 were specific to *F. vesca*, whilst only 25 were similarly represented in the two species. Among all repeat classes, only ribosomal DNAs show similar genome proportions between *P. micrantha* and *F. vesca*.

Potentilla full-length LTR-RE characterization, annotation and insertion age

Of the 505 LTR-REs characterised, 220 (43.6%) belonged to the *Copia* superfamily, with the greatest proportion belonging to the *Bianca* family, 256 (50.7%) belonged to the *Gypsy* superfamily, with the greatest proportion belonging to the *Ogre/TAT* family, whilst the remaining 29 (5.7%) could not be placed into a specific superfamily. Table 2 lists the proportion of the annotated 505 LTR-REs in each superfamily, and the numbers of elements contained in each sub-family within the *Copia* and *Gypsy* super-families. For RE insertion age determination, the mean synonymous substitution rate between *P. micrantha* and *F. vesca*, was estimated by comparing 50 orthologous genes, which equated to 52,703 bp of aligned sequences between the two species, resulting to be 0.064 synonymous substitutions per site (K_s). Using a timescale of 24.22 million years since the separation of

P. micrantha and *F. vesca*, and a K_s of 0.064, the resulting synonymous substitution rate was 2.64×10^{-9} substitutions per year. As mutation rates for LTR retrotransposons have been estimated to be approximately two-fold higher than silent site mutation rates for protein coding genes (SanMiguel and Bennetzen 1998; Ma and Bennetzen 2004), a substitution rate per year of $5.28\times10{-}9$ was used in calculations of LTR-RE insertion dates. When the whole set of usable retrotransposons was taken into account, the nucleotide distance (K) between sister LTRs showed a large degree of variation between retro-elements, ranging from 0 to 0.124 using the Kimura two parameter method, which represents a time span of at most 23.54 million years.

DISCUSSION

In this investigation, we present a set of resources for *P. micrantha*, which will form the foundation for future genomics studies in the species. Here, the genome of *P. micrantha*, a member of the Rosaceae, a diverse family of fruiting perennial plant genera, was sequenced using both short-read Illumina and long-read PacBio sequence data, and the resulting data was assembled into a highly contiguous reference sequence for the genus *Potentilla*. The study has provided a foundational resource for the future comparative genomics studies within the Rosoideae sub-family of Rosaceae in particular, but also in the Rosaceae as a whole. PacBio data (using early iterations of the sequencing chemistry) were proficiently integrated with short-reads, significantly improving the contiguity of the assembly; however the PacBio throughput was not sufficient to permit independent *de novo* assembly. Nonetheless, whilst fragmented, the genome and sequence presented here have a quality similar to the *F. vesca* genome, containing significantly fewer un-sequenced gaps within scaffolds, and is far more contiguous than that of *R. occidentalis* [15]. Along with the set of gene predictions presented, it represents a valuable resource for studying the genetic basis of a number of key morphological traits that differ between *P. micrantha* and its closest sequenced relatives.

Potentilla and *Fragaria* are separated by just 24.22 million years of evolution [3], however, in this investigation, we show the genome of *P. micrantha* is 59.6% larger than that of *F. vesca*, and it is

also larger than the available genomes of the other Fragariianae i.e. *Rubus* [16,17] and *Rosa* species [18,19] to which it is more distantly related. We also demonstrate here that *P. micrantha* and *F. vesca* exhibit a remarkable degree of microsynteny of the coding portion of the genome, with the main differences being short-range inversions. Nonetheless, the apparent differences in insertion age of transposable elements in the two genomes has led to significant differences in the repetitive portions. Whereas the genome structure of *P. micrantha* is similar to that of most angiosperm species [20], with a repetitive component amounting to around 41.5% of the total genome content, the genome of *F. vesca* has been previously demonstrated to contain just 22% repetitive elements [21]. Contrary to the coding or non-repetitive genome, the repetitive fractions of the *P. micrantha* and *F. vesca* genomes are highly diversified, suggesting that the overwhelming majority of retrotransposon activity in the genus *Potentilla* occurred after the divergence of the two genera from their common progenitor. The data presented here strongly indicate that retrotransposon activity (or the lack thereof in the genus *Fragaria*) is responsible for the significant difference between the genome size of *Fragaria* and its closest relatives, and support the assentation of Potter et al. (2007) [2] that *Fragaria* should be treated as a distinct genus, separate from *Potentilla*.

Gene expression patterns for differentially expressed genes that were common to both *F. vesca* and *P. micrantha* were largely similar between the two species, however one gene, a 3-hydroxy-3-methylglutaryl coenzyme A reductase 1 homologue displayed significantly higher gene expression levels in *F. vesca.* The 3-hydroxy-3-methylglutaryl coenzyme A reductase 1 gene catalyzes the first committed step in the cytosolic isoprenoid biosynthesis pathway [22]. Loss of function mutants of this gene in *Arabidopsis* display a dwarf phenotype due to suppression of cell elongation and reduced sterol levels [22]. Sterols are precursors in cellulose synthesis, important for cell-wall formation [23] and fruit development, and as such, up-regulation in the 3-hydroxy-3-methylglutaryl coenzyme A reductase 1 gene during fruit development in *F. vesca* over *P. micrantha* may indicate a role for this enzyme in berry formation in *Fragaria*.

In contrast to the gene expression patterns of differentially expressed genes common to both *F. vesca* and *P. micrantha* during fruit development, global patterns of gene expression during fruit development differed between the two species. The gene ontology for the *F. vesca* expression profile was enriched for genes with transcription factor and transcription regulator activity as well as transporter activity and lipid metabolic processes. A study of the differences in transcriptional regulation between *F. vesca* and *P. micrantha* therefore may provide clues to the genetic basis of berry formation in *F. vesca*. MADS-box transcription factors have been implicated in a wide and extremely diverse array of developmental processes in plants [24], and were initially demonstrated to play a major role in floral organ differentiation, including gametophyte, embryo and seed development, as well as flower and fruit development. A study of the differential expression of MADS-box genes revealed three clades of orthologous genes where gene expression of orthologous genes was up-regulated in *F. vesca* with respect to *P. micrantha*. One clade contained genes that were homologous to AGL36, a transcription factor crucial for endosperm differentiation and development [12,25]. Another clade contained genes homologous to *A. thaliana* AGL62, which likewise has been implicated in embryo development, and is thought to have an essential role of endosperm cellularization for viable seed formation [13]. The third clade contained genes homologous to AGL15 reported to have diverse roles in embryogenesis, fruit maturation, seed desiccation and the repression of floral transition [14,26], as well as being a positive regulator of the expression of mir156, a repressor of floral transition [27].

The set of genomics tools developed here for a non-fruiting relative of *F. vesca,* including a genome sequence, gene predictions and RNA-Seq data is a valuable foundational resource for more detailed future functional studies of fleshy receptacle or berry development.

METHODS

Plant material, flow cytometry and DNA isolation

A specimen of *Potentilla micrantha* was collected from Avala, Serbia in spring 2012 and subsequently used for sequencing. The plant was maintained in a growth room at a constant temperature of 24 degrees during the day and 18 degrees at night, with a 16-hour photoperiod to encourage new shoot development. Young leaves were harvested and subjected to flow cytometry by Plant Cytometry Services, NL. Measurements were taken in triplicate against a *Vicia minor* internal standard using the propidium iodide fluorescent dye. The *F. vesca* accession 'Hawaii 4' for which a whole genome sequence has been published [21] was analyzed for comparison. Prior to harvesting leaf material for DNA extraction, the plant was moved to a darkened growth chamber for 120 hours, maintaining a constant temperature of 22 degrees. DNA was extracted from young, unexpanded leaf material using the modified CTAB extraction protocol of Chen and Ronald (1999) [28], quantified using a Nanodrop spectrophotometer and Qubit fluorometer, and assessed for integrity by agarose gel electrophoresis against a λ *Hind*III size standard.

Since *P. micrantha* does not reproduce asexually from runners, a seedling population obtained from the selfing of the original mother plant was maintained from which to harvest tissue from stages of floral and fruiting development. Flowers of *P. micrantha* and *F. vesca*, along with two other *Potentilla* species, *P. reptans* and *P. indica* were treated with naphthaleneacetic acid (NAA; Sigma-Aldrich), N-1-naphthylphthalamic acid (NPA; Sigma-Aldrich), gibberellic acid (GA3; Sigma-Aldrich) and a combination of NAA and NPA, following the methods of Kang et al. (2013) [11]. Briefly, stock solutions of 50 mM NAA, 50mM NPA, and 100mM GA3 were made in ethanol and diluted with two drops of Tween 20 and water before application. The final treatment concentrations were 500 μM for NAA and GA3 and 100 μM for NPA. 50 ml of hormone solution was pipetted onto the receptacle of each emasculated flower every two days for twelve days.

Tissue sampling, RNA extraction and sequencing

Tissues from five stages of flowering and 'fruit' development were harvested from untreated flowers in biological duplicates or triplicates for RNA isolation. The stages of flowering followed those

identified in *Fragaria* by Kang et al. (2013) [11], with the addition of a stage 0 (unopened flowers) and young unexpanded leaf tissue. The selected developmental stages are shown in Fig. 3. RNA was extracted from 50 mg of snap-frozen tissue from each developmental stage using the Spectrum plant total RNA extraction kit (Sigma) with an on-column DNase I digestion (Sigma) step. The extraction protocol followed the manufacturers' recommendations with two minor modifications: 1% PVP was added to the lysis solution, and the number of washes at each stage was doubled (i.e. two washes were performed with wash solution 1 and four washes were performed with wash solution 2). The RNA extracted from each sample was diluted in 50 µl of elution solution (Sigma). Following elution, total RNA was quantified using a Nanodrop spectrophotometer and Qubit fluorometer and assessed for integrity using a Bioanalyzer (Agilent). Samples returning a RIN value greater than 7.5 were considered acceptable for sequencing. A total of 12 Illumina TruSeq libraries were constructed from 2 µg of total RNA. Libraries were made from the following samples; one from stage 0, two from stage 1, two from stage 2, three from stage 3 and three from stage 4. A final library was made from RNA of young leaf tissue. The libraries were sequenced in triplex per single lane of Illumina HiSeq2000. Samples were indexed and multiplexed, and then 101 bp paired-end sequencing was performed using the Illumina HiSeq 2000 platform at the Weill Medical core genomics facility of Cornell University.

Whole genome shotgun sequencing, assembly

A strategy following the ALLPATHs-LG protocol was followed to produce an initial assembly using second-generation sequence data. Five sequencing libraries were developed; an overlapping fragment library (OLF) with an insert size of 170 bp, and four libraries of 3 kb, 5 kb, 8 kb and 12 kb. The OLF library was created using the Illumina Nextera library preparation kit following the manufacturers' recommendations and was sequenced in simplex on a single lane of Illumina HiSeq2000, whilst the MP libraries were prepared using the Illumina Mate Pair Library v2 kit following the manufacturers' recommendations and were subsequently sequenced in duplex. All sequencing was performed at the

Weill Medical Centre core genomics facility at Cornell University. ALLPATHS-LG [29] was run using the sequencing libraries described above using default settings. Subsequently, a selection of SMRT-bell sequencing libraries were constructed using various versions of the PacBio RS sequencing kits and chemistries (Additional File 2: Table S2) and PBJelly [9] running default settings was used to incorporate data generated using the PacBio RS platform (Pacific Biosciences) into the ALLPATHS-LG Illumina assembly scaffolds. Identification of benchmarking universal single-copy orthologs (BUSCOs) was performed using BUSCO v3 [10] running default parameters and using 1,440 BUSCO groups from the embryophyta_odb9 (plant) lineage data.

Gene prediction, annotation, determination of gene orthology and evaluation of synteny between *Potentilla* and *Fragaria* genomes

First, *ab initio* repeat finding was done with RepeatModeler [30] that was run on the complete set of genomic scaffolds set and a repeat library was created. Next, the genome was masked using RepeatMasker [31]. Gene prediction was done with GeneMark-ET [32]. The following parameters were used; a minimum scaffold length of 10 kb, a maximum scaffold gap size of 40 kb, a minimum intron size of 50 bp, a maximum intron length of 10 kb and a maximum intergenic length of 50 kb. RNA-seq reads from the 12 libraries were aligned to the genome sequence scaffolds using the STAR tool with default parameters [33]. Reads from the 12 RNA-seq datasets were aligned to the genome. Mapping of RNA-seq reads that included intron junctions led to the identification of introns. Introns with a high 'intron score' (identified by more than 60 RNAseq reads) were considered to be reliably identified. Predicted genes were annotated using BLAST2GO [34]. The non-redundant NCBI protein database was downloaded and BLAST was run locally. Results from the BLAST analysis were uploaded to the BLAST2GO server and gene ontology analyses were performed using default parameters.

Orthologous relationships between *Fragaria* and *Potentilla* genes was determined through sequence clustering performed using Inparanoid 7 [35]. Analyses were based only on homology, as an

alternative to the more stringent ortholog classification. *Prunus persica* v2.0.a1 predicted proteins downloaded from the GDR [36] and *Potentilla micrantha* and *Fragaria vesca* protein sequences were blasted all against all and the output file was filtered at the following thresholds: maximum E-value=10^{-4} and query coverage of at least 50%. The resulting file was used as an input to the MCL algorithm using as edge weight $-\log_{10}$(evalue) (all E-values=0 were changed to 1E-300). To explore more thoroughly the homology network used as input, the MCL algorithm was run at different granularity levels (inflation parameter equal to 1.5, 1.7, 2.0, 2.3, 2.4, 2.7, 3) and then a table indicating cluster memberships at the different stringencies was compiled for each node. Ortholog classification was produced using Inparanoid 7 [35] for pairs of species in all combinations. The resulting sqltables were then used as an input for QuickParanoid (http://pl.postech.ac.kr/QuickParanoid/) and the sequences were combined in a three-species ortholog classification. The clusters obtained with QuickParanoid were used to calculate the number of genes contained in each cluster for both *Potentilla* and *Fragaria*.

Potentilla gene predictions for which an orthologous relationship was identified through the inparanoid analysis, were used as queries to identify the physical locations of ortholgous sequences on the *F. vesca* v2.0 pseudomolecules and those sequences that returned a single, unambiguous match on the genome sequence were used to evaluate synteny between the two species. Since the *Potentilla* genomic scaffolds were not oriented and ordered against a reference genetic map, conservation of synteny between the *Potentilla* and *Fragaria* genomes was determined through a comparison of the physical positions of orthologous gene sequences on the sequence scaffolds of *Potentilla* and the pseudomolecules of *Fragaria*. Criteria for the identification of syntenic regions followed that of Jung et al (2012). No attempt was therefore made to infer macro-syntenic structure on a chromosome scale between the two genomes.

Gene expression during stages of fruit development in *Potentilla micrantha* and *Fragaria vesca*

The quality of the raw reads generated as described above was checked with FastQC [37]; Trimmomatic [38] was used to remove adapter sequences. The *F. vesca* .sra files [11] were used to compare gene expression in *Fragaria* with *Potentilla*; *Fragaria* reads from the same developmental stage were merged and treated as a single data set since data from *Potentilla* was not generated from individual floral organs. The 12 trimmed *P. micrantha* RNA-seq libraries were mapped on the *P. micrantha* gene prediction CDS, while the ten *F. vesca* sets were mapped to the *F. vesca* v1.0 gene prediction CDS [21] downloaded from the GDR [36] using Bowtie2 [39] and default settings. The number of reads mapping to each gene for each RNA set was calculated from the .sam alignment files derived from Bowtie2.

Counts of RNA-seq reads over transcripts were used to calculate the gene expression level in $FPKM=10^9*ER/(EL \times MR)$, where ER was the number of mapped reads in the exons of a particular gene, EL was the sum of exon length in base pairs, and MR was the total number of mapped reads [40]. FPKM was used to distinguish expressed genes from inactive genes (those not returning any expression data) during the flower development in each species. Further, FPKM was used to define a set of highly expressed genes: Genes were considered as 'highly-expressed' if FPKM>1000. Genes that returned an FPKM<1000 in all samples were removed from further differential expression analysis. The retained differentially expressed genes were processed by performing a linear rescaling of the log2-counts, aligning the distributions for every sample at their distribution modes, followed by variance stabilization to ensure homoscedasticity. A one-way ANOVA was performed gene-by-gene on the rescaled $\log_2$-counts to detect changes in expression among different developmental phases. Differentially expressed genes (DEGs) were selected by setting cutoffs both on the p-values from the ANOVA F-tests, as well as on the magnitude of observed changes represented by the square root of the ANOVA MSR values (equivalent to using volcano plots for two-condition studies). Genes were considered differentially expressed if the sqrt (MSR) > 2.00 and p-value < 10^{-3}.

Gene Onthology enrichment analysis of DEGs sets of *Potentilla micrantha* and *Fragaria vesca* was carried out using Blast2GO 2.8.0 [41] with "Fisher's exact test" method, considering as "enriched"

the GO categories with FDR<0.05. *Potentilla micrantha* whole transcriptome functional annotation obtained in this work was used as background for *Potentilla* GO enrichment analysis, while the “InterPro GO for GeneMark hybrid transcripts” database downloaded from GDR website was used as background for *Fragaria vesca*. Cytoscape 3.5.1 [42] with the BiNGO 3.0.3 plugin was used for the GO-slim network visualization of enriched GO categories over *Fragaria vesca* and *Potentilla micrantha* DEGs. For determination of over-representation, the Benjamini and Hochberg FDR-adjusted significance level cutoff was 0.05.

Phylogenetic and functional analysis of MADs-box domain-containing genes and gene expression profile mapping

Protein sequences of *Potentilla* (this publication) and *Fragaria* (Fvesca_v1.0_hybrid; www.rosaceae.org) were analysed on the NCBI conserved domain database [43]. All proteins containing a MADS-box domain were retrieved and the MADS-box extracted with Bedtools getfasta [44] using default parameters. An initial sequence alignment was carried out using ClustalW and pairwise distances were calculated to eliminate outliers. A total of 16 sequences were removed from further analysis since they were too short and possessed incomplete N-terminal ends, indicating they were likely pseudogenes. The alignment used for phylogenetic analysis was constructed with SATé-II [45] and contained 156 protein sequences (75 from *Potentilla* and 81 from *Fragaria*).

Three methods, Maximum Likelihood (ML), Maximum Parsimony (MP) and Neighbour-joining (NJ), each with 1,000 bootstrap replicates were employed for phylogenetic reconstruction of the MADs-box domain containing genes using Mega 7.0.14 [46]. Where missing data was present in the alignment, deletion of columns containing a fraction of missing data above 10% and 30% was performed for ML and MP methods. Pairwise deletion was instead used in the case of NJ, to maximise the phylogenetic information retained in the alignment. The ML topology was used as reference for further analysis.

The expression profiles of the genes containing a MADS-box were used to decorate the phylogenetic tree using iTOL v2 [47], allowing the identification of orthologous MADS-box gene pairs displaying differential gene expression profiles between *Potentilla* and *Fragaria*. Curated annotation of differentially expressed putative gene function was carried out using BLASTp homology searches of the TAIR database [48].

Analysis of the repetitive component of *Potentilla* genome

To identify and characterize genomic repeats in the *P. micrantha* genome, a reduced set of 2,000,000 randomly selected genomic Illumina reads, corresponding to 0.57× of the *P. micrantha* genome were subjected to clustering using RepeatExplorer [49]. Among the clusters produced, the top clusters, with a genome proportion higher than 0.01%, were annotated using 0.2 as cutoff for cluster connection through mates. Clusters that were annotated as similar to phi-X174 were removed as contaminants. The output of RepeatExplorer was also used to prepare an in-house library containing all contigs belonging to clusters annotated by RepeatExplorer as long terminal repeat retrotransposons (LTR-REs) by similarity search against RepBase [50]. Subsequently, pairwise hybrid clustering between a random set of 1,431,114 Illumina reads derived from *P. micrantha* genomic DNA and 1,090,102 *F. vesca* genomic reads, each corresponding to 0.41× of the respective genomes was performed using RepeatExplorer [49].

Potentilla full-length LTR-RE characterization

LTR-FINDER [51] was used to isolate putative full-length LTR-REs from 280 randomly-selected *Potentilla* genome sequence scaffolds and alignment boundaries were obtained by adjusting the ends of LTR-pair candidates using the Smith–Waterman algorithm. These boundaries were re-adjusted based on the occurrence of the following typical LTR-RE features: (a) the putative LTR-RE were flanked by the dinucleotides TG and CA at 5′ and 3′ ends respectively; (b) a target-site duplication (TSD) of 4–6 nt in length was present in the sequence; (c) a putative 15–18 nt primer binding site

(PBS) complementary to a tRNA at the end of the putative 5′-LTR was present in the sequence; and (d) a 20–25-nt polypurine tract (PPT) just upstream of the 5′ end of the 3′ LTR was present in the sequence. Putative LTR-REs were manually validated using DOTTER [52], verifying the occurrence of LTRs, dinucleotides TG and CA at the 5′ and 3′ ends respectively, and TSDs. The validated LTR-REs were annotated using BLASTX and BLASTN querying the NCBI nr nucleotide and protein NCBI databases and RepBase [50]. To limit false-positive detection, a fixed E-value threshold of $E < 10^{-5}$ for BLASTN and $E < 10^{-10}$ for BLASTX was used. The full-length elements identified were analysed using RepeatExplorer [49], performing searches for GAG, protease, retrotranscriptase, RNAseH, integrase, and chromodomain derived from plant protein domains from RepBase. The similarity search was filtered at E-value $< 10^{-10}$, allowing for both mismatches and frameshifts. The same tool was used to assign full-length elements to specific *Gypsy* or *Copia* lineages. Full-length LTR-REs that were identified as belonging to *Gypsy* or *Copia* superfamilies, and clusters annotated as LTR-retrotransposons by RepeatExplorer (see above) were then used as reference datasets for further searches in order to identify previously unclassified elements using RepeatMasker, running default parameters, but with -div set to 20.

For determination of RE redundancy, approximately 32,000,000 randomly-selected raw *Potentilla* Illumina paired end reads, corresponding to 10.3× genome coverage. After removal of organellar contamination performed by mapping the reads to an in-house Rosaceae organellar database and the removal of duplicate reads, a total of 25,206,510 filtered nuclear reads corresponding to 7.2× equivalent genomic coverage were used for redundancy analysis by mapping the reads to all REs characterized in the *Potentilla* genome using CLC-BIO Genomic Workbench 8.0 (CLC-BIO, Aarhus, Denmark). Mismatch cost, deletion cost, and insertion cost were fixed at 1, and similarity and length fraction were both fixed at 0.9, 0.8, 0.5 or 0.4 to obtain high, medium, low, or very low stringencies, respectively. As reads that mapped to multiple distinct sequences were few, and distributed randomly throughout the dataset, the number of reads mapping to each RE was taken as the degree of redundancy of that sequence within the genome. The effective abundance of a particular class of reads

was calculated as the proportion of the total number of reads mapped in each class, with respect to the overall number of genomic reads mapped, using optimal stringency parameters, i.e. where further relaxation of stringency did not significantly increase the number of mapped reads.

The abundance of each single RE sequence in the genome was analysed by mapping *Potentilla* DNA reads, corresponding to 2× genome coverage to the full-length REs characterised, one by one using BWA (alignment via Burrows–Wheeler transformation) version 0.7.5a-r405 [53] running the following parameters: bwaaln -t 4 -l 12 -n 4 -k 2 -o 3 -e 3 -M 2 -O 6 -E 3. The resulting single-end mappings were resolved via the samse module of BWA, and the output was converted to .bam file format using SAMtools version 0.1.19 [54]. Subsequently, SAMtools was used to calculate the number of mapped reads for each alignment using the following parameters: samtools view -c -F 4.

Determination of RE insertion age

Retrotransposon insertion age was estimated through a sequence divergence comparison of the 5′- and 3′-LTRs of each putative full-length retrotransposon. Synonymous substitution rates were calculated for 50 pairs of orthologous gene sequences of *P. micrantha* and *F. vesca*, using a time of divergence of 24.22 million years [3]. Subsequently, the two LTRs were aligned with ClustalX software [53], indels were eliminated, and the number of nucleotide substitutions was counted using DnaSP [54] for each retrotransposon. The insertion times of retrotransposons with both LTRs were dated using the Kimura two parameter (K2P) method [55], calculated using DnaSP, and a synonymous substitution rate that is twofold that calculated for genes [56,57].

AVAILABILITY OF SUPPORTING DATA AND MATERIALS

The data set supporting the results of this article are available in the GenBank repository, project number PRJEB18433. The genome reference sequence and gene predictions can be downloaded from the GigaScience GigaDB repository.

FUNDING

This work was funded by a grant to the Fondazione Edmund Mach (FEM) from the Autonomous Province of Trento grants office. A.C. acknowledges funding from the Department of Agriculture, Food and Environment of Pisa University, Project 'Plantomics'.

CONFLICT OF INTERESTS

The authors declare no competing interests.

AUTHOR CONTRIBUTIONS

M.Buti performed the experiments, analysed and interpreted all data and authored the paper. M.M., P.S. and A.C. analysed sequence data and performed genome assemblies. K.E. and M. Brilli assisted with experimental design, analysed and interpreted gene expression data and commented on and contributed to the manuscript. L.N. and A.C. performed full-length retrotransposon isolation. E.B., F.M. and A.C. performed clustering, annotation and redundancy analyses of repetitive sequences. E.B., F.M., L.N. and A.C. participated in the interpretation and discussion of results and contributed to the writing of the paper. A.L and M.Borodovsky performed gene predictions and analysed and interpreted the data. L.G., N.Š. assisted with experiments, interpreted data and contributed to the manuscript. M.A. and J.W. assisted with genome assemblies and gene annotation. C.V. analysed and interpreted phylogenetic data and contributed to the manuscript. R.V. commented on the manuscript. D.J.S. designed the study, assisted with the experiments, analysed and interpreted the data and authored the paper.

ADDITIONAL FILES

Additional File 1: Table S1. Illumina sequencing libraries used in the sequencing of the *Potentilla micrantha* genome including fragment sizes and total genome depth of coverage.

Additional File 2: Table S2. PacBio RS sequencing kits and chemistries used for *Potentilla micrantha* sequencing.

Additional File 3: Table S3. RNAseq read data used for gene prediction and number of splice sites identified in the *Potentilla micrantha* genome.

Additional File 4: Table S4. *Potentilla micrantha* and *Fragaria vesca* RNAseq reads statistics.

Additional File 5: Fig S1. Distribution of predicted genes *Potentilla micrantha* and *Fragaria vesca* mapped, blasted and GO-annotated by BLAST2GO analysis.

Additional File 6: Fig S2. The differential gene expression profiles between the four developmental stages of fruit development studied in *F. vesca* and *P. micrantha*.

Additional File 7: Fig S3. The overall abundance of different classes of transposons within the *Potentilla micrantha* genome according to the analyses performed using RepeatExplorer.

Additional File 8: Fig S4. Genome proportion in *Potentilla micrantha* and *Fragaria vesca* of 291 repeats clustered using RepeatExplorer. Other repeats include satellite DNAs, pararetroviruses, and one LINE.

REFERENCES

1. Eriksson T, Donoghue MJ, Hibbs MS. Phylogenetic analysis of Potentilla using DNA sequences of nuclear ribosomal internal transcribed spacers (ITS), and implications for the classification of Rosoideae (Rosaceae). Plant Syst. Evol. [Internet]. Springer-Verlag; 1998 [cited 2016 Aug 9];211:155–79. Available from: http://link.springer.com/10.1007/BF00985357

2. Potter D, Eriksson T, Evans RC, Oh S, Smedmark JEE, Morgan DR, et al. Phylogeny and classification of Rosaceae. Plant Syst. Evol. [Internet]. 2007 [cited 2015 Oct 3];266:5–43. Available from: http://link.springer.com/10.1007/s00606-007-0539-9

3. Njuguna W, Liston A, Cronn R, Ashman T-L, Bassil N. Insights into phylogeny, sex function and age of Fragaria based on whole chloroplast genome sequencing. Mol. Phylogenet. Evol. 2013;66:17–29.

11. Kang C, Darwish O, Geretz A, Shahan R, Alkharouf N, Liu Z. Genome-Scale Transcriptomic Insights into Early-Stage Fruit Development in Woodland Strawberry Fragaria vesca. Plant Cell [Internet]. 2013;25:1960–78. Available from: http://www.plantcell.org/cgi/doi/10.1105/tpc.113.111732

21. Shulaev V, Sargent DJ, Crowhurst RN, Mockler TC, Folkerts O, Delcher AL, et al. The genome of woodland strawberry (Fragaria vesca). Nat. Genet. [Internet]. 2011 [cited 2016 Aug 8];43:109–16. Available from: http://www.ncbi.nlm.nih.gov/pubmed/21186353

4. Jung S, Cestaro A, Troggio M, Main D, Zheng P, Cho I, et al. Whole genome comparisons of Fragaria, Prunus and Malus reveal different modes of evolution between Rosaceous subfamilies. BMC Genomics [Internet]. 2012 [cited 2016 Aug 8];13:129. Available from: http://www.ncbi.nlm.nih.gov/pubmed/22475018

5. Davidson RM, Gowda M, Moghe G, Lin H, Vaillancourt B, Shiu S-H, et al. Comparative transcriptomics of three Poaceae species reveals patterns of gene expression evolution. Plant J. [Internet]. 2012 [cited 2016 Aug 8];71:492–502. Available from: http://www.ncbi.nlm.nih.gov/pubmed/22443345

6. Jiao Y, Leebens-Mack J, Ayyampalayam S, Bowers JE, McKain MR, McNeal J, et al. A genome triplication associated with early diversification of the core eudicots. Genome Biol. [Internet]. BioMed Central; 2012 [cited 2017 Feb 16];13:R3. Available from: http://genomebiology.biomedcentral.com/articles/10.1186/gb-2012-13-1-r3

7. Ferrarini M, Moretto M, Ward JA, Šurbanovski N, Stevanović V, Giongo L, et al. An evaluation of the PacBio RS platform for sequencing and de novo assembly of a chloroplast genome. BMC Genomics [Internet]. BioMed Central; 2013 [cited 2016 Aug 8];14:670. Available from: http://bmcgenomics.biomedcentral.com/articles/10.1186/1471-2164-14-670

8. Dolezel J, Bartos J, Voglmayr H, Greilhuber J. Letter to the editor. Cytometry [Internet]. Wiley Subscription Services, Inc., A Wiley Company; 2003 [cited 2016 Aug 9];51A:127–8. Available from: http://doi.wiley.com/10.1002/cyto.a.10013

9. English AC, Richards S, Han Y, Wang M, Vee V, Qu J, et al. Mind the Gap: Upgrading Genomes with Pacific Biosciences RS Long-Read Sequencing Technology. Liu Z, editor. PLoS One [Internet]. Public Library of Science; 2012 [cited 2016 Aug 8];7:e47768. Available from: http://dx.plos.org/10.1371/journal.pone.0047768

10. Simão FA, Waterhouse RM, Ioannidis P, Kriventseva E V., Zdobnov EM. BUSCO: assessing genome assembly and annotation completeness with single-copy orthologs. Bioinformatics [Internet]. 2015 [cited 2017 Nov 2];31:3210–2. Available from: http://www.ncbi.nlm.nih.gov/pubmed/26059717

12. Day RC, Herridge RP, Ambrose BA, Macknight RC. Transcriptome Analysis of Proliferating Arabidopsis Endosperm Reveals Biological Implications for the Control of Syncytial Division, Cytokinin Signaling, and Gene Expression Regulation. PLANT Physiol. [Internet]. American Society of Plant Biologists; 2008 [cited 2016 Aug 10];148:1964–84. Available from: http://www.plantphysiol.org/cgi/doi/10.1104/pp.108.128108

13. Hehenberger E, Kradolfer D, Köhler C. Endosperm cellularization defines an important developmental transition for embryo development. Development [Internet]. 2012 [cited 2016 Aug 10];139:2031–9. Available from: http://www.ncbi.nlm.nih.gov/pubmed/22535409

14. Fang S-C, Fernandez DE. Effect of regulated overexpression of the MADS domain factor AGL15 on flower senescence and fruit maturation. Plant Physiol. [Internet]. 2002 [cited 2016 Aug 10];130:78–89. Available from: http://www.ncbi.nlm.nih.gov/pubmed/12226488

15. VanBuren R, Bryant D, Bushakra JM, Vining KJ, Edger PP, Rowley ER, et al. The genome of black raspberry (Rubus occidentalis). Plant J. [Internet]. 2016 [cited 2016 Aug 16]; Available from: http://www.ncbi.nlm.nih.gov/pubmed/27228578

16. Dickson EE, Arumuganathan K, Kresovich S, Doyle JJ, Kresovich S, Doyle2 JJ. Nuclear DNA Content Variation within the Rosaceae NUCLEAR DNA CONTENT VARIATION WITHIN THE ROSACEAE'. Am. J. Bot. Am. J. Bot. Am. J. Bot. [Internet]. 1992 [cited 2016 Nov 5];79:1081–6. Available from: http://scholarcommons.sc.edu/biol_facpub

17. Meng R, Finn C. Determining Ploidy Level and Nuclear DNA Content in Rubus by Flow Cytometry. J. Am. Soc. Hortic. Sci. American Society for Horticultural Science; 2002;127:767–75.

18. Rajapakse S, Byrne DH, Zhang L, Anderson N, Arumuganathan K, Ballard RE. Two genetic linkage maps of tetraploid roses. TAG Theor. Appl. Genet. [Internet]. Springer-Verlag; 2001 [cited 2016 Nov 5];103:575–83. Available from: http://link.springer.com/10.1007/PL00002912

19. Yokoya K, Roberts A V., Mottley J, Lewis R, Brandham PE. Nuclear DNA Amounts in Roses. Ann. Bot. [Internet]. Oxford University Press; 2000 [cited 2016 Nov 5];85:557–61. Available from: http://aob.oxfordjournals.org/cgi/doi/10.1006/anbo.1999.1102

20. Vitte C, Fustier M-A, Alix K, Tenaillon MI. The bright side of transposons in crop evolution. Brief. Funct. Genomics [Internet]. Oxford University Press; 2014 [cited 2016 Aug 15];13:276–95. Available from: http://www.ncbi.nlm.nih.gov/pubmed/24681749

22. Suzuki M, Kamide Y, Nagata N, Seki H, Ohyama K, Kato H, et al. Loss of function of 3-hydroxy-3-methylglutaryl coenzyme A reductase 1 (HMG1) in Arabidopsis leads to dwarfing, early senescence and male sterility, and reduced sterol levels. Plant J. [Internet]. 2004 [cited 2017 Nov 2];37:750–61. Available from: http://www.ncbi.nlm.nih.gov/pubmed/14871314

23. Schrick K, Debolt S, Bulone V. Deciphering the molecular functions of sterols in cellulose biosynthesis. Front. Plant Sci. [Internet]. Frontiers Media SA; 2012 [cited 2017 Nov 2];3:84. Available from: http://www.ncbi.nlm.nih.gov/pubmed/22639668

24. Smaczniak C, Immink RGH, Angenent GC, Kaufmann K, Adamczyk BJ, Fernandez DE, et al. Developmental and evolutionary diversity of plant MADS-domain factors: insights from recent studies. Development [Internet]. Oxford University Press for The Company of Biologists Limited; 2012 [cited 2016 Aug 15];139:3081–98. Available from: http://www.ncbi.nlm.nih.gov/pubmed/22872082

25. Shirzadi R, Andersen ED, Bjerkan KN, Gloeckle BM, Heese M, Ungru A, et al. Genome-wide transcript profiling of endosperm without paternal contribution identifies parent-of-origin-dependent regulation of AGAMOUS-LIKE36. PLoS Genet. [Internet]. 2011 [cited 2016 Aug

16];7:e1001303. Available from: http://www.ncbi.nlm.nih.gov/pubmed/21379330

26. Harding EW, Tang W, Nichols KW, Fernandez DE, Perry SE. Expression and maintenance of embryogenic potential is enhanced through constitutive expression of AGAMOUS-Like 15. Plant Physiol. [Internet]. 2003 [cited 2016 Aug 16];133:653–63. Available from: http://www.ncbi.nlm.nih.gov/pubmed/14512519

27. Serivichyaswat P, Ryu H-S, Kim W, Kim S, Chung KS, Kim JJ, et al. Expression of the floral repressor miRNA156 is positively regulated by the AGAMOUS-like proteins AGL15 and AGL18. Mol. Cells [Internet]. Korean Society for Molecular and Cellular Biology; 2015 [cited 2016 Aug 16];38:259–66. Available from: http://www.ncbi.nlm.nih.gov/pubmed/25666346

28. Chen D-H, Ronald PC. A Rapid DNA Minipreparation Method Suitable for AFLP and Other PCR Applications. Plant Mol. Biol. Report. [Internet]. Kluwer Academic Publishers; 1999 [cited 2016 Aug 8];17:53–7. Available from: http://link.springer.com/10.1023/A:1007585532036

29. Butler J, MacCallum I, Kleber M, Shlyakhter IA, Belmonte MK, Lander ES, et al. ALLPATHS: de novo assembly of whole-genome shotgun microreads. Genome Res. [Internet]. 2008 [cited 2016 Aug 8];18:810–20. Available from: http://www.ncbi.nlm.nih.gov/pubmed/18340039

30. Smit AFA, Hubley R. RepeatModeler - 1.0.7 [Internet]. 2013. Available from: http://www.repeatmasker.org/RepeatModeler.html

31. Smit A, Hubley R, Green P. RepeatMasker Open-4.0 [Internet]. 2013. Available from: http://www.repeatmasker.org/

32. Lomsadze A, Burns PD, Borodovsky M. Integration of mapped RNA-Seq reads into automatic training of eukaryotic gene finding algorithm. Nucleic Acids Res. [Internet]. Oxford University Press; 2014 [cited 2016 Aug 8];42:e119. Available from: http://www.ncbi.nlm.nih.gov/pubmed/24990371

33. Dobin A, Davis CA, Schlesinger F, Drenkow J, Zaleski C, Jha S, et al. STAR: ultrafast universal RNA-seq aligner. Bioinformatics [Internet]. Oxford University Press; 2013 [cited 2016 Aug 8];29:15–21. Available from: http://www.ncbi.nlm.nih.gov/pubmed/23104886

34. Conesa A, Götz S. Blast2GO: A comprehensive suite for functional analysis in plant genomics. Int. J. Plant Genomics [Internet]. Hindawi Publishing Corporation; 2008 [cited 2016 Aug 8];2008:619832. Available from: http://www.ncbi.nlm.nih.gov/pubmed/18483572

35. Ostlund G, Schmitt T, Forslund K, Köstler T, Messina DN, Roopra S, et al. InParanoid 7: new algorithms and tools for eukaryotic orthology analysis. Nucleic Acids Res. [Internet]. 2010 [cited 2016 Aug 10];38:D196-203. Available from: http://www.ncbi.nlm.nih.gov/pubmed/19892828

36. Jung S, Staton M, Lee T, Blenda A, Svancara R, Abbott A, et al. GDR (Genome Database for Rosaceae): integrated web-database for Rosaceae genomics and genetics data. Nucleic Acids Res. [Internet]. Oxford University Press; 2008 [cited 2016 Aug 9];36:D1034-40. Available from: http://www.ncbi.nlm.nih.gov/pubmed/17932055

37. Andrews S. Babraham Bioinformatics - FastQC A Quality Control tool for High Throughput Sequence Data [Internet]. 2010. Available from: http://www.bioinformatics.babraham.ac.uk/projects/fastqc/

38. Bolger AM, Lohse M, Usadel B. Trimmomatic: a flexible trimmer for Illumina sequence data. Bioinformatics [Internet]. Oxford University Press; 2014 [cited 2016 Aug 9];30:2114–20. Available from: http://www.ncbi.nlm.nih.gov/pubmed/24695404

39. Langmead B, Salzberg SL. Fast gapped-read alignment with Bowtie 2. Nat. Methods [Internet]. NIH Public Access; 2012 [cited 2016 Aug 8];9:357–9. Available from: http://www.ncbi.nlm.nih.gov/pubmed/22388286

40. Mortazavi A, Williams BA, McCue K, Schaeffer L, Wold B. Mapping and quantifying mammalian transcriptomes by RNA-Seq. Nat. Methods [Internet]. Nature Publishing Group; 2008 [cited 2016 Aug 8];5:621–8. Available from: http://www.nature.com/doifinder/10.1038/nmeth.1226

41. Conesa A, Götz S. Blast2GO: A comprehensive suite for functional analysis in plant genomics. Int. J. Plant Genomics [Internet]. Hindawi Publishing Corporation; 2008 [cited 2016 Aug 8];2008:619832. Available from: http://www.ncbi.nlm.nih.gov/pubmed/18483572

42. Shannon P, Markiel A, Ozier O, Baliga NS, Wang JT, Ramage D, et al. Cytoscape: a software environment for integrated models of biomolecular interaction networks. Genome Res. [Internet]. Cold Spring Harbor Laboratory Press; 2003 [cited 2017 Nov 3];13:2498–504. Available from: http://www.ncbi.nlm.nih.gov/pubmed/14597658

43. Marchler-Bauer A, Derbyshire MK, Gonzales NR, Lu S, Chitsaz F, Geer LY, et al. CDD: NCBI's conserved domain database. Nucleic Acids Res. [Internet]. 2015 [cited 2016 Aug 8];43:D222-6. Available from: http://www.ncbi.nlm.nih.gov/pubmed/25414356

44. Quinlan AR, Hall IM. BEDTools: a flexible suite of utilities for comparing genomic features. Bioinformatics [Internet]. 2010 [cited 2016 Aug 8];26:841–2. Available from: http://www.ncbi.nlm.nih.gov/pubmed/20110278

45. Liu K, Warnow TJ, Holder MT, Nelesen SM, Yu J, Stamatakis AP, et al. SATe-II: very fast and accurate simultaneous estimation of multiple sequence alignments and phylogenetic trees. Syst. Biol. [Internet]. Oxford University Press; 2012 [cited 2016 Aug 9];61:90–106. Available from: http://www.ncbi.nlm.nih.gov/pubmed/22139466

46. Kumar S, Stecher G, Tamura K. MEGA7: Molecular Evolutionary Genetics Analysis Version 7.0 for Bigger Datasets. Mol. Biol. Evol. [Internet]. 2016;33:1870–4. Available from: https://academic.oup.com/mbe/article-lookup/doi/10.1093/molbev/msw054

47. Letunic I, Bork P. Interactive Tree Of Life v2: online annotation and display of phylogenetic trees made easy. Nucleic Acids Res. [Internet]. Oxford University Press; 2011 [cited 2016 Aug 8];39:W475-8. Available from: http://www.ncbi.nlm.nih.gov/pubmed/21470960

48. Huala E, Dickerman AW, Garcia-Hernandez M, Weems D, Reiser L, LaFond F, et al. The Arabidopsis Information Resource (TAIR): a comprehensive database and web-based information retrieval, analysis, and visualization system for a model plant. Nucleic Acids Res. [Internet]. Oxford University Press; 2001 [cited 2016 Aug 8];29:102–5. Available from: http://www.ncbi.nlm.nih.gov/pubmed/11125061

49. Novák P, Neumann P, Pech J, Steinhaisl J, Macas J. RepeatExplorer: a Galaxy-based web

server for genome-wide characterization of eukaryotic repetitive elements from next-generation sequence reads. Bioinformatics [Internet]. 2013 [cited 2016 Aug 9];29:792–3. Available from: http://www.ncbi.nlm.nih.gov/pubmed/23376349

50. Jurka J, Kapitonov VV, Pavlicek A, Klonowski P, Kohany O, Walichiewicz J. Repbase Update, a database of eukaryotic repetitive elements. Cytogenet. Genome Res. [Internet]. Karger Publishers; 2005 [cited 2016 Aug 9];110:462–7. Available from: http://www.karger.com/?doi=10.1159/000084979

51. Xu Z, Wang H. LTR_FINDER: an efficient tool for the prediction of full-length LTR retrotransposons. Nucleic Acids Res. [Internet]. Oxford University Press; 2007 [cited 2016 Aug 8];35:W265-8. Available from: http://www.ncbi.nlm.nih.gov/pubmed/17485477

52. Sonnhammer EL, Durbin R. A dot-matrix program with dynamic threshold control suited for genomic DNA and protein sequence analysis. Gene [Internet]. 1995 [cited 2016 Aug 8];167:GC1-10. Available from: http://www.ncbi.nlm.nih.gov/pubmed/8566757

53. Li H, Durbin R. Fast and accurate short read alignment with Burrows-Wheeler transform. Bioinformatics [Internet]. 2009 [cited 2016 Aug 9];25:1754–60. Available from: http://www.ncbi.nlm.nih.gov/pubmed/19451168

54. Li H, Handsaker B, Wysoker A, Fennell T, Ruan J, Homer N, et al. The Sequence Alignment/Map format and SAMtools. Bioinformatics [Internet]. 2009 [cited 2016 Aug 9];25:2078–9. Available from: http://www.ncbi.nlm.nih.gov/pubmed/19505943

55. Kimura M. A simple method for estimating evolutionary rates of base substitutions through comparative studies of nucleotide sequences. J. Mol. Evol. [Internet]. 1980 [cited 2016 Aug 9];16:111–20. Available from: http://www.ncbi.nlm.nih.gov/pubmed/7463489

56. Sanmiguel P, Bennetzen JL. Evidence that a Recent Increase in Maize Genome Size was Caused by the Massive Amplification of Intergene Retrotransposons. Ann. Bot. Oxford University Press; 1998;82:37–44.

57. Ma J, Bennetzen JL. Rapid recent growth and divergence of rice nuclear genomes. Proc. Natl.

Acad. Sci. U. S. A. [Internet]. National Academy of Sciences; 2004 [cited 2016 Aug 9];101:12404–10. Available from: http://www.ncbi.nlm.nih.gov/pubmed/15240870

FIGURE LEGENDS AND TABLES

Figure 1. Comparison of *Fragaria vesca* and *Potentilla micrantha* morphology for leaves, flowers and fruits.

Figure 2a. Anchoring of five *Potentilla micrantha* genome scaffolds to the *Fragaria vesca* Fvb pseudomolecules *Fvb*2 and *Fvb*4 demonstrating the microsynteny between the *F. vesca* and *P. micrantha* genomes (numbers in parentheses below the scaffold names indicate the number of genes contained in each split syntenic block.

Figure 2b. A comparison of the seven pseudomolecules of the *F. vesca* genome with eight *P. micrantha* sequencing scaffolds, highlighting the major translocation events identified between the two species in this investigation.

Figure 3. *Potentilla micrantha* flower/fruit developmental stages used for RNA extraction.

Figure 4. Differentially expressed genes during fruit development in *P. micrantha* and *F. vesca.* Volcano plots of differential expression analysis between the four developmental stages A-B-C-D in *Potentilla micrantha* and *Fragaria vesca*. Using a cut-off of sqrt (MSR) > 2.00 and p-value < 10^{-3}, 1,556 genes were differentially expressed in *Potentilla micrantha*, whilst 816 genes were differentially expressed in *Fragaria vesca*.

Figure 5. Over-represented GO-slim categories in *Fragaria vesca* and *Potentilla micrantha* DEGs sets. The circles are shaded based on significance level (yellow = FDR below 0.05), and the radius of each circle is proportional to the number of genes included in each GO-slim category.

Figure 6. Heatmap comparing the log expression values of 205 genes (orthologs of both *F.vesca* and *P.micrantha*) The rows (genes) were sorted using hierarchical clustering using 'correlation' distance and 'complete' linkage. A-D correspond to the four developmental stages defined in the methods section.

Figure 7. A Maximum Likelihood-based phylogenetic reconstruction of the *Potentilla micrantha* and *Fragaria vesca* genes containing MADs-box motifs, along with the relative gene expression levels for each gene. Categories A-D refer to the developmental stages defined in the methods. Filled circles represent the relative level of support for each relationship defined in the Maximum Likelihood analysis.

Figure 8. The three identified clades of orthologous MADS-box motif containing genes that were not expressed or poorly expressed in *Potentilla micrantha* but highly expressed in *Fragaria vesca.* Categories A-D refer to the four developmental stages definedin the methods.

Table 1. *Potentilla micrantha* assembly stats

	ALLPATHS-LG Illumina data	PacBio PBJelly
Number of scaffolds	2,866	2,674 (-6.7%)
Total size of scaffolds	315,266,043	326,533,584 (+3.5%)
Longest scaffold	3,162,838	3,488,351 (+9.3%)
N50 scaffold length	318,490	335,712 (+5.1%)
Gapped Ns in scaffolds	67,706,454	27,311,787 (-59.7%)
Number of contigs	33,026	n/a
Number of contigs in scaffolds	32,063	n/a
Total size of contigs	247,565,733	n/a
N50 contig length	16,235	n/a

Table 2. Annotation of 505 full-length LTR-retrotransposons of *Potentilla micrantha*.

**Superfamily**	**Family**	**Number**	**Percentage**
Ty1-*Copia*	*AleI/Retrofit*	14	2.77
	AleII	26	5.15

	Angela	20	3.96
	Bianca	114	22.57
	Ivana	23	4.55
	Maximus/SIRE	10	1.98
	TAR/Tork	11	2.18
	Unknown	2	0.40
	Total	220	43.56
Ty3-*Gypsy*	*Athila*	3	0.59
	Chromovirus	42	8.32
	Ogre/TAT	186	36.83
	Unknown	25	4.95
	Total	256	50.69
Unclassified		29	5.74

Figure 1 Click here to download Figure Figure 1.tif

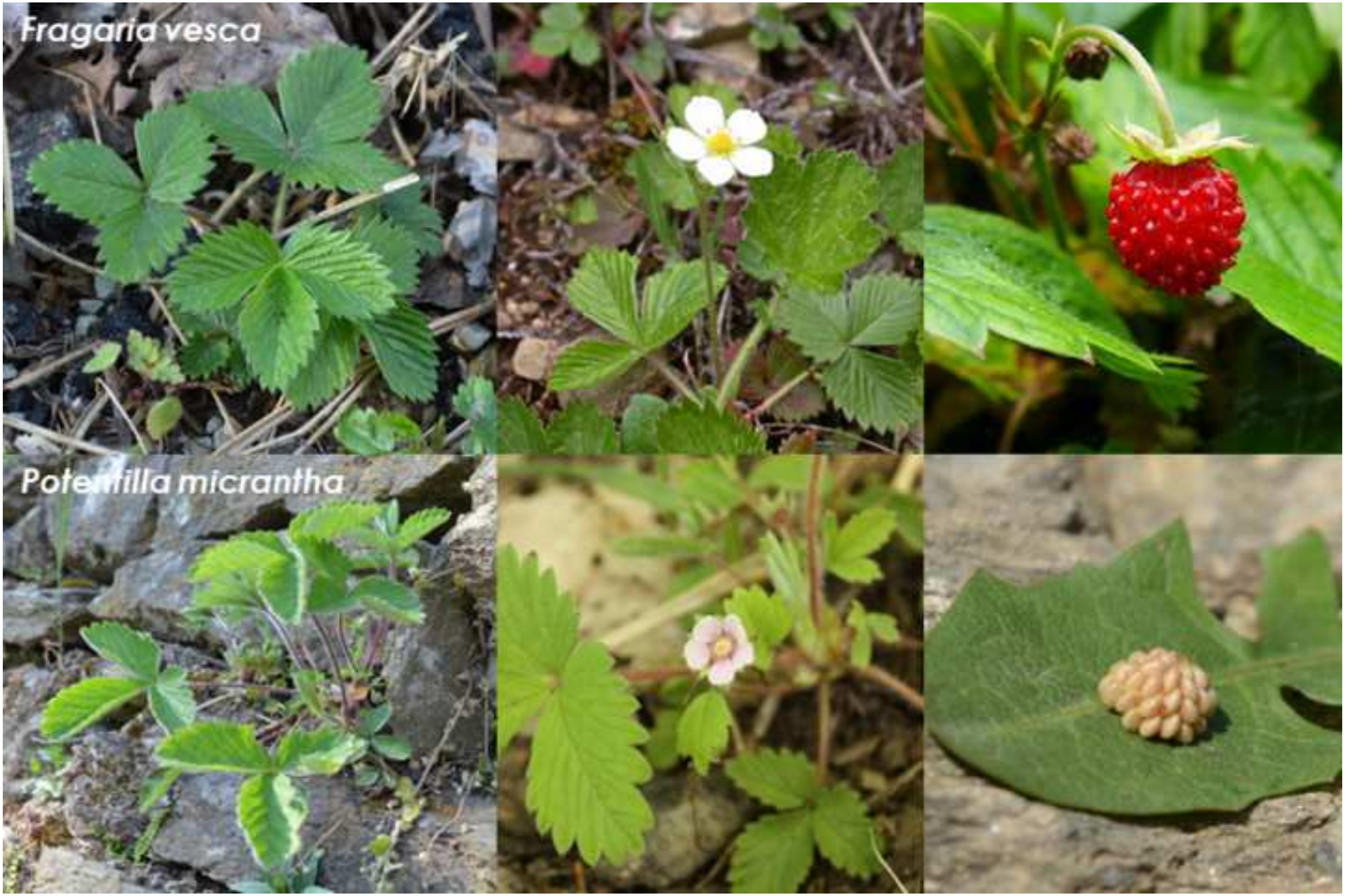

Figure 2a

Click here to download Figure Figure 2a.png

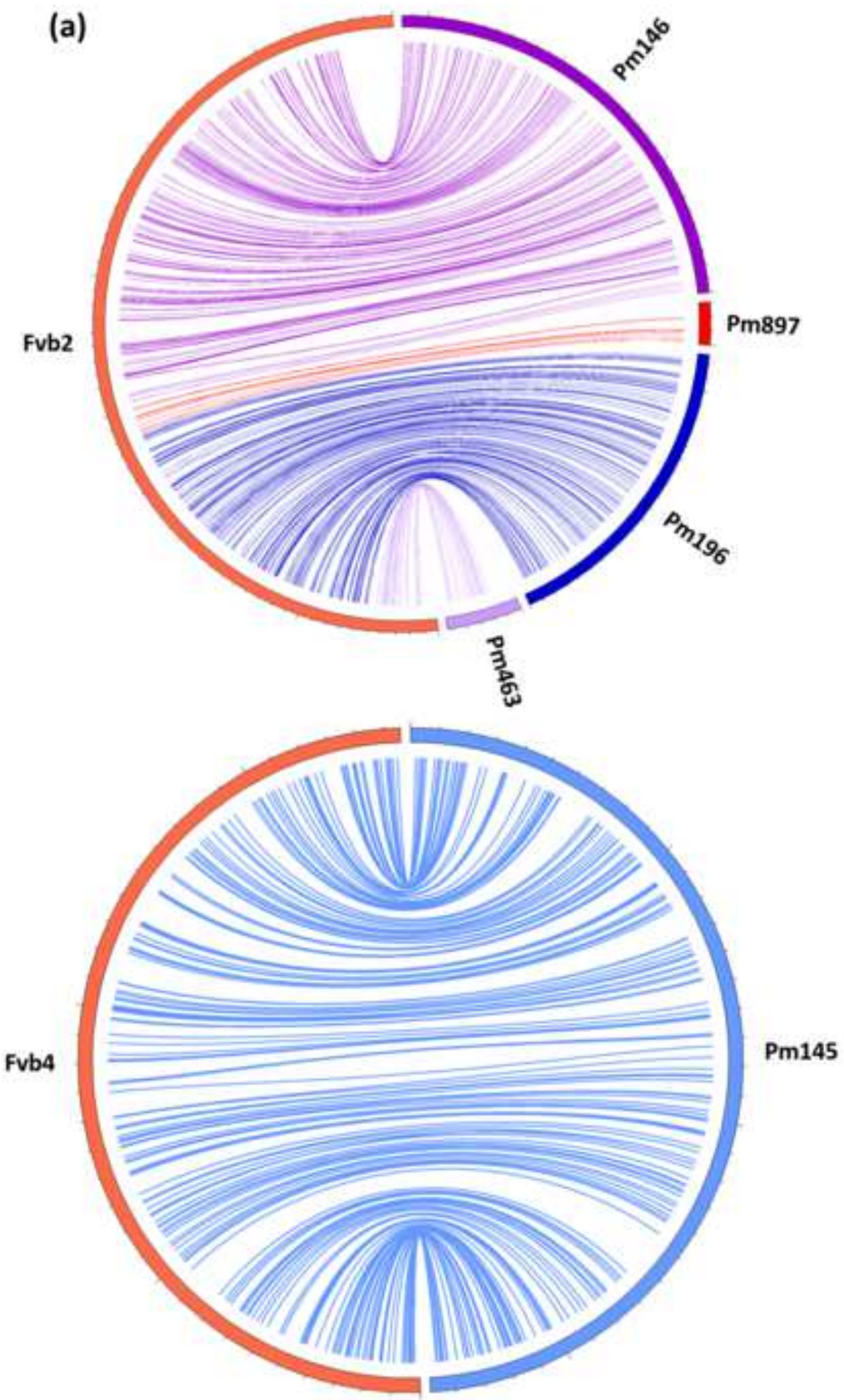

Figure 2b

Click here to download Figure Figure 2b.png

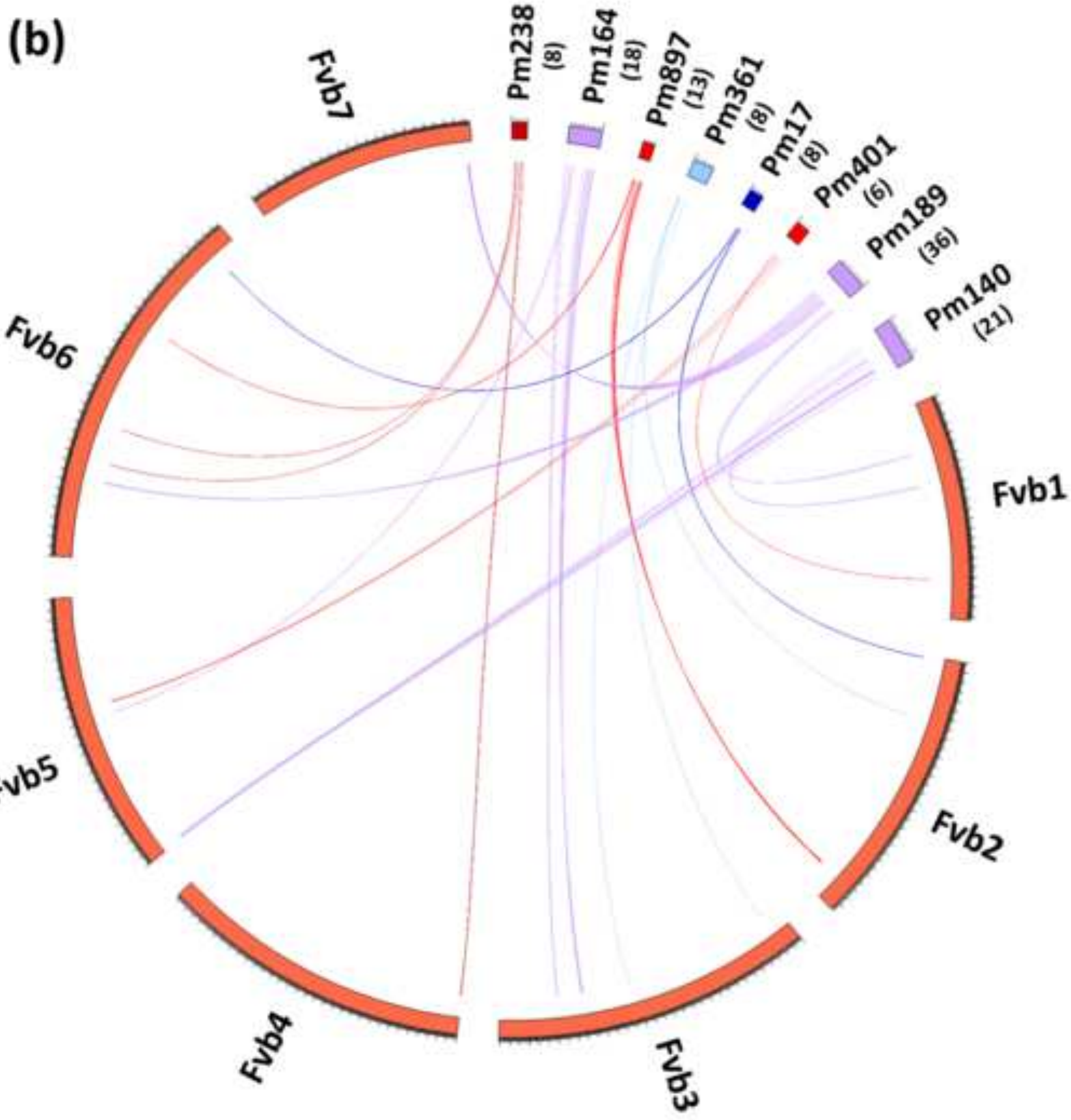

Figure 3

Click here to download Figure Figure 3.tif

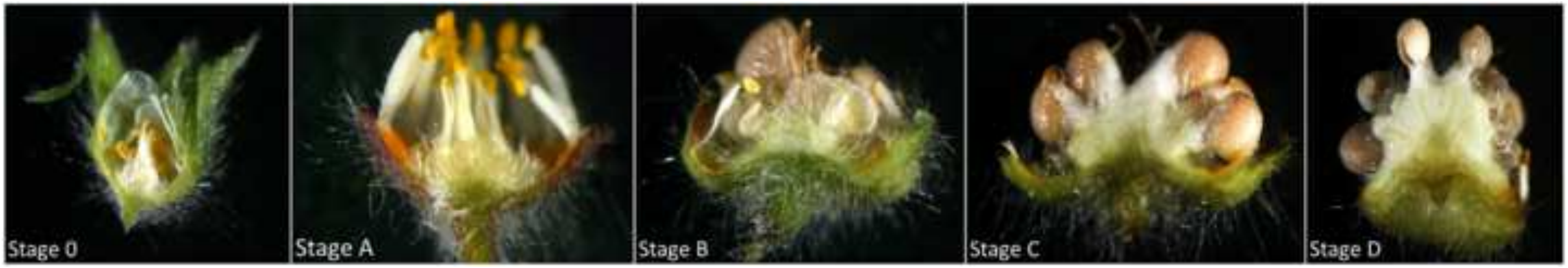

Figure 4

Click here to download Figure Figure 4.tif

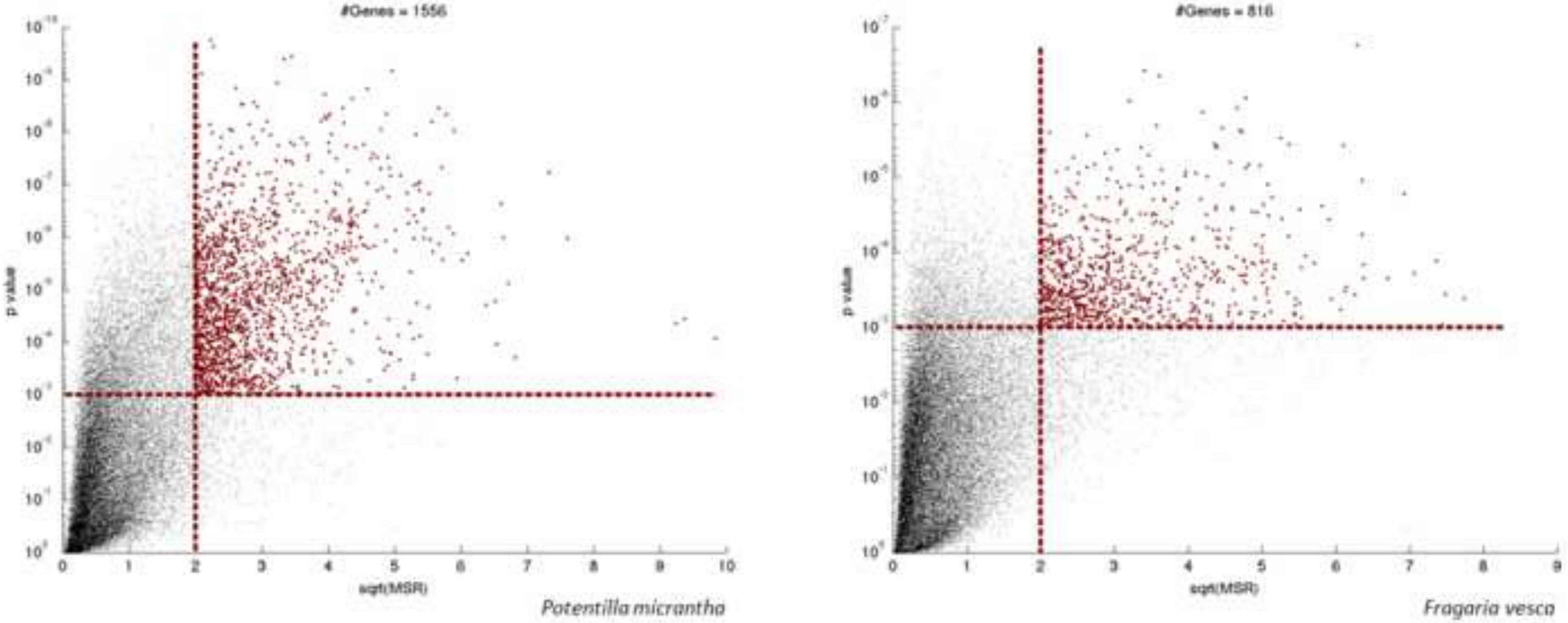

Figure 5

Click here to download Figure Figure 5.tif

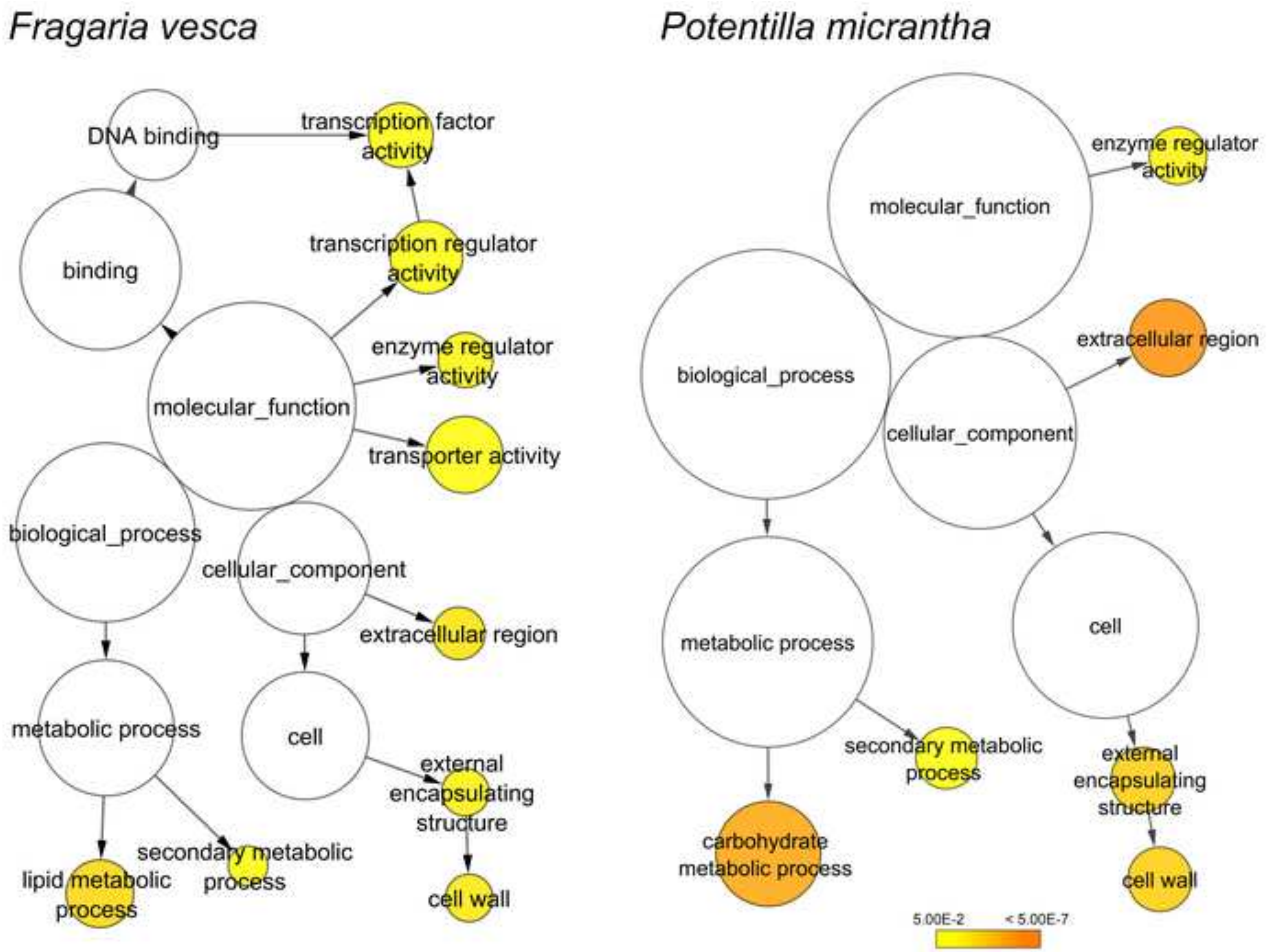

Figure 6

Click here to download Figure Figure6.pdf

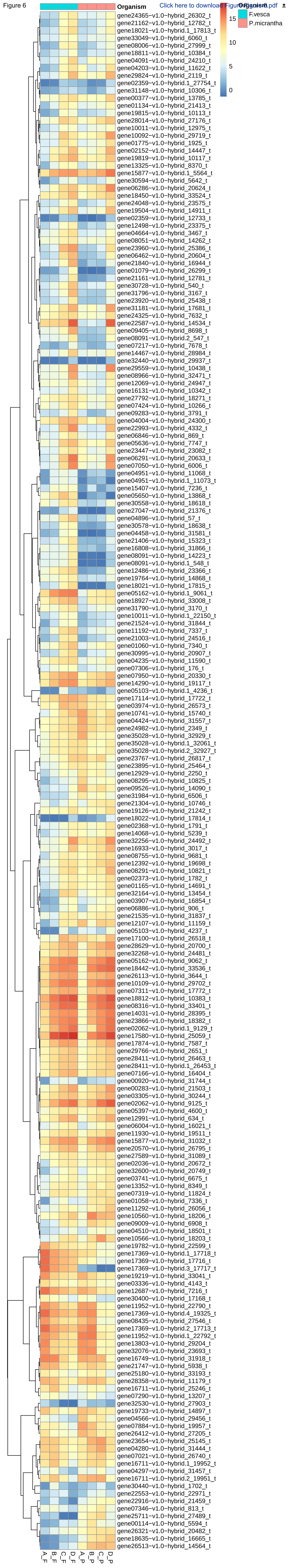

Figure 7 Click here to download Figure Figure 7.tif

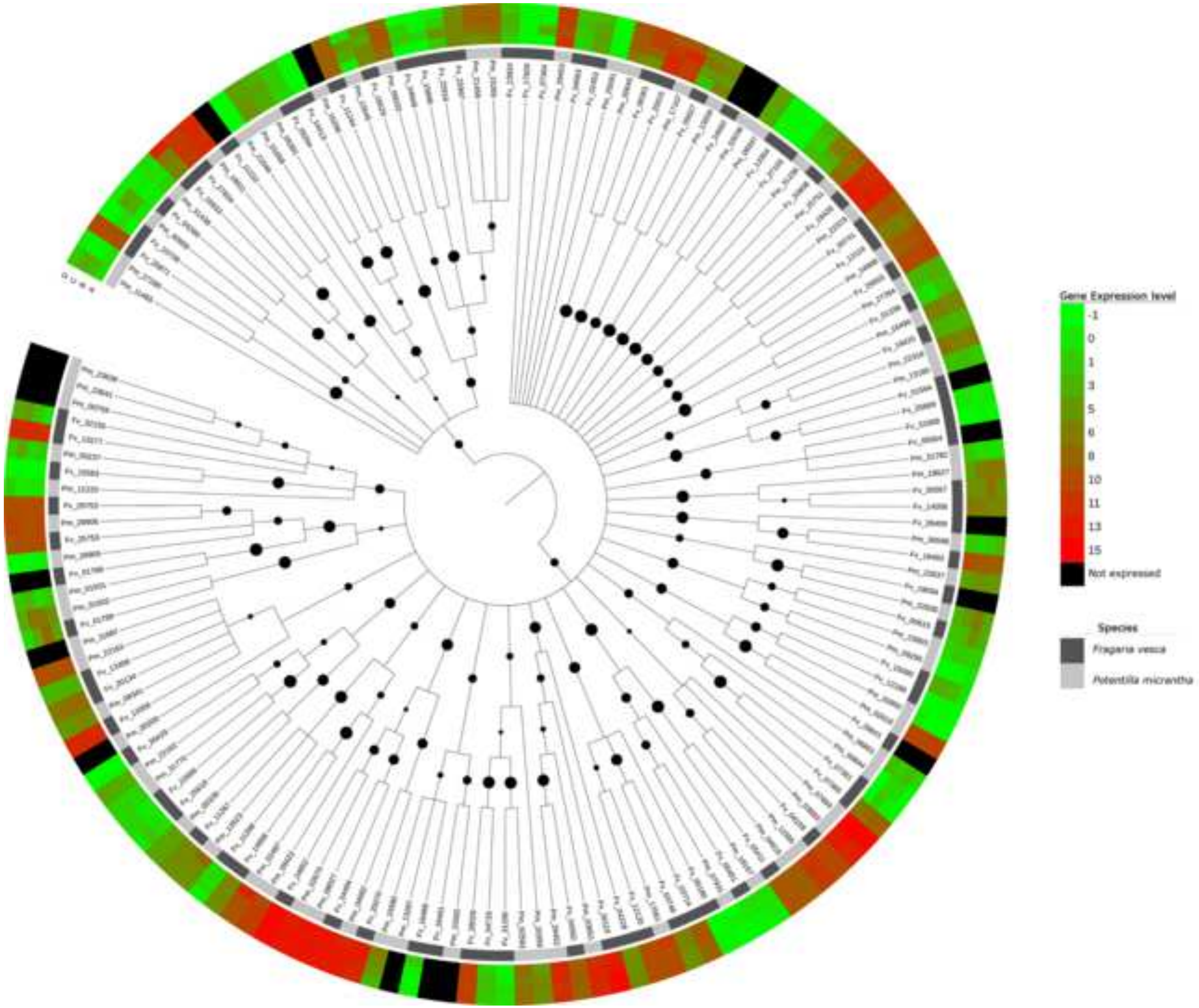

 Click here to download Figure Figure 8.tif

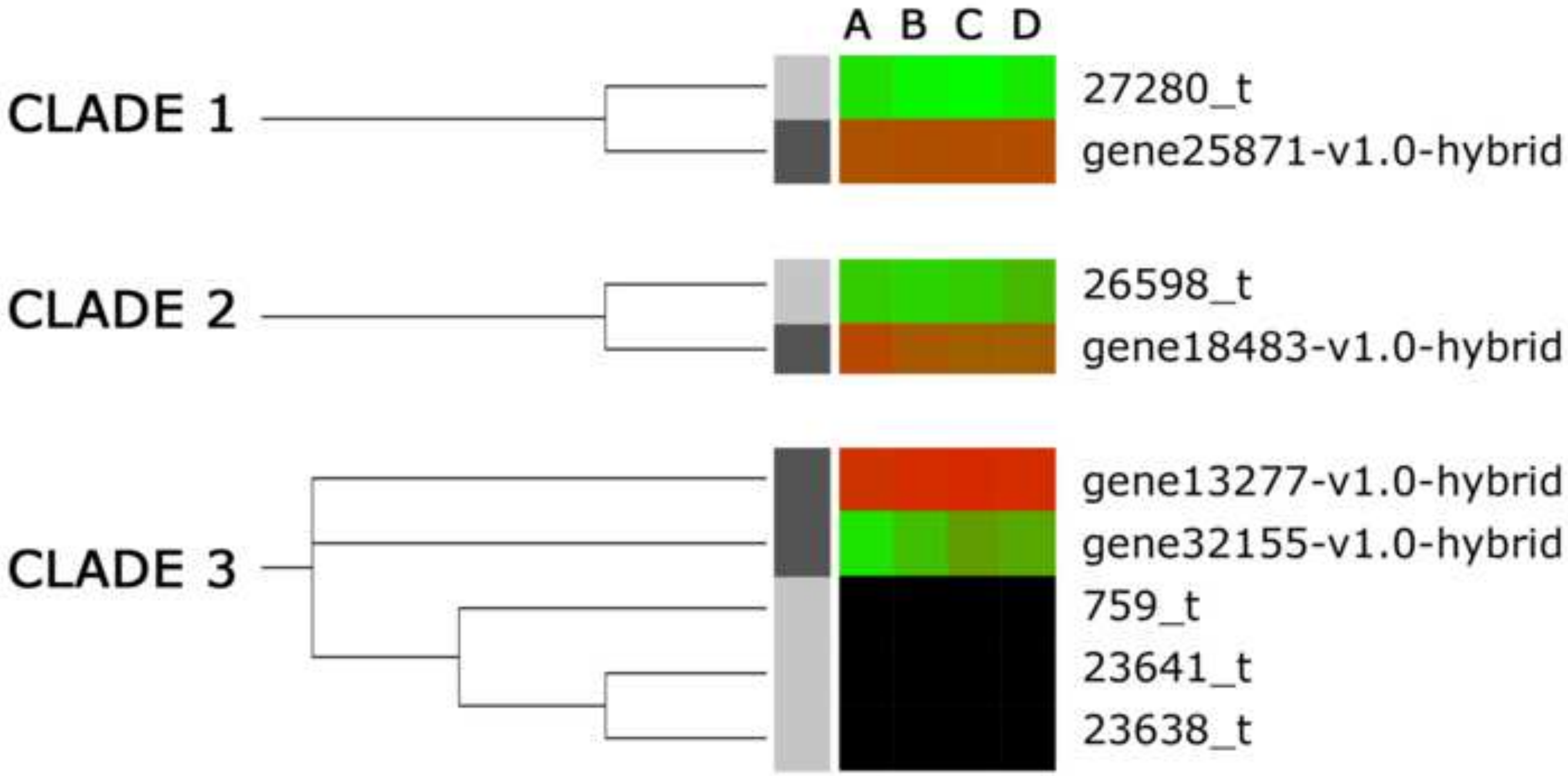

Supplementary Material 1

Click here to access/download
Supplementary Material
Additional_File_1_Table S1.docx

Click here to access/download
Supplementary Material
Additional_File_2_Table S2.docx

Click here to access/download
Supplementary Material
Additional_File_3_Table S3.docx

Click here to access/download
Supplementary Material
Additional_File_4_Table_S4.docx

Click here to access/download
Supplementary Material
Additional_File_5_Figure S1.docx

Supplementary Material 6

Click here to access/download
Supplementary Material
Additional_File_6_Figure_S2.png

Supplementary Material 7

Click here to access/download
Supplementary Material
Additional_File_7_Figure_S3.tif

Click here to access/download
Supplementary Material
Additional_File_8_Figure_S4.docx
