# Supplementary material for: The genome sequence and transcriptome of Potentilla micrantha and their comparison to Fragaria vesca (the woodland strawberry)
Source: Gigascience. 2017 Feb 15;7(4):giy010. doi: 10.1093/gigascience/giy010 (PMC5893959; doi:10.1093/gigascience/giy010)
Supplement: Additional Files [file giy010_supp.zip › Additional_File_1_Table S1.docx]

**Table S1.** Illumina sequencing libraries used in the sequencing of the *Potentilla micrantha* genome including fragment sizes and total genome depth of coverage.

| **Library type** | **Fragment size (bp)** | **Coverage** |
| --- | --- | --- |
| Illumina paired-end | 170 | 57.7× |
| Illumina mate-pair | 3 | 42× |
| Illumina mate-pair | 5 | 20.5× |
| Illumina mate-pair | 8 | 18.3× |
| Illumina mate-pair | 12 | 12.8× |
