# Supplementary material for: The genome sequence and transcriptome of Potentilla micrantha and their comparison to Fragaria vesca (the woodland strawberry)
Source: Gigascience. 2017 Feb 15;7(4):giy010. doi: 10.1093/gigascience/giy010 (PMC5893959; doi:10.1093/gigascience/giy010)
Supplement: Additional Files [file giy010_supp.zip › Additional_File_2_Table S2.docx]

**Table S2.** PacBio RS sequencing kits and chemistries used for *Potentilla micrantha* sequencing.

| **Library** | **PacBio** | **No. SMRT cells** | **No. sequences** | **Total length (Gb)** | **Longest read (kb)** | **N50 read length (bp)** | **Mean read length (bp)** |
| --- | --- | --- | --- | --- | --- | --- | --- |
| PMA001 | P4-C2 | 26 | 2,103,945 | 4.13 | 21.34 | 4,516 | 1,961 |
| PMA002 | P4-C2 | 14 | 95,631 | 1.59 | 21.31 | 2,106 | 1,666 |
| PMA003 | P5-C3 | 7 | 453,338 | 1.64 | 31.3 | 641 | 3,611 |
| PMA004 | P5-C3 | 16 | 2,933,820 | 6.96 | 35.36 | 3,007 | 2,373 |
| **Total** |  | **63** | **6,447,413** | **14.32** | **35.36** | **3,199** | **2,221** |
