# Supplementary material for: The genome sequence and transcriptome of Potentilla micrantha and their comparison to Fragaria vesca (the woodland strawberry)
Source: Gigascience. 2017 Feb 15;7(4):giy010. doi: 10.1093/gigascience/giy010 (PMC5893959; doi:10.1093/gigascience/giy010)
Supplement: Additional Files [file giy010_supp.zip › Additional_File_3_Table S3.docx]

**Table S3.** RNAseq read data used for gene prediction and number of splice sites identified in the *Potentilla micrantha* genome.

| **Parameter** | **Total/average over 12 RNAseq sets** |
| --- | --- |
| Number of input reads | 626,896,967 |
| Average input read length | 202 (2x101) |
| No. uniquely mapped reads | 592,230,354 |
| % uniquely mapped reads | 94.45% |
| Average mapped length | 200.27 |
| **Splice sites** | **Total over 12 RNAseq sets** |
| GT-AG | 350,296 |
| GC-AG | 4,157 |
| AT-AC | 441 |
| Other | 3,391 |
| **Total** | **358,285** |
