# Supplementary material for: The genome sequence and transcriptome of Potentilla micrantha and their comparison to Fragaria vesca (the woodland strawberry)
Source: Gigascience. 2017 Feb 15;7(4):giy010. doi: 10.1093/gigascience/giy010 (PMC5893959; doi:10.1093/gigascience/giy010)
Supplement: Additional Files [file giy010_supp.zip › Additional_File_4_Table_S4.docx]

**Table S4.** *Potentilla micrantha* and *Fragaria vesca* RNAseq reads statistics.

|  | **library** | **# initial reads** | **# reads after trimming** | **% reads after trim** | **Aln. rate to transcriptome** |
| --- | --- | --- | --- | --- | --- |
| ***Potentilla micrantha*** | **0** | 30,293,264 | 29,653,058 | 97.89% | 65.13% |
|  | **A1** | 47,582,182 | 46,430,978 | 97.58% | 66.83% |
|  | **A2** | 53,215,453 | 51,908,946 | 97.54% | 68.41% |
|  | **B1** | 65,539,518 | 63,944,349 | 97.57% | 64.41% |
|  | **B2** | 55,158,599 | 53,486,293 | 96.97% | 70.03% |
|  | **C1** | 38,721,137 | 37,909,403 | 97.90% | 62.89% |
|  | **C2** | 60,012,349 | 58,459,990 | 97.41% | 68.63% |
|  | **C3** | 61,688,704 | 60,158,302 | 97.52% | 68.55% |
|  | **D1** | 54,357,203 | 53,196,616 | 97.86% | 68.29% |
|  | **D3** | 57,946,398 | 56,421,786 | 97.37% | 69.10% |
|  | **D4** | 54,186,721 | 52,899,580 | 97.62% | 69.17% |
|  | **YL** | 55,885,739 | 54,615,814 | 97.73% | 69.03% |
|  | **TOTAL** | **634,587,267** | **619,085,115** | **97.76%** | **67.73%** |
| ***Fragaria vesca*** | **A1** | 110,886,356 | 110,347,314 | 99.51% | 63.84% |
|  | **A2** | 110,108,195 | 109,643,224 | 99.58% | 63.70% |
|  | **B1** | 116,852,713 | 115,170,350 | 98.56% | 64.41% |
|  | **B2** | 132,864,883 | 131,623,001 | 99.07% | 66.55% |
|  | **C1** | 114,173,482 | 111,788,559 | 97.91% | 62.78% |
|  | **C2** | 114,253,904 | 111,890,338 | 97.93% | 64.39% |
|  | **D1** | 116,383,562 | 114,091,012 | 98.03% | 62.60% |
|  | **D2** | 158,056,143 | 155,643,061 | 98.47% | 66.16% |
|  | **E1** | 128,818,961 | 126,638,949 | 98.31% | 67.58% |
|  | **E2** | 151,978,989 | 150,046,732 | 98.73% | 67.61% |
|  | ***TOTAL*** | ***1,254,377,188*** | ***1,236,882,540*** | ***98.60%*** | ***65.14%*** |
