# Supplementary material for: The genome sequence and transcriptome of Potentilla micrantha and their comparison to Fragaria vesca (the woodland strawberry)
Source: Gigascience. 2017 Feb 15;7(4):giy010. doi: 10.1093/gigascience/giy010 (PMC5893959; doi:10.1093/gigascience/giy010)
Supplement: Additional Files [file giy010_supp.zip › Additional_File_5_Figure S1.docx]

**Figure S2.** Distribution of predicted genes *Potentilla micrantha* and *Fragaria vesca* mapped, blasted and GO-annotated by BLAST2GO analysis.
