# Supplementary material for: The genome sequence and transcriptome of Potentilla micrantha and their comparison to Fragaria vesca (the woodland strawberry)
Source: Gigascience. 2017 Feb 15;7(4):giy010. doi: 10.1093/gigascience/giy010 (PMC5893959; doi:10.1093/gigascience/giy010)

**Fig S4.** Genome proportion in *Potentilla micrantha* and *Fragaria vesca* of 291 repeats clustered using RepeatExplorer. Other repeats include satellite DNAs, pararetroviruses, and one LINE.


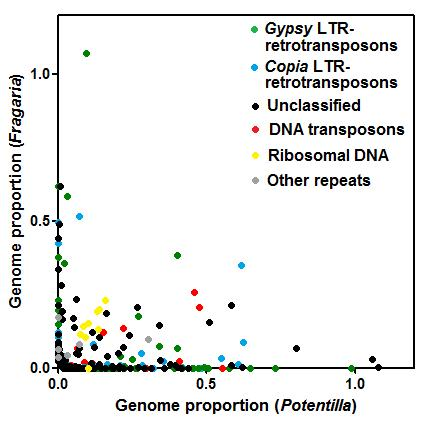

Supplement: Additional Files [file giy010_supp.zip › Additional_File_8_Figure_S4.docx]
