# Supplementary figures and images for: The genome sequence and transcriptome of Potentilla micrantha and their comparison to Fragaria vesca (the woodland strawberry)
Source: Gigascience. 2017 Feb 15;7(4):giy010. doi: 10.1093/gigascience/giy010 (PMC5893959; doi:10.1093/gigascience/giy010)

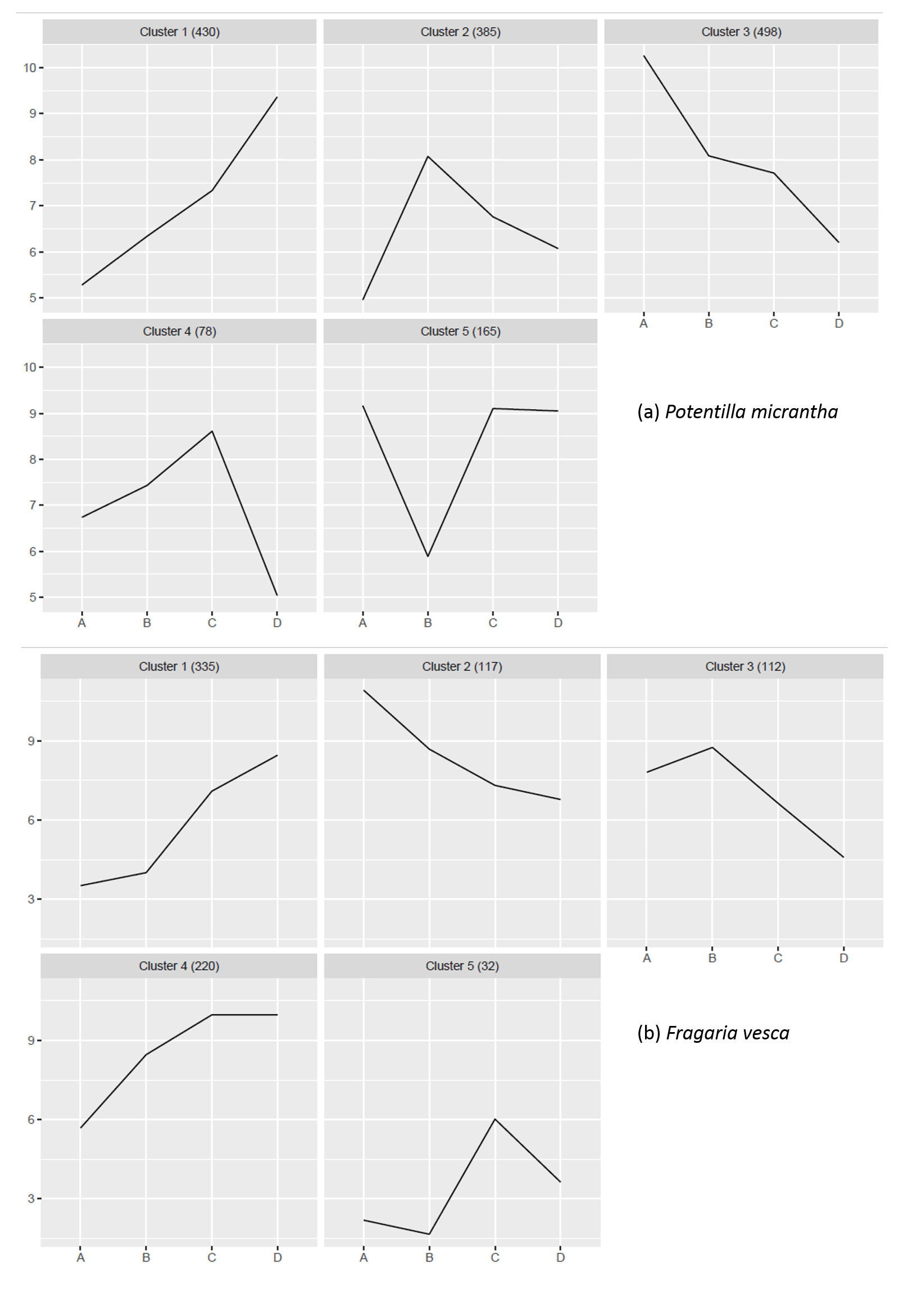

Supplement: Additional Files [file giy010_supp.zip › Additional_File_6_Figure_S2.png]

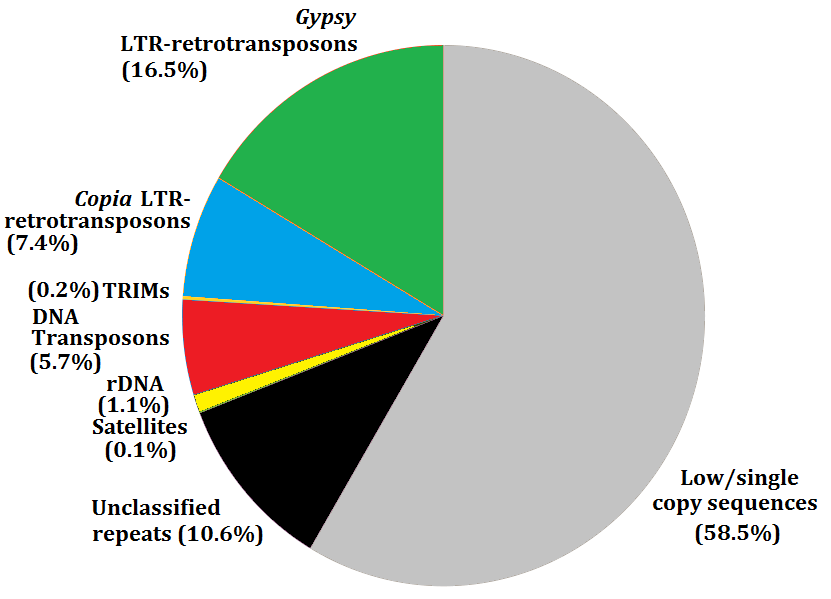

Supplement: Additional Files [file giy010_supp.zip › Additional_File_7_Figure_S3.tif]
