# Supplementary material for: The genome sequence and transcriptome of Potentilla micrantha and their comparison to Fragaria vesca (the woodland strawberry)
Source: Gigascience. 2017 Feb 15;7(4):giy010. doi: 10.1093/gigascience/giy010 (PMC5893959; doi:10.1093/gigascience/giy010)
Supplement: Response_to_Reviewer_Comments_Original_Submission.pdf [file giy010_response_to_reviewer_comments_original_submission.pdf]

Author's Response To Reviewer Comments

Dr Daniel James Sargent
Driscoll's Genetics Limited
East Malling Enterprise Centre
New Road
East Malling
Kent, ME19 6BJ, UK
3rd November 2017

Dear Editor,
Please find attached the revision of our original article. Below please find a point-by-point description of the changes made in the light of the reviewers' comments. We would like to thank both you and the reviewers, as we feel the changes that have been made have significantly enhanced and strengthened the paper.

Reviewer 1
We have tones down the whole of the manuscript to reflect the descriptive nature of our data and have likewise changed the title of the paper to: The genome sequence and transcriptome of Potentilla micrantha and their comparison to Fragaria vesca (the woodland strawberry).
The figure legends have been checked and corrected where necessary.
The figure relating to anchoring of scaffolds has been moved to the supplementary material and replaced with figures relating to synteny of specific scaffolds rather than the genome as a whole. Additionally, we have ensured throughout the text that it is clear that only micro-synteny was evaluated.
A BUSCO analysis has been performed and presented.
An analysis showing the overlap between the DEGs in each species was performed, as well as a visualisation of the genes from each species and the GO class they fall into.
The Transposon analysis section has been reduced.
The hormonal treatment analysis has been removed from the paper.
The miR1511 data has been removed from the paper as further work would have been required to strengthen this section sufficiently for publication which was not possible since almost all authors now no longer work at FEM where this work was initiated.

Reviewer 2
We appreciate the comments regarding the mechanisms of differentiation, and indeed at the inception of the project this was to be a major focus of the work; however, we were not able to progress in this area sufficiently to make this a major part of the manuscript. We hope that other groups will be able to study this area, building on the work we present here.
We have added a space between x and ananassa.
We have removed the redundancy and made clearer the objectives of the study.
Figure numbering has been corrected.
The ML study is presented the others have been referred to as data not shown.
Plants were selected from Serbia as we had a collaborator there who guided us to a large population from which we could sample plant material easily.
Redundancy has been removed from the HiSeq2000 methods section.

We have adjusted the text relating to FPKM to clarify that highly expressed genes were those with FPKM >1000 and on/off genes were those where no expression data were observed.
A space was added to sqrt (MSR).
Abbreviations have been added for ML, MP and NJ in the text.
Resolution of the figures has been improved and font size increased to improve clarity.
Figure legends for the phylogenetic analysis have been improved. The text resolution on the submitted figures is much better than in the reviewer copy. We hope that in the revised version, the reviewers have access to higher resolution images where text is hopefully clear and legible.

Reviewer 3
The text has been modified throughout to make clearer that only micro-synteny was evaluated. Likewise, the figures relating to this section have been changed to reflect and emphasise the micro-synteny.
The abundance of GO terms for the DEGs in each species has been highlighted through an additional figure, and those classes that were in greater abundance are identified. Likewise, a heatmap of the expression levels of genes shared between the two species has been produced and those that differ in their expression patterns have been identified.
The title and text have been toned down to reflect the results presented more accurately.

We look forward to hearing from you in due course regarding this resubmission,
Best regards,
Dan Sargent (on behalf of all authors).
