# Supplementary material for: The genome sequence and transcriptome of Potentilla micrantha and their comparison to Fragaria vesca (the woodland strawberry)
Source: Gigascience. 2017 Feb 15;7(4):giy010. doi: 10.1093/gigascience/giy010 (PMC5893959; doi:10.1093/gigascience/giy010)
Supplement: Response_to_Reviewer_Comments_Revision_1.pdf [file giy010_response_to_reviewer_comments_revision_1.pdf]

Author's Response To Reviewer Comments

Dr Daniel James Sargent
Driscoll's Genetics Limited
East Malling Enterprise Centre
New Road
East Malling
Kent, ME19 6BJ, UK
4th January 2018

Dear Dr Zauner,

Please find enclosed our revised manuscript: GIGA-D-17-00155R2 The genome sequence and transcriptome of Potentilla micrantha and their comparison to Fragaria vesca (the woodland strawberry), which has been reformatted in the style of a Data Note.

Below we provide a point-by-point description of the changes made in response to the reviewers comments and hope that the changes we have made will be sufficient for the paper to be acceptable for publication.

We look forward to hearing from you in due course regarding our revised paper.

Very best regards,

Dan Sargent (on behalf of all authors).

Responses to Reviewer's comments:

Q1. Overall, I am pleased that the reviewers agree that the latest, revised version of your manuscript presents useful and valuable data. However, I also agree with reviewer 1 that, in the absence of more in-depth analyses, the main value of the paper is now indeed the presentation of a high-quality resource, rather than new biological insights.

R1. Many thanks for the useful comments. We agree with your feeling that the paper describes more of a technical advance than a description of new biological insights at this stage, but hope we have provided a valuable dataset that can be used for future biological investigations.

Q2. I therefore feel that your revised manuscript may be more suitable for the format of a "Data Note". Data notes are indexed in the same way as research articles, but the emphasis is on presenting an exceptional resource, together with description and validation of the dataset. If you agree that we consider your next revised version as a data note, please adjust the format (see: https://academic.oup.com/gigascience/pages/data_note). I feel that this would require only minor adjustments to the text itself (e.g. the discussion part could be revised to demonstrate validation and re-use cases of the data). You can keep the Background section for the Data Note format (this is not quite clear from our Instructions).

Please let me know if you agree to this suggestion, and I'm happy to answer any questions regarding the Data Note format.

R2. We are happy for our paper to be published as a data note and have revised the manuscript accordingly (following the guidelines and taking recently published data note articles as examples).

Reviewer reports:
Q3. Reviewer #1: The authors addressed some of the main issues appropriately (synteny, figures etc.). However, for other issues like the transposon section, the hormonal treatment analysis and the miR1511 analysis the main action was just to shorten or completely drop the part. This is Ok and/or was suggested but on the other hand, no real efforts were made to strengthen the comparison/fleshy fruit analyses (or any other analytical part) and most of my suggestions/questions for this and also the annotation and gene expression part were simply ignored. As a result, this study as it stands now mostly provides an "extended description" of the resources generated (although potentially valuable) with clear shortcomings in the analysis and interpretation of the data. Along that lines, I appreciate that the authors in the new version resign from claiming analytical results not there or possible.

R3. We appreciate the reviewer's useful and constructive criticisms on the previous version of our paper and whilst we tried to incorporate as many of the suggested changes as was possible, the lead authors did not have the resources available to make all the suggested improvements, and as such we opted to remove the unsupported claims and refocus the paper as a technical report.

Additional comments:
Q4. a.) Your BUSCO analysis shows that you are missing ~6% of the BUSCO genes from the genome sequence (present) to the final gene prediction (absent). Are they completely missing in the gene prediction or fragmented etc.?

R4. We have provided more detail in the manuscript, detailing the complete and fragmented BUSCO sequences retrieved. We have also performed a BUSCO analysis on the F. vesca gene predictions and provided a comparison of the two to demonstrate that the P. micrantha set is virtually as complete as the F. vesca set.

Q5. b.) I still wonder about the ~9,000 gene predictions not showing a hit on the F. vesca pseudomolecules…do those genes have functional annotation and expression support?

R5. Our sincere apologies for not making this section clearer (it was not explained carefully enough what analyses we are performing and the results were not reported in detail). The Inparanoid analysis attempted to identify orthologous genes between the P. micrantha and F. vesca datasets using protein sequences. This analysis revealed 33,127 P. micrantha genes that had a putative match in the F. vesca gene set (98.6%). However, for the analysis of synteny, we chose only those genes where there was a clear and unambiguous orthologous relationship between the two genomes. This is the reason only 24,555 genes were considered for the synteny analysis. We have amended the text to clarify these points.

Q6. c.) I cannot make much sense out of figure 2B. In the figure resolution I have, individual lines look like they correspond to a single (or very few) gene(s), although I suspect it has to be more genes. It would be good to define the sizes of the blocks somewhere.

R6. We have added the number of genes in each conserved region which are split between pseudomolecules on the F. vesca genome. We have also made it clearer that these are only scaffolds that are split between pseudomolecules (i.e. evidence of major rearrangements). There were many instances of individual genes that did not fall into syntenic blocks.

Q7. d.) I'd move figure 9 to the supplementary material and drop sup Figure S1. It still has the same problem as when it was a main figure.

R7. Done as suggested

Reviewer #3: The authors have addressed all my comments and concerns.
Q8. I have one minor comment -I found 'CDs' in multiple places. Could you spell it out when it first appears? If it stands for coding DNA sequence, I think 'CDS' is a more common abbreviation.

R8. Done as suggested.
