# Supplementary material for: The genome sequence and transcriptome of Potentilla micrantha and their comparison to Fragaria vesca (the woodland strawberry)
Source: Gigascience. 2017 Feb 15;7(4):giy010. doi: 10.1093/gigascience/giy010 (PMC5893959; doi:10.1093/gigascience/giy010)
Supplement: Response_to_Reviewer_Comments_Revision_2.pdf [file giy010_response_to_reviewer_comments_revision_2.pdf]

Author's Response To Reviewer Comments

Dear Dr Zauner, please find attached our manuscript with the minor modifications made as you suggested. Many thanks for guiding our manuscript through the review process, it is greatly appreciated. Best regards, Dan Sargent
