# Supplementary material for: The genome sequence and transcriptome of Potentilla micrantha and their comparison to Fragaria vesca (the woodland strawberry)
Source: Gigascience. 2017 Feb 15;7(4):giy010. doi: 10.1093/gigascience/giy010 (PMC5893959; doi:10.1093/gigascience/giy010)
Supplement: Reviewer_1_Report_(Original_Submission) -- Manuel Spannagl — 01 Aug 2017 Reviewed [file giy010_reviewer_1_report_(original_submission).pdf]

Reviewer Report

Title: The genome sequence and transcriptome of Potentilla micrantha and their comparison to Fragaria vesca (the woodland strawberry)

Version: Original Submission **Date:** 01 Aug 2017

Reviewer name: Manuel Spannagl

Reviewer Comments to Author:

In this manuscript the authors report the genome sequencing, annotation and analysis of P. micrantha and compare transcriptome&genome data to the related Fragaria clade to investigate fleshy fruit development. I agree with the authors that this new resource represents a valuable tool to further and in-depth study fruit development and evolution in this clade and beyond. The analysis of exactly this, however, remains superficial in this study albeit the title promises more…this is my main point of criticism. The identification of the three differentially expressed orthologous clades seems sound from the methodology point of view but lacks any further evaluation or analysis. What about the promotor regions of these orthologs? Any explanation for the different expression profiles? I understand this is a genome and comparative genomics study but it would be nice to see some additional (e.g. experimental) evidence for the functional role of those genes except of their sequence homology to Arabidopsis. Also, do you find any additional (differentially expressed or not) genes potentially related to fruit development (e.g. known genes from Fragaria)? Until this is not included, you should at least tone down the titel's promises…

Additional points to consider:

a.) Figure numbering is incorrect between legends and actual figures.

b.) Figure 3 (anchoring…fig 2 in the legend): I understand that this figure represents the anchoring of the scaffolds on the pseudochromosomes of Fragaria but I also feel that this figure is somewhat suggestive of an aspect that the authors cannot really assess. As the scaffolds are not ordered or oriented along a map (see pg. 18…and no attempts were made either according to authors) the authors can assess the micro-synteny but not the macro-synteny (this is also clearly mentioned in the methods). The ordering along the Fragaria pseudochromosomes in Figure 3 together with too general conclusions about syntenic conservation (see chapter "Scaffold anchoring and synteny…" and conclusions pg.12, ln 22) between P. micrantha and Fragaria give the impression about high conservation on the macro-synteny level, too (though possible, but not shown).

c.) Annotation: there is no real evaluation of the gene prediction quality other than BLAST searches and BLAST2GO annotations. It would be nice to see a BUSCO evaluation or even a gene family clustering with related species…this would give a good proxy on gene prediction completeness and also quality. What about the ~9,000 P. micrantha genes not identified on the F. vesca pseudomolecules? Are they all true species-specific genes? Is there expression evidence for them in your data?

d.) Gene expression during fruit development: with 1,556 DEGs in P. micrantha and 816 DEGs in F. vesca I was wondering about the overlap and differences between these two genes sets? This would be much more informative than the GO annotation statistics for these…in that context, I do not understand Fig.

S1. How are the different categories exactly defined and what should be the conclusion of it? I was also wondering about the comparability of the expression data generated in this study and the one by Kang et al. Although they should represent identical tissues etc., is there any statistical and/or technical evaluation available (e.g. PCA etc.)?
e.) Parts of the transposon analysis read extremely descriptive without major conclusions…this could be substantially shortened in my view.
f.) Hormonal treatment: this chapter seems very much isolated…what is the conclusion here and how does it relate to the rest of the analyses?
g.) Is there a reason for not showing the miR1511 data and analysis? This appears to be potentially interesting but cannot be assessed if just mentioned in the discussions…

Methods

Are the methods appropriate to the aims of the study, are they well described, and are necessary controls included? Yes

Conclusions

Are the conclusions adequately supported by the data shown? No

Reporting Standards

Does the manuscript adhere to the journal's guidelines on minimum standards of reporting? Yes

Statistics

Are you able to assess all statistics in the manuscript, including the appropriateness of statistical tests used? There are no statistics in the manuscript.

Quality of Written English

Please indicate the quality of language in the manuscript: Acceptable

Declaration of Competing Interests

Please complete a declaration of competing interests, considering the following questions:

- Have you in the past five years received reimbursements, fees, funding, or salary from an organisation that may in any way gain or lose financially from the publication of this manuscript, either now or in the future?

- Do you hold any stocks or shares in an organisation that may in any way gain or lose financially from the publication of this manuscript, either now or in the future?

- Do you hold or are you currently applying for any patents relating to the content of the manuscript?

- Have you received reimbursements, fees, funding, or salary from an organization that holds or has applied for patents relating to the content of the manuscript?

- Do you have any other financial competing interests?

- Do you have any non-financial competing interests in relation to this paper?

If you can answer no to all of the above, write 'I declare that I have no competing interests' below. If your reply is yes to any, please give details below.

I declare that I have no competing interests

I agree to the open peer review policy of the journal. I understand that my name will be included on my report to the authors and, if the manuscript is accepted for publication, my named report including any attachments I upload will be posted on the website along with the authors' responses. I agree for my report to be made available under an Open Access Creative Commons CC-BY license (http://creativecommons.org/licenses/by/4.0/). I understand that any comments which I do not wish to be included in my named report can be included as confidential comments to the editors, which will not be published.

I agree to the open peer review policy of the journal

To further support our reviewers, we have joined with Publons, where you can gain additional credit to further highlight your hard work (see: https://publons.com/journal/530/gigascience). On publication of this paper, your review will be automatically added to Publons, you can then choose whether or not to claim your Publons credit. I understand this statement.

Yes
