# Supplementary material for: The genome sequence and transcriptome of Potentilla micrantha and their comparison to Fragaria vesca (the woodland strawberry)
Source: Gigascience. 2017 Feb 15;7(4):giy010. doi: 10.1093/gigascience/giy010 (PMC5893959; doi:10.1093/gigascience/giy010)
Supplement: Reviewer_1_Report_(Revision_1) -- Manuel Spannagl — 21 Nov 2017 Reviewed [file giy010_reviewer_1_report_(revision_1).pdf]

Reviewer Report

Title: The genome sequence and transcriptome of Potentilla micrantha and their comparison to Fragaria vesca (the woodland strawberry)

Version: Revision 1 **Date:** 21 Nov 2017

Reviewer name: Manuel Spannagl

Reviewer Comments to Author:

The authors addressed some of the main issues appropriately (synteny, figures etc.). However, for other issues like the transposon section, the hormonal treatment analysis and the miR1511 analysis the main action was just to shorten or completely drop the part. This is Ok and/or was suggested but on the other hand, no real efforts were made to strengthen the comparison/fleshy fruit analyses (or any other analytical part) and most of my suggestions/questions for this and also the annotation and gene expression part were simply ignored. As a result, this study as it stands now mostly provides an "extended description" of the resources generated (although potentially valuable) with clear shortcomings in the analysis and interpretation of the data. Along that lines, I appreciate that the authors in the new version resign from claiming analytical results not there or possible.
Additional comments:
a.) Your BUSCO analysis shows that you are missing ~6% of the BUSCO genes from the genome sequence (present) to the final gene prediction (absent). Are they completely missing in the gene prediction or fragmented etc.?
b.) I still wonder about the ~9,000 gene predictions not showing a hit on the F. vesca pseudomolecules…do those genes have functional annotation and expression support?
c.) I cannot make much sense out of figure 2B. In the figure resolution I have, individual lines look like they correspond to a single (or very few) gene(s), although I suspect it has to be more genes. It would be good to define the sizes of the blocks somewhere.
d.) I'd move figure 9 to the supplementary material and drop sup Figure S1. It still has the same problem as when it was a main figure.

Methods

Are the methods appropriate to the aims of the study, are they well described, and are necessary controls included? Yes

Conclusions

Are the conclusions adequately supported by the data shown? Yes

Reporting Standards

Does the manuscript adhere to the journal's guidelines on minimum standards of reporting? Yes

Statistics

Are you able to assess all statistics in the manuscript, including the appropriateness of statistical tests used? There are no statistics in the manuscript.

Quality of Written English

Please indicate the quality of language in the manuscript: Acceptable

Declaration of Competing Interests

Please complete a declaration of competing interests, considering the following questions:

- Have you in the past five years received reimbursements, fees, funding, or salary from an organisation that may in any way gain or lose financially from the publication of this manuscript, either now or in the future?
- Do you hold any stocks or shares in an organisation that may in any way gain or lose financially from the publication of this manuscript, either now or in the future?
- Do you hold or are you currently applying for any patents relating to the content of the manuscript?
- Have you received reimbursements, fees, funding, or salary from an organization that holds or has applied for patents relating to the content of the manuscript?
- Do you have any other financial competing interests?
- Do you have any non-financial competing interests in relation to this paper?

If you can answer no to all of the above, write 'I declare that I have no competing interests' below. If your reply is yes to any, please give details below.

I declare that I have no competing interests

I agree to the open peer review policy of the journal. I understand that my name will be included on my report to the authors and, if the manuscript is accepted for publication, my named report including any attachments I upload will be posted on the website along with the authors' responses. I agree for my report to be made available under an Open Access Creative Commons CC-BY license (http://creativecommons.org/licenses/by/4.0/). I understand that any comments which I do not wish to be included in my named report can be included as confidential comments to the editors, which will not be published.

I agree to the open peer review policy of the journal

To further support our reviewers, we have joined with Publons, where you can gain additional credit to further highlight your hard work (see: https://publons.com/journal/530/gigascience). On publication of this paper, your review will be automatically added to Publons, you can then choose whether or not to claim your Publons credit. I understand this statement.

Yes
