# Supplementary material for: The genome sequence and transcriptome of Potentilla micrantha and their comparison to Fragaria vesca (the woodland strawberry)
Source: Gigascience. 2017 Feb 15;7(4):giy010. doi: 10.1093/gigascience/giy010 (PMC5893959; doi:10.1093/gigascience/giy010)
Supplement: Reviewer_2_Report_(Original_Submission) -- Soichiro Nagano — 05 Aug 2017 Reviewed [file giy010_reviewer_2_report_(original_submission).pdf]

Reviewer Report

Title: The genome sequence and transcriptome of Potentilla micrantha and their comparison to Fragaria vesca (the woodland strawberry)

Version: Original Submission **Date:** 05 Aug 2017

Reviewer name: Soichiro Nagano

Reviewer Comments to Author:

This study presented the genome of Potentilla micrantha, a closely related species with Fragaria vesca, which is the model species of the cultivated strawberry, F. x ananassa and Rosaceae family, and the authors compared them. It is really interesting about the homology in the genome and morphological characteristics between the two species, and in the suggestion based on the results in comparison in MADs-box conserved domain-containing genes and transposable elements. Therefore, I consider that it is worthwhile to accept this paper in GigaScience. In globaly, Potentilla and Fragaria both "grow within the same ecological niches, and where their ranges of distribution overlap" as the authors presented in the introduction. In relation to the evolutionary context, why the two genus have been able to be separated? Because it is possible that gene flow could suppress adaptive differentiation at the beginning of the speciation if a non-mutated and a mutated individual have existed in the common habitat. And also, Potentilla or Fragaria, which genus is the closest genus that is remaining the characteristics of the common ancestor? If the authors discuss about the mechanisms affected on the differentiation, this study would attract attention from various researcher related with plant sciences. Anyway, the manuscript has been well constructed with detailed discription in Introduction, Results, Discussion, and Materials and Methods, although the authors have to make sure about the order of the numbering of the Figures and to improve the Figures. For the latter, high-resolution Figures and detailed descriptions as Figure legends have to be presented, because it is difficult to recognize about the contents and construction with viewing the figures.

Page Line Comments
Page 1 Line 7 On the description for "F. × ananassa", a space have to be included between "×" and "ananassa".
Page 4 Line 25 Here it can be seen that some redundant descriptions between the current paragraph and the last paragraph. Please present about clear objective in this study.

Page 7 Line 18 Number of the Figures are not matched with the actually presented. The number of the Fig. 2 and Fig. 3 should be converted because Fig.2 is a set of photographs about the flower/fruit developmental stages but not synteny with P. micrantha and the F. vesca genome sequence.
Page 8 Line 20 The authors did not present the results of the two method, ML and MP, although they presented the results NJ in Fig.5, right? If so, please mention about the actual situation with the word "data not shown for ML and MP" for better understanding.

Page 15 Line 6 Why the authors have selected the plants from Serbia?
Page 16 Line 20 The descriptions are redundant in the part of sequencing with HiSeq2000. please correct.
Page 19 Line 15-16 How have the authors discriminated for the two sentences; "FPKM was used to distinguish expressed genes from inactive genes during the flower development in each species." and "FPKM was used to define a set of highly expressed genes."
Page 19 Line 25 It is necessarily to insert a space into sqrt(MSR).
Page 20 Line 12 Its better to include abbreviations for the three methods, Maximum Likelihood (ML), Maximum Parsimony (MP) and Neighbour-joining (NJ)

Resolution of the most of the figures should be improved because it is difficult to recognize about the contents details in the figures with low quality.
Fig. 3 Text size is too small. improve the resolution of the figure
Fig. 4 Text size is too small. improve the resolution of the figure
Fig. 5 The authors have to present explanations about the circular heatmap, gene, the meaning of the A, B, C, and D in the Fig. 5 and Fig 6. Please also present the meaning of the black circle on the dendrogram. I could not recognize about the text in the Fig. 5 because the size is too small.

Methods

Are the methods appropriate to the aims of the study, are they well described, and are necessary controls included? Yes

Conclusions

Are the conclusions adequately supported by the data shown? Yes

Reporting Standards

Does the manuscript adhere to the journal's guidelines on [minimum standards of reporting?]() Yes

Statistics

Are you able to assess all statistics in the manuscript, including the appropriateness of statistical tests used? Yes, and I have assessed the statistics in my report.

Quality of Written English

Please indicate the quality of language in the manuscript: Acceptable

Declaration of Competing Interests

Please complete a declaration of competing interests, considering the following questions:

- Have you in the past five years received reimbursements, fees, funding, or salary from an organisation that may in any way gain or lose financially from the publication of this manuscript, either now or in the future?
- Do you hold any stocks or shares in an organisation that may in any way gain or lose financially from the publication of this manuscript, either now or in the future?
- Do you hold or are you currently applying for any patents relating to the content of the manuscript?
- Have you received reimbursements, fees, funding, or salary from an organization that holds or has applied for patents relating to the content of the manuscript?
- Do you have any other financial competing interests?
- Do you have any non-financial competing interests in relation to this paper?

If you can answer no to all of the above, write 'I declare that I have no competing interests' below. If your reply is yes to any, please give details below.

I declare that I have no competing interests.

I agree to the open peer review policy of the journal. I understand that my name will be included on my report to the authors and, if the manuscript is accepted for publication, my named report including any attachments I upload will be posted on the website along with the authors' responses. I agree for my report to be made available under an Open Access Creative Commons CC-BY license (http://creativecommons.org/licenses/by/4.0/). I understand that any comments which I do not wish to be included in my named report can be included as confidential comments to the editors, which will not be published.

I agree to the open peer review policy of the journal

To further support our reviewers, we have joined with Publons, where you can gain additional credit to further highlight your hard work (see: https://publons.com/journal/530/gigascience). On publication of this paper, your review will be automatically added to Publons, you can then choose whether or not to claim your Publons credit. I understand this statement.

Yes
