# Supplementary material for: The genome sequence and transcriptome of Potentilla micrantha and their comparison to Fragaria vesca (the woodland strawberry)
Source: Gigascience. 2017 Feb 15;7(4):giy010. doi: 10.1093/gigascience/giy010 (PMC5893959; doi:10.1093/gigascience/giy010)
Supplement: Reviewer_3_Report_(Original_Submission) -- Sook Jung — 15 Aug 2017 Reviewed [file giy010_reviewer_3_report_(original_submission).pdf]

Reviewer Report

Title: The genome sequence and transcriptome of Potentilla micrantha and their comparison to Fragaria vesca (the woodland strawberry)

Version: Original Submission **Date:** 15 Aug 2017

Reviewer name: Sook Jung

Reviewer Comments to Author:

The paper titled "The genome sequence and transcriptome of Potentilla micrantha shed light on the origins of strawberry fruit development" reports the new genome and transcriptome data of Potentilla micrantha, a species that belongs to the genus Potentilla that is closely related to that of Fragaria. The work is important for the researchers who are interested in the mechanism of fruit development in Fragaria. However, I feel that some of the conclusions and claims made in the manuscript, including the title, cannot be justified by the data. Below are two main examples.
1) Synteny analysis
The scaffolds have not assembled into a larger chromosome, so the macrosynteny conservation could not be evaluated as stated in the methods section. In the results section, however, authors state that "Comparison of the two genomes revealed a remarkable degree of synteny with P. micrantha scaffolds spanning the entirety of the F. vesca genome sequence". The authors need to clarify that the level of synteny detected is micro-level throughout the manscript, not just in Methods section. The resolution of the Figure 3 (Figure 2?) is too low to see any letters but it looks like a couple of hundreds scaffolds were anchored to each F. vesca chromosome. There is no evidence that majority of these scaffolds aligned to one chromosome is in the same P. micrantha chromosome.
2) RNA-seq data
The authors reported 1,556 differentially expressed genes between the four developmental stages in P. micrantha, and 816 in F. vesca. They did GO annotation on these genes but they didn't seem to have looked any further. Were there any difference in the profile of the differentially expressed genes in both species and that only in F. vesca?

In conclusion, a lot more work needs to be done (anchoring scaffolds to chromosomes to assess the level of macrosynteny and do a further analyses to develop at least some hypotheses on the genetic mechanism of the fruit development). On the other hand, I feel this work is worthwhile to be published as long as the title and text is appropriately edited to reflect the results accurately.

Methods

Are the methods appropriate to the aims of the study, are they well described, and are necessary controls included? Yes

Conclusions

Are the conclusions adequately supported by the data shown? No

Reporting Standards

Does the manuscript adhere to the journal's guidelines on minimum standards of reporting? Yes

Statistics

Are you able to assess all statistics in the manuscript, including the appropriateness of statistical tests used? No, I do not feel adequately qualified to assess the statistics.

Quality of Written English

Please indicate the quality of language in the manuscript: Acceptable

Declaration of Competing Interests

Please complete a declaration of competing interests, considering the following questions:

- Have you in the past five years received reimbursements, fees, funding, or salary from an organisation that may in any way gain or lose financially from the publication of this manuscript, either now or in the future?
- Do you hold any stocks or shares in an organisation that may in any way gain or lose financially from the publication of this manuscript, either now or in the future?
- Do you hold or are you currently applying for any patents relating to the content of the manuscript?
- Have you received reimbursements, fees, funding, or salary from an organization that holds or has applied for patents relating to the content of the manuscript?
- Do you have any other financial competing interests?
- Do you have any non-financial competing interests in relation to this paper?

If you can answer no to all of the above, write 'I declare that I have no competing interests' below. If your reply is yes to any, please give details below.

I declare that I have no competing interests

I agree to the open peer review policy of the journal. I understand that my name will be included on my report to the authors and, if the manuscript is accepted for publication, my named report including any attachments I upload will be posted on the website along with the authors' responses. I agree for my report to be made available under an Open Access Creative Commons CC-BY license (http://creativecommons.org/licenses/by/4.0/). I understand that any comments which I do not wish to

be included in my named report can be included as confidential comments to the editors, which will not be published.

I agree to the open peer review policy of the journal

To further support our reviewers, we have joined with Publons, where you can gain additional credit to further highlight your hard work (see: https://publons.com/journal/530/gigascience). On publication of this paper, your review will be automatically added to Publons, you can then choose whether or not to claim your Publons credit. I understand this statement.

Yes
